# Genotypic and phenotypic spectra of hemojuvelin mutations in primary hemochromatosis patients: a systematic review

**DOI:** 10.1186/s13023-019-1097-2

**Published:** 2019-07-08

**Authors:** Xiaomu Kong, Lingding Xie, Haiqing Zhu, Lulu Song, Xiaoyan Xing, Wenying Yang, Xiaoping Chen

**Affiliations:** 1 Department of Endocrinology, China-Japan Friendship Hospital, No. 2 Yinghua East Street, Chaoyang District, Beijing, 100029 China; 2 Department of Endocrinology and Metabolism, China Meitan General Hospital, No. 29 Xibahe Nanli, Chaoyang District, Beijing, 100029 China

**Keywords:** Iron overload, Hereditary hemochromatosis, Juvenile hemochromatosis, Hemojuvelin

## Abstract

Hereditary hemochromatosis (HH) is a genetic disorder that causes excess absorption of iron and can lead to a variety of complications including liver cirrhosis, arthritis, abnormal skin pigmentation, cardiomyopathy, hypogonadism, and diabetes. Hemojuvelin (*HJV*) is the causative gene of a rare subtype of HH worldwide. This study aims to systematically review the genotypic and phenotypic spectra of *HJV*-HH in multiple ethnicities, and to explore the genotype–phenotype correlations. A comprehensive search of PubMed database was conducted. Data were extracted from 57 peer-reviewed original articles including 132 cases with *HJV*-HH of multiple ethnicities, involving 117 biallelic cases and 15 heterozygotes. Among the biallelic cases, male and female probands of Caucasian ancestry were equally affected, whereas males were more often affected among East Asians (*P*=1.72×10^-2^). Hepatic iron deposition and hypogonadism were the most frequently reported complications. Hypogonadism and arthropathy were more prevalent in Caucasians than in East Asians (*P*=9.30×10^-3^, 1.69×10^-2^). Among the recurrent mutations, G320V (45 unrelated cases) and L101P (7 unrelated cases) were detected most frequently and restricted to Caucasians. [Q6H; C321*] was predominant in Chinese patients (6 unrelated cases). I281T (Chinese and Greek), A310G (Brazilian and African American), and R385* (Italian and North African) were reported across different ethnicities. In genotype–phenotype correlation analyses, 91.30% of homozygotes with exon 2-3 mutations developed early-onset HH compared to 66.00% of those with exon 4 mutations (*P*=2.40×10^-2^). Hypogonadism occurred more frequently in homozygotes with missense mutations (72.55%) than in those with nonsense mutations (35.71%; *P*=2.43×10^-2^). Liver biopsy was accepted by more probands with frame-shift or missense mutations (85.71% and 60.78%, respectively) than by those with nonsense mutations (28.57%; *P*=2.37×10^-2^, 3.93×10^-2^). The present review suggests that patients’ ethnicity, geographical region, and genetic predisposition should be considered in the diagnosis, prognosis and management of *HJV*-HH.

**Electronic supplementary material:**

The online version of this article (10.1186/s13023-019-1097-2) contains supplementary material, which is available to authorized users.

## Background

Hereditary hemochromatosis (HH) is a genetic disorder characterized by disturbed regulation of iron, which leads to systemic iron overload and a wide spectrum of serious complications including cardiomyopathy, liver cirrhosis, arthritis, and skin pigmentation, as well as endocrine disorders such as diabetes, hypogonadotropic hypogonadism, hypothyroidism, etc. [1, 2]. To date, five genes, *HFE*, *HAMP*, *HJV*, *TFR2*, and *SLC40A1,* have been identified as causative genes for various HH subtypes [2, 3].

The hemojuvelin gene (*HJV*) maps to chromosome 1q21.1, and its coding sequence consists of three exons (exon 2, 3 and 4). *HJV* encodes a co-receptor of bone morphogenetic proteins (BMPs) that regulates the circulating level of hepcidin, the principal hormone in the mediation of iron homeostasis [4]. Pathogenic mutations in the *HJV* gene cause hemochromatosis in an autosomal recessive hereditary pattern. Mutation of this gene was first identified in Caucasian families with primary hemochromatosis in 2004 [5]. A previous study based on public databases indicated that the homozygous or compound heterozygous status of *HJV* pathogenic mutations is estimated to cause iron overload in approximately 1 in 5–6 million people worldwide [3]. Biallelic *HJV* mutations were estimated to cause up to 90% of the juvenile hemochromatosis (JH, also known as type 2 HH), which is the most severe HH form with an onset before 30 years of age [4]. If not appropriately treated, JH can lead to death from hemochromatosis-related cardiomyopathy [6]. In addition to the autosomal recessive mode of inheritance, studies in recent years also reported that *HJV* heterozygotes can develop middle age-onset hemochromatosis [7–12]. The phenotypic spectrum and the outcomes in *HJV*-HH cases are of special interest for clinicians, as this information could better guide the diagnosis, prognosis and management of the patients.

During the past 15 years, more than 70 genetic variants related to *HJV*-HH were identified in patients with iron-overload, including non-synonymous, non-sense, frame-shift and in-frame mutations in the coding region of exons 2–4, as well as mutations in the 5′ untranslated region (UTR) and intron. About two-thirds of the mutations were identified in a single proband or family, and recurrent mutations also were detected. Most of the recurrent mutations were restricted to the given race and origin of the family and were diverse and distinct among geographic regions around the world. For example, G320V, the most commonly reported mutation in *HJV*, has been frequently reported in Caucasians (mainly in the North European population), but has never been detected in East Asians [13, 14]. C321*, the most frequently reported mutation in Chinese patients, has not been found in patients of any other race [13]. Given such a wide spectrum of mutations, diagnosis of *HJV*-HH can be challenging. Therefore, the phenotypic variations with respect to ethnicity and genotype need to be investigated in the pursuit for more personalized screening and management strategies for *HJV*-HH.

In the present review, we complied cases of *HJV*-HH published in peer-reviewed journals worldwide in order to summarize the genotypic and phenotypic spectra of *HJV*-HH for multiple ethnicities, as well as the outcomes, for the purpose of improving the current understanding of the disease. This review also provides a comparison of phenotypes between Caucasian and East Asian patients, along with a comparison between early-onset and late-onset *HJV*-HH. All *HJV* mutations described to date were reviewed in terms of ethnic and geographic association. Genotype–phenotype correlations were explored. As *HJV*-HH is a rare hereditary disease in both Caucasians and non-Caucasians, this review provides valuable information for identifying *HJV*-related iron overload both before and after genetic testing.

## Materials and methods

### Literature search strategy

To identify all published HH cases that were genetically confirmed to be *HJV* pathogenic mutation cases (OMIM *608374), we conducted an extensive literature review, which included a comprehensive search of the National Center for Biotechnology Information PubMed database (http://www.ncbi.nlm.nih.gov/pubmed) for articles published through March 20, 2019. The search strategy consisted of multiple queries combining “hemojuvelin” or “HJV” or “HFE2” or “Hemochromatosis, type 2” or “juvenile hemochromatosis” without a language restriction. A list of the search terms employed in the database searches is presented in Additional file 1. A manual search of references from articles including relevant review articles, systematic reviews and meta-analyses was performed. The titles and abstracts of the 546 generated articles were carefully screened to remove the obviously ineligible studies.

Two investigators independently assessed the eligibility of the remaining studies by reviewing their full text. Only peer-reviewed publications providing both genotype and phenotype data related to *HJV* hemochromatosis were included in this review. Finally, 57 articles were included for further investigation. A list of the excluded articles with the associated reasons for exclusion is provided in Additional file 2.

### Duplicate exclusion

To minimize the bias through reporting of the same case from multiple publications, we used the mutation information (both cDNA and protein change), sex, age at onset, serum ferritin (SF) and transferrin saturation (TS) measurements to identify duplicated cases. When a case was included in more than one article, we included only the publication with more detailed case information in the analysis, and all reference was assigned to the individual. Seven cases were found to be reported in more than one publication.

### Eligibility assessment

Among the 167 cases with primary iron overload reported by the 57 eligible publications, six cases that also had genetically diagnosed alpha-thalassemia, beta-thalassemia or congenital dyserythropoietic anemia II were excluded [7, 15–18], and four cases with unavailable sex information were excluded [5]. An additional 25 cases with compound heterozygous or homozygous status of pathogenic mutations in other HH genes (e.g., C282Y, H63D, S65C of *HFE* gene) were removed [8, 9, 19–28]. Thus, 132 eligible iron overload cases were included for data extraction.

### Data extraction

Using a standardized data collection form, the following data from the eligible cases regarding the genetic, phenotypic, demographic and clinical outcomes of individual patients were manually extracted and compiled in a database: genotype of *HJV* and other HH-related genes, sex, age at diagnosis, age at presentation, SF, TS, heart disease, skin hyperpigmentation (including freckled skin), arthropathy, hypogonadism, diabetes or glucose intolerance, osteopathy, thyroid abnormality, abnormality on liver function test, liver iron deposition, liver fibrosis or cirrhosis, therapies and outcomes. Early-onset was defined by an age at presentation of 30 years or younger; all other ages were considered late-onset. If the age at presentation was not provided, the age at diagnosis was used for classification as early-onset or late-onset. The pathogenicity of the reported *HJV* variants was assessed based on the available phenotypic data and the opinions of the study authors. All data were extracted and verified by two investigators.

### Annotation and prediction of the pathogenicity of variants

The variants were mapped to the reference genome (GRCh37/hg19 and GRCh38/hg38) and annotated in the following public databases: 1000 genome (http://www.internationalgenome.org/), Exome Sequencing Project (ESP; Exome Variant Server, NHLBI GO ESP, Seattle, WA; http://evs.gs.washington.edu/EVS/), and Exome Aggregation Consortium (ExAC; Cambridge, MA; http://exac.broadinstitute.org/) data sets. Two computational algorithms, PROVEAN v1.1.3 (http://provean.jcvi.org/index.php) and PolyPhen2 (http://genetics.bwh.harvard.edu/pph2/), were applied to predict the deleterious effects of the missense mutations.

### Nomenclature

The variants were named according to the GenBank *HJV* reference sequences NM_213653.3 and NP_998818.1 (http://www.ncbi.nlm.nih.gov), following the guideline provided by the Human Genome Variation Society (http://www.hgvs.org/).

### Statistical analysis

Statistical analyses were performed using SAS (version 9.2; SAS Institute, Cary, NC, USA). Differences among continuous data were analyzed using factorial analysis of variance (ANOVA) or Student’s t-test. Non-parametric tests were utilized when the normality assumption was not met. Differences among categorical variables were analyzed using chi-squared test of independence or Fisher's exact test.

## Results

### Literature search and inclusion of cases

**Fig. 1:**
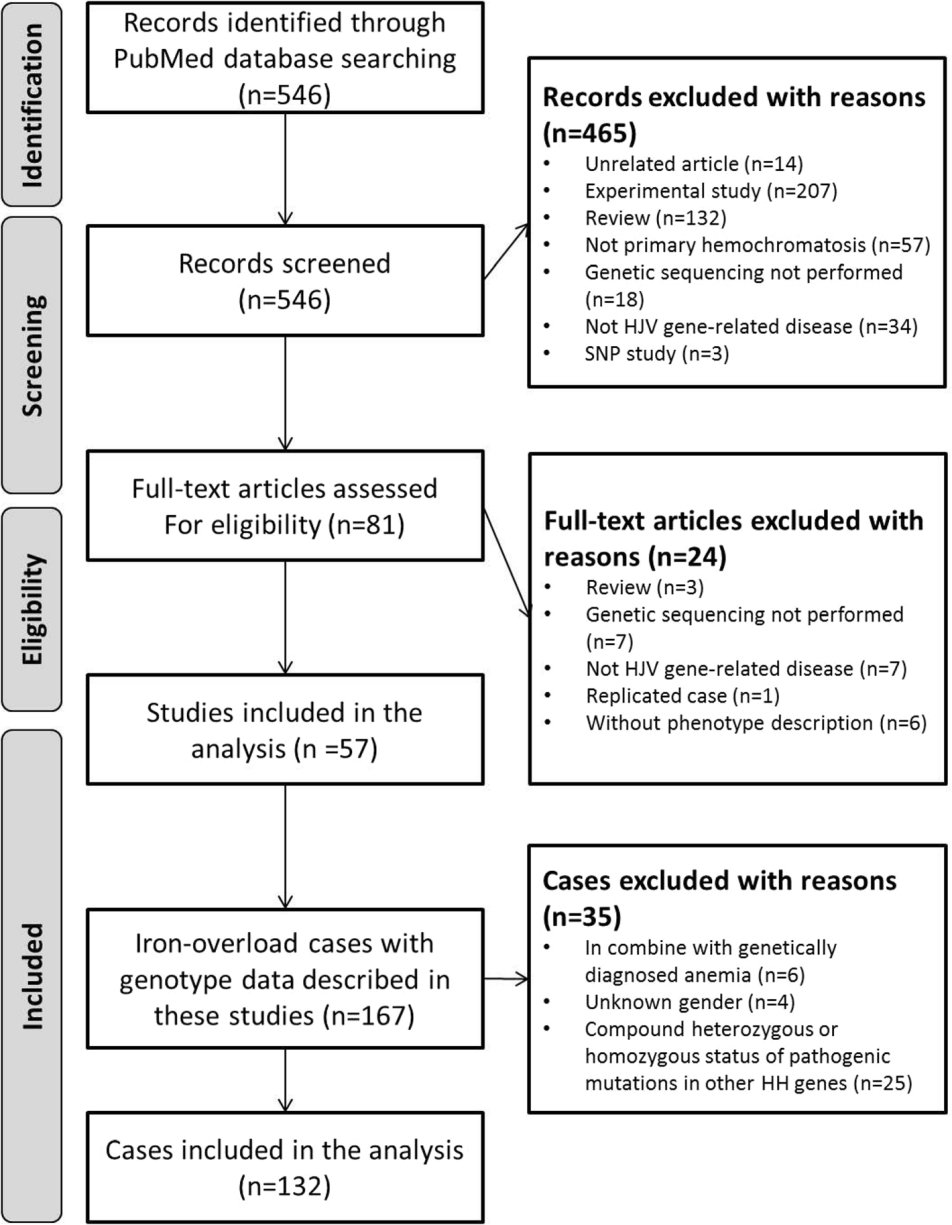
Flow chart of literature search and study selection

The process used to search the literature and identify eligible studies is described in the flow chart in Fig. 1. The search strategy initially identified 546 unique articles. Based on screening of titles and abstracts, 81 were kept for further evaluation. On examination of the full text, 57 articles met the inclusion criteria, and a total of 132 cases were eligible for data extraction. These cases included 117 cases of biallelic mutation and 15 cases with heterozygous mutation. The clinical characteristics of all 132 *HJV*-variant cases are presented in Table 1, and the extracted information for each patient was collected in Additional files 3 and 4.

**Table 1:** Clinical characteristics of the included *HJV*-HH cases

	*HJV*-HH Cases with biallelic mutations			*HJV*-HH Cases with a single mutation			*P*
	All	Probands	Non-probands	All	Probands	Non-probands	
N	117	97	20	15	9	6	
Male, n (%)	61 (52.14)	52 (53.61)	9 (45.00)	10 (66.67)	5 (55.56)	5 (83.33)	9.11 × 10^-1^_C_
Ethnicities Caucasian/East Asian/African/ND, (%)	98/18/1/0 (83.76/15.38/8.55/0.00)	83/13/1/0 (85.57/13.40/1.03/0.00)	15/5/0/0 (75.00/25.00/0.00/0.00)	3/9/0/3 (20.00/60.00/0.00/20.00)	2/4/0/3 (22.22/44.44/0.00/33.33)	1/5/0/0 (16.67/83.33/0.00/0.00)	**6.71 × 10** ^**-3**^_**F**_^a^
Homozygotes, n (%)	89 (76.07)	73 (75.26)	16 (80.00)	—	—	—	—
Age at diagnosis (year)	25.00 (20.00, 32.00)	26.00 (21.00, 32.00)	21.00 (16.00, 28.50)	53.00 (36.00, 65.00)	58.00 (36.00, 62.00)	48.00 (46.00, 70.00)	**8.02 × 10** ^**-4**^_**W**_
Age at presentation (year)	21.50 (18.00, 28.00)	22.50 (19.50, 27.50)	16.50 (13.00, 32.00)	—	—	—	—
Disease onset before 30 years, n (%)	88 (75.21)	72 (74.23)	16 (80.00)	4 (26.67)	3 (33.33)	1 (16.67)	**1.75 × 10** ^**-2**^_**F**_
Serum parameters at presentation							
Serum ferritin (ng/ml)	3541.80 (2270.00, 5293.00)	3700.00 (2329.00, 5520.00)	1971.50 (1117.50, 4314.00)	444.00 (356.00, 1402.00)	784.50 (388.50, 1512.50)	382.50 (266.00, 554.00)	**2.70 × 10** ^**-5**^_**W**_
Transferrin saturation (%)	94.00 (90.00, 100.00)	95.00 (89.00, 100.00)	92.00 (90.00, 97.00)	49.00 (40.00, 68.00)	64.00 (47.00, 83.00)	40.00 (34.00, 46.00)	**1.03 × 10** ^**-4**^_**W**_
Complications							
Cardiomyopathy, n (%)	40 (34.19)	36 (37.11)	4 (20.00)	1 (6.67)	1 (11.11)	0 (0.00)	1.57 × 10^-1^_F_
Skin hyperpigmentation, n (%)	48 (41.03)	40 (41.24)	8 (40.00)	2 (13.33)	1 (11.11)	1 (16.67)	1.49 × 10^-1^_F_
Arthropathy, n (%)	32 (27.35)	27 (27.84)	5 (25.00)	1 (6.67)	1 (11.11)	0 (0.00)	4.40 × 10^-1^_F_
Endocrine abnormality							
Hypogonadism, n (%)	70 (59.83)	63 (64.95)	7 (35.00)	2 (13.33)	1 (11.11)	1 (16.67)	**2.95 × 10** ^**-3**^_**F**_
Glucose intolerance, n (%)	36 (30.77)	33 (34.02)	3 (15.00)	2 (13.33)	1 (11.11)	1 (16.67)	2.66 × 10^-1^_F_
Osteopathy, n (%)	11 (9.40)	7 (7.22)	4 (20.00)	0 (0.00)	0 (0.00)	0 (0.00)	1.00 × 10^-1^_F_
Thyroid abnormality, n (%)	9 (7.69)	7 (7.22)	2 (10.00)	0 (0.00)	0 (0.00)	0 (0.00)	1.00 × 10^-1^_F_
Liver disease							
Abnormal liver function test, n (%)	39 (33.33)	35 (36.08)	4 (20.00)	4 (26.67)	3 (33.33)	1 (16.67)	1.00 × 10^-1^_F_
Liver iron deposition, n (%)	74 (63.25)	67 (69.07)	7 (35.00)	4 (26.67)	4 (44.44)	0 (0.00)	1.53 × 10^-1^_F_
Liver fibrosis, n (%)	43 (36.75)	39 (40.21)	4 (20.00)	1 (6.67)	1 (11.11)	0 (0.00)	1.49 × 10^-1^_F_
Liver cirrhosis, n (%)	28 (23.93)	26 (26.80)	2 (10.00)	1 (6.67)	1 (11.11)	0 (0.00)	4.43 × 10^-1^_F_
Liver biopsy, n (%)	62 (52.99)	56 (57.73)	6 (30.00)	3 (20.00)	3 (33.33)	0 (0.00)	1.81 × 10^-1^_F_
Therapy Phlebotomy/Chelating agent/Phlebotomy & Chelating agent/ND, n (%)	60/3/7/47 (51.28/2.56/5.98/40.17)	46/3/7/41 (47.42/3.09/7.22/42.27)	14/0/0/6 (70.00/0.00/0.00/30.00)	3/0/0/12 (20.00/0.00/0.00/80.00)	2/0/0/7 (22.22/0.00/0.00/77.78)	1/0/0/5 (16.67/0.00/0.00/83.33)	—

*Abbreviations*: *HJV*-HH *HJV*-related hereditary hemochromatosis, *ND* not described

Data are shown as n (%) or median (interquartile range). *P* values were calculated to assess the differences between probands with biallelic mutations and probands with a single mutation using chi-square test, Fisher’s exact test or Wilcoxon test as appropriate. _C_, on chi-square test; _F_, on Fisher’s exact test; _W_, on Wilcoxon test. ^a^, compared the proportions of Caucasians and East Asians. *P* values <0.05 are denoted in bold and underlined

### Phenotypic spectrum of the biallelic *HJV* mutation cases

As shown in Table 1, 97 of the 117 cases with biallelic mutations were the probands of their family with comparable proportions of males (53.61%) and females (46.39%). Of the probands, 83 were Caucasians, 13 were East Asians, and one was African. Seventy-three of the probands had homozygous mutation, and 24 had compound heterozygous mutation. Presentation of iron overload or its related traits occurred at age 30 years or earlier in 72 probands, whereas 25 cases were late-onset. Notably, hepatic iron deposition and hypogonadism were the most frequently reported complications. Moreover, more than one-third of cases had complications including liver fibrosis, skin hyperpigmentation, abnormal liver function test results, cardiomyopathy, and impaired glucose regulation. Arthropathy, liver cirrhosis, osteopathy, and thyroid abnormality were also reported.

We further compared the phenotypes of the early-onset and late-onset probands (Table 2). The early-onset probands showed a significantly higher prevalence of hypogonadism (75.00%) than the late-onset probands (36.00%; *P*=4.30×10^-4^). Liver iron deposition was more frequently detected in late-onset cases (96.00%) and was significantly higher in these cases than in the early-onset ones (59.72%; *P*=7.22×10^-4^). A greater proportion of late-onset cases developed glucose intolerance (including diabetes; 48.00%) compared with that among early-onset cases (29.17%), but the difference was not statistically significant (*P*=8.68×10^-2^).

**Table 2:** Comparisons of the clinical characteristics of early-onset versus late-onset *HJV*-HH cases with biallelic mutations

	Probands			Non-probands		
	Onset age ≤30 years	Onset age >30 years	*P*	Onset age ≤30 years	Onset age >30 years	*P*
N	72	25		16	4	
Male, n (%)	37 (51.39)	15 (60.00)	4.57 × 10^-1^_C_	8 (50.00)	1 (25.00)	5.91 × 10^-1^_F_
Homozygotes, n (%)	54 (75.00)	19 (76.00)	9.21 × 10^-1^_C_	12 (75.00)	4 (100.00)	5.38 × 10^-1^_F_
Age at diagnosis (year)	24.00 (19.50, 27.50)	39.00 (32.00, 49.00)	**1.53 × 10** ^**-12**^_**W**_	19.00 (16.00, 22.50)	45.50 (36.00, 53.00)	**2.86 × 10** ^**-3**^_**W**_
Age at presentation (year)	21.00 (18.00, 25.00)	33.50 (32.00, 39.00)	**7.10 × 10** ^**-7**^_**W**_	15.00 (12.00, 17.00)	34.00 (32.00, 49.00)	**2.18 × 10** ^**-2**^_**W**_
Serum parameters at presentation						
Serum ferritin (ng/ml)	3520.90 (2291.50, 5782.50)	3987.00 (2680.00, 4959.00)	6.16 × 10^-1^_W_	1971.50 (1117.50, 3498.50)	2753.50 (869.50, 6981.50)	9.52 × 10^-1^_W_
Transferrin saturation (%)	95.50 (89.00, 100.00)	93.50 (89.00, 99.50)	4.73 × 10^-1^_W_	92.00 (90.00, 98.00)	92.00 (75.00, 94.90)	5.46 × 10^-1^_W_
Complications						
Cardiomyopathy, n (%)	29 (40.28)	7 (28.00)	2.74 × 10^-1^_C_	3 (18.75)	1 (25.00)	1.00 × 10^-0^_F_
Skin hyperpigmentation, n (%)	29 (40.28)	11 (44.00)	7.45 × 10^-1^_C_	6 (37.50)	2 (50.00)	1.00 × 10^-0^_F_
Arthropathy, n (%)	18 (25.00)	9 (36.00)	2.90 × 10^-1^_C_	4 (25.00)	1 (25.00)	1.00 × 10^-0^_F_
Endocrine abnormality						
Hypogonadism, n (%)	54 (75.00)	9 (36.00)	**4.30 × 10** ^**-4**^_**C**_	6 (37.50)	1 (25.00)	1.00 × 10^-0^_F_
Glucose intolerance, n (%)	21 (29.17)	12 (48.00)	8.68 × 10^-2^_C_	2 (12.50)	1 (25.00)	5.09 × 10^-1^_F_
Osteopathy, n (%)	6 (8.33)	1 (4.00)	6.73 × 10^-1^_F_	4 (25.00)	0 (0.00)	5.38 × 10^-1^_F_
Thyroid abnormality, n (%)	4 (5.56)	3 (12.00)	3.69 × 10^-1^_F_	3 (18.75)	1 (25.00)	1.00 × 10^-0^_F_
Liver disease						
Abnormal liver function test, n (%)	23 (31.94)	12 (48.00)	1.50 × 10^-1^_C_	3 (18.75)	1 (25.00)	1.00 × 10^-0^_F_
Liver iron deposition, n (%)	43 (59.72)	24 (96.00)	**7.22 × 10** ^**-4**^_**C**_	6 (37.50)	1 (25.00)	1.00 × 10^-0^_F_
Liver fibrosis, n (%)	31 (43.06)	8 (32.00)	3.31 × 10^-1^_C_	3 (18.75)	1 (25.00)	1.00 × 10^-0^_F_
Liver cirrhosis, n (%)	20 (27.78)	6 (24.00)	7.13 × 10^-1^_C_	1 (6.25)	1 (25.00)	3.68 × 10^-1^_F_
Liver biopsy, n (%)	41 (56.94)	15 (60.00)	7.90 × 10^-1^_C_	5 (31.25)	1 (25.00)	1.00 × 10^-0^_F_
Therapy Phlebotomy/Chelating agent/Phlebotomy & Chelating agent/ND, n (%)	28/2/6/36 (38.89/2.78/8.33/50.00)	18/1/1/5 (72.00/4.00/4.00/20.00)	—	11/0/0/5 (68.75/0.00/0.00/31.25)	3/0/0/1 (75.00/0.00/0.00/25.00)	—

*Abbreviations*: *HJV*-HH *HJV*-related hereditary hemochromatosis, *ND* not described

Data are shown as n (%) or median (interquartile range). *P* values were calculated to assess the differences between cases with onset age ≤30 years and cases with onset age >30 years among probands or non-probands using chi-square test, Fisher’s exact test or Wilcoxon test as appropriate. _C_, on chi-square test; _F_, on Fisher’s exact test; _W_, on Wilcoxon test. *P* values <0.05 are denoted in bold and underlined

### Comparisons of phenotypes between Caucasian and East Asian probands with biallelic *HJV* mutation

In Caucasian probands with biallelic *HJV* mutation, similar proportions of male (48.19%) and female (51.81%) were observed, whereas the proportion of male cases (84.62%) was significantly greater among East Asians (*P*=1.72×10^-2^). The age at diagnosis of iron overload and the age at the first presentation of symptoms, as well as the pretreatment levels of SF and TS, were comparable between the populations. Notably, hypogonadism was diagnosed in 71.08% of the Caucasian probands, but in only 33.33% of the East Asians patients (*P*=9.30×10^-3^). Also, 32.53% of the Caucasian probands, but none of the East Asian probands, complained of arthralgia or were diagnosed with arthropathy such as arthritis (*P*=1.69×10^-2^). In addition, East Asian probands showed higher prevalence rates of complications including liver iron deposition, abnormal liver function test results, glucose intolerance, and cardiomyopathy than did Caucasian probands, whereas osteopathy and thyroid involvement were only reported in Caucasian probands. However, the prevalence rates for these complications were not statistically different between the populations (Table 3).

**Table 3:** Comparisons of the clinical characteristics of Caucasian versus East Asian *HJV*-HH cases with biallelic mutations

	Probands			Non-probands		
	Caucasians	East Asians	*P*	Caucasians	East Asians	*P*
N	83	13		15	5	
Male, n (%)	40 (48.19)	11 (84.62)	**1.72 × 10** ^**-2**^_**F**_	6 (40.00)	3 (60.00)	6.17 × 10^-1^_F_
Homozygotes, n (%)	64 (77.11)	8 (61.54)	3.00 × 10^-1^_F_	12 (80.00)	4 (80.00)	1.00 × 10^-1^_F_
Age at diagnosis (year)	26.00 (21.00, 32.00)	26.00 ( 19.00, 37.00)	6.72 × 10^-1^_W_	20.00 ( 16.00, 24.00)	27.00 ( 22.00, 51.00)	8.03 × 10^-2^_W_
Age at presentation (year)	22.50 (20.00, 27.00)	21.50 ( 14.00, 48.00)	8.73 × 10^-1^_W_	—	—	—
Disease onset before 30 years, n (%)	63 (75.90)	8 (61.54)	3.13 × 10^-1^_F_	13 (86.67)	3 (60.00)	2.49 × 10^-1^_F_
Serum parameters at presentation						
Serum ferritin (ng/ml)	3553.00 (2329.00, 5293.00)	4974.00 (2337.00,6678.00)	2.21 × 10^-1^_W_	1648.00 (1117.50, 3498.50)	3389.00 (1505.00,5273.50)	6.71 × 10^-1^_W_
Transferrin saturation (%)	95.00 (88.00, 100.00)	94.40 ( 92.05, 97.55)	9.80 × 10^-1^_W_	92.00 (80.00, 98.00)	93.70 ( 91.00, 95.60)	8.01 × 10^-1^_W_
Complications						
Cardiomyopathy, n (%)	29 (34.94)	7 (53.85)	2.26 × 10^-1^_F_	3 (20.00)	1 (20.00)	1.00 × 10^-1^_F_
Skin hyperpigmentation, n (%)	35 (42.17)	5 (38.46)	1.00 × 10^-0^_F_	6 (40.00)	2 (40.00)	1.00 × 10^-1^_F_
Arthropathy, n (%)	27 (32.53)	0 (0.00)	**1.69 × 10** ^**-2**^_**F**_	5 (33.33)	0 (0.00)	2.66 × 10^-1^_F_
Endocrine abnormality						
Hypogonadism, n (%)	59 (71.08)	4 (30.77)	**9.30 × 10** ^**-3**^_**F**_	6 (40.00)	1 (20.00)	6.13 × 10^-1^_F_
Glucose intolerance, n (%)	26 (31.33)	7 (53.85)	1.27 × 10^-1^_F_	1 (6.67)	2 (40.00)	1.40 × 10^-1^_F_
Osteopathy, n (%)	7 (8.43)	0 (0.00)	5.88 × 10^-1^_F_	4 (26.67)	0 (0.00)	5.30 × 10^-1^_F_
Thyroid abnormality, n (%)	7 (8.43)	0 (0.00)	5.88 × 10^-1^_F_	2 (13.33)	0 (0.00)	1.00 × 10^-1^_F_
Liver disease						
Abnormal liver function test, n (%)	28 (33.73)	7 (53.85)	2.17 × 10^-1^_F_	2 (13.33)	2 (40.00)	2.49 × 10^-1^_F_
Liver iron deposition, n (%)	55 (66.27)	11 (84.62)	3.34 × 10^-1^_F_	5 (33.33)	2 (40.00)	1.00 × 10^-1^_F_
Liver fibrosis, n (%)	36 (43.37)	3 (23.08)	2.29 × 10^-1^_F_	4 (26.67)	0 (0.00)	5.30 × 10^-1^_F_
Liver cirrhosis, n (%)	22 (26.51)	4 (30.77)	7.45 × 10^-1^_F_	1 (6.67)	1 (20.00)	4.47 × 10^-1^_F_
Liver biopsy, n (%)	48 (57.83)	8 (61.54)	1.00 × 10^-1^_F_	4 (26.67)	2 (40.00)	6.13 × 10^-1^_F_
Therapy Phlebotomy/Chelating agent/Phlebotomy & Chelating agent/ND, n (%)	41/2/6/34 (49.40/2.41/7.23/40.96)	5/0/1/7 (38.46/0.00/7.69/53.85)	—	12/0/0/3 (80.00/0.00/0.00/20.00)	2/0/0/3 (40.00/0.00/0.00/60.00)	—

*Abbreviations*: *HJV*-HH *HJV*-related hereditary hemochromatosis, *ND* not described

Data are shown as n (%) or median (interquartile range). *P* values were calculated to assess the differences between Caucasians and East Asians using Fisher’s exact test or Wilcoxon test as appropriate. _F_, on Fisher’s exact test; _W_, on Wilcoxon test. *P* values <0.05 are denoted in bold and underlined

### Phenotypic spectrum of the monoallelic *HJV* mutation cases

As shown in Table 1, 15 monoallelic *HJV* mutation-related iron-overload cases were reported, including nine probands (5 men and 4 women) and six of their relatives. Two of the probands were Caucasian, four were East Asian, and three originated from Brazil with no race information provided. Early-onset occurred in only three of the probands. Signs of organ damage other than hepatic iron deposition and abnormal liver function were seldom reported in these patients (Table 1).

The ethnicity distributions were significantly different between the monoallelic mutation probands and biallelic mutation probands (*P*=6.71×10^-3^). As expected, the SF and TS levels at presentation for the monoallelic mutation probands were significantly lower than those for biallelic mutation probands (*P*=2.70×10^-5^, 1.03×10^-4^). The age at diagnosis among the monoallelic mutation probands was later (*P*=8.02×10^-4^), and more individuals with late-onset were identified (*P*=1.75×10^-2^). Iron overload-related complications were less prevalent in monoallelic mutation cases than in biallelic mutation cases, and this was especially true for hypogonadism (*P*=2.95×10^-3^; Table 1).

### Mutation profiles in different ethnicities

**Fig. 2:**
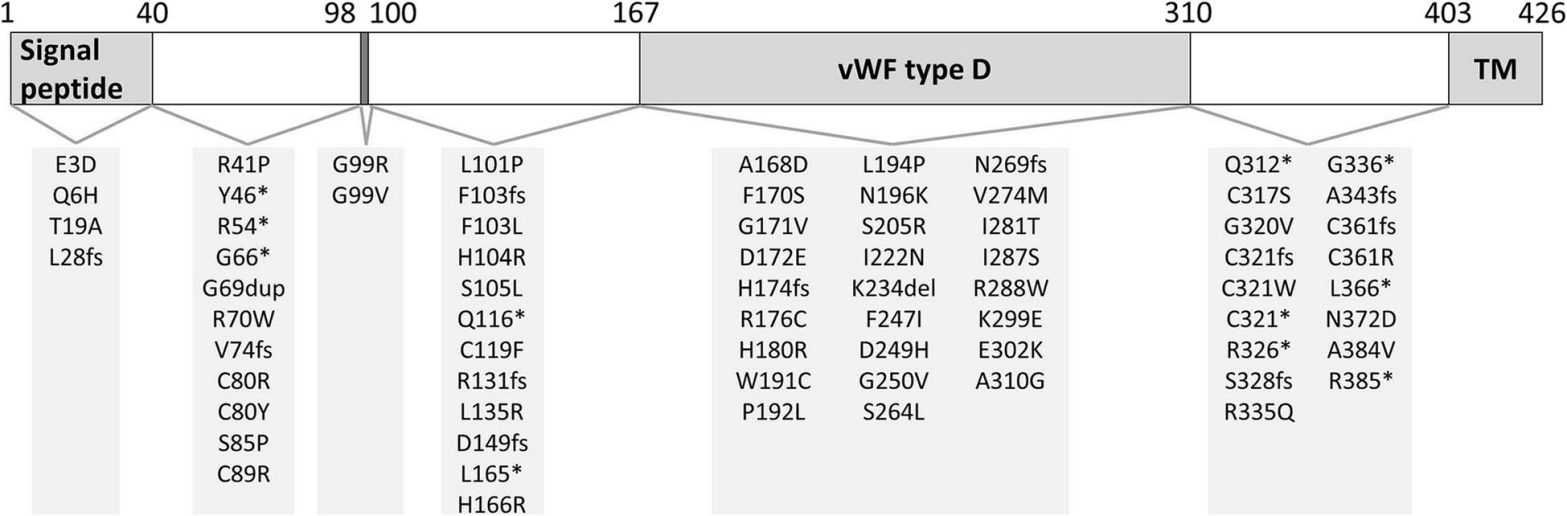
Structure of the HJV protein and mutations in *HJV*. Abbreviations: TM, transmembrane domain; vWF type D: von Willebrand factor type D domain

In total, 72 variants in the coding sequence as well as 1 mutation in the splicing site, 2 mutations in the non-coding region of 5′ UTR, and 1 case of whole gene deletion were reported in *HJV* patients as related to hemochromatosis before March 20, 2019 in publications found in the PubMed database. The locations of the mutations in the *HJV* gene are shown in Fig. 2.

The 72 coding sequence variants included 11 frame-shift mutations, 11 nonsense mutations, 48 missense mutations, 1 deletion, and 1 insertion. Respectively, 4, 37 and 31 mutations were located in exons 2, 3, and 4. Except for A310G, the variants in the coding sequence were either absent in the databases or reported with a very low minor allele frequency.

A detailed list of the information and family origin of each variant is provided in Table 4. Twenty-six of the reported mutations were observed in more than one family. Three mutations were reported across different ethnicities, including I281T (in Chinese and Greek), A310G (in Brazilian and African American), and R385* (in Italian and North African). G320V, L101P, D149fs, E302K, G336*, G99R, and R176C were the most frequently recurrent variations in Caucasians, and each mutation was detected in at least three unrelated probands. In East Asians, *HJV* mutations were reported only in families of Chinese or Japanese origin. [Q6H; C321*] *in cis* was the most frequently observed mutation in Chinese families with hemochromatosis. E3D was identified in five families of Chinese origin. Both Q6H and E3D were predicted as benign by bioinformatics tools. I281T was reported in two families from China. D249H and Q312* were the most frequently reported mutations in Japanese cases, and Q312* was also observed in the Chinese population. R385* and A310G were the commonest variations in the African population, and both were also reported in Caucasians from Brazil or Italy. However, very few cases of African ancestry that underwent genetic diagnosis have been published.

**Table 4:** Information for *HJV* mutations

Nucleotide change	Amino acid change	Coordinates (GRCh37/hg19)	RSID	Allele frequency reported				Bioinformatics prediction of missense variant			Number of reported probands	Family of origin	Ethnicity reported	Reference	Included and excluded cases in the present study ^ref^
				1000G	ESP	ExAC	GnomAD	PolyPhen-2 (HumDiv)	PolyPhen-2 (HumVar)	Provean					
c.9G>C	p.Glu3Asp (p.E3D)	chr1:145414790	rs12025510	3.00×10^-3^	NA	1.25×10^-3^	1.19×10^-3^	Benign	Benign	Neutral	5	China	East Asian	9	Included: Heterozygote: 5 [9]
c.18G>C	p.Gln6His (p.Q6H)	chr1:145414799	rs376970642	NA	7.70×10^-5^	2.47×10^-5^	2.00×10^-5^	Benign	Benign	Neutral	6	China	East Asian	9,10,11,29	Included: Homozygote: 5 [9, 10, 29]; Heterozygote: 1 [11]
c.55A>G	p.Thr19Ala (p.T19A)	chr1:145414836	rs781981862	NA	NA	8.24×10^-6^	8.16×10^-6^	Benign	Benign	Neutral	1	China	East Asian	15	Excluded: Heterozygote: 1 [15]
c.81delG	p.Leu28SerfsTer24 (p.L28fs)	chr1:145414862	NA	NA	NA	NA	NA	—	—	—	1	England/Ireland	Caucasian	30	Included: Homozygote: 1 [30]
c.122G>C	p.Arg41Pro (p.R41P)	chr1:145415303	rs781785800	NA	NA	8.30×10^-6^	4.08×10^-6^	Probably damaging	Probably damaging	Deleterious	1	France	Caucasian	26	Included: Compound heterozygote: 1 [26]
c.138C>A	p.Tyr46Ter (p.Y46*)	chr1:145415319	NA	NA	NA	NA	NA	—	—	—	1	China	East Asian	31	Included: Homozygote: 1 [31]
c.160A>T	p.Arg54Ter (p.R54*)	chr1:145415341	rs121434375	NA	NA	NA	NA	—	—	—	1	African American	African American	17	Excluded: Heterozygote: 1 [17]
c.196G>T	p.Gly66Ter (p.G66*)	chr1:145415377	rs1469129426	NA	NA	NA	NA	—	—	—	2	Czech, Romania:	Caucasian	32,33	Included: Homozygote: 2 [32, 33]
c.204_205insGGA	p.Gly69dup (p.G69dup)	chr1:145415386_145415387insGGA	NA	NA	NA	NA	NA	—	—	—	1	United States (African American)	African American	34	Excluded: Heterozygote: 1 [34]
c.208C>T	p.Arg70Trp (p.R70W)	chr1:145415389	rs377513689	NA	1.54×10^-4^	5.06×10^-5^	3.31×10^-5^	Probably damaging	Possibly damaging	Neutral	1	France	Caucasian	35	Excluded: Heterozygote: 1 [35]
c.220delG	p.Val74TrpfsTer40 (p.V74fs)	chr1:145415401	NA	NA	NA	NA	NA	—	—	—	1	England	Caucasian	18	Included: Compound heterozygote: 1 [18]
c.238T>C	p.Cys80Arg (p.C80R)	chr1:145415419	rs28940586	NA	NA	8.50×10^-6^	4.16×10^-6^	Probably damaging	Probably damaging	Deleterious	2	Southeast United States, Australia	Caucasian	28,36	Included: Compound heterozygote: 2 [28, 36]
c.239G>A	p.Cys80Try (p.C80Y)	chr1: 145415420	NA	NA	NA	NA	NA	Probably damaging	Probably damaging	Deleterious	1	Bangladesh/United Kingdom	Caucasian	37	Included: Compound heterozygote: 1 [37]
c.253T>C	p.Ser85Pro (p.S85P)	chr1:145415434	NA	NA	NA	NA	NA	Probably damaging	Probably damaging	Deleterious	1	Italy	Caucasian	18	Included: Homozygote: 1 [18]
c.265T>C	p.Cys89Arg (p.C89R)	chr1:145415446	NA	NA	NA	NA	NA	Probably damaging	Probably damaging	Deleterious	1	Western Iran	Caucasian	38	Included: Homozygote: 1 [38]
c.295G>A	p.Gly99Arg (p.G99R)	chr1:145415476	NA	NA	NA	NA	NA	Probably damaging	Probably damaging	Deleterious	3	Pakistan, Albania	Caucasian	18,37	Included: Homozygote: 2; Compound heterozygote: 1 [18, 37]
c.296G>T	p.Gly99Val (p.G99V)	chr1:145415477	rs1451187897	NA	NA	NA	NA	Probably damaging	Probably damaging	Deleterious	1	Greece	Caucasian	5	Included: Homozygote: 1 [5]
c.302T>C	p.Leu101Pro (p.L101P)	chr1: 145415483	rs74315327	NA	NA	8.65×10^-6^	8.41×10^-6^	Probably damaging	Probably damaging	Deleterious	7	Southeast United, Albania, France	Caucasian	18,26,27,36	Included: Compound heterozygote: 5 [18, 26, 36] Excluded: Heterozygote: 2 [27]
c.306delC	p.Phe103SerfsTer11 (p.F103fs)	chr1:145415487	NA	NA	NA	NA	NA	—	—	—	1	Italy	Caucasian	7	Included: Heterozygote: 1 [7]
c.309C>G	p.Phe103Leu (p.F103L)	chr1:145415490	NA	NA	NA	NA	NA	Possibly damaging	Benign	Deleterious	1	China	East Asian	9	Included: Homozygote: 1 [9]
c.311A>G	p.His104Arg (p.H104R)	chr1:145415492	NA	NA	NA	NA	NA	Probably damaging	Probably damaging	Deleterious	1	China	East Asian	9	Included: Compound heterozygote: 1 [9]
c.314C>T	p.Ser105Leu (p.S105L)	chr1:145415495	NA	NA	NA	NA	NA	Probably damaging	Probably damaging	Deleterious	1	France	Caucasian	27	Excluded: Heterozygote: 1 [27]
c.346C>T	p.Gln116Ter (p.Q116*)	chr1:145415527	NA	NA	NA	NA	NA	—	—	—	1	Ireland	Caucasian	39	Included: Compound heterozygote: 1 [39]
c.356G>T	p.Cys119Phe (p.C119F)	chr1:145415537	NA	NA	NA	NA	NA	Probably damaging	Probably damaging	Deleterious	1	Germany	Caucasian	40	Included: Homozygote: 1 [40]
c.391_403del	p.Arg131PhefsTer111 (p.R131fs)	chr1:145415572_145415584del	rs1486905702	NA	NA	NA	NA	—	—	—	1	Italy	Caucasian	18	Included: Homozygote: 1 [18]
c.404T>G	p.Leu135Arg (p.L135R)	chr1:145415585	rs782182681	NA	NA	2.75×10^-5^	1.29×10^-5^	Benign	Benign	Neutral	1	Spanish	Caucasian	25	Excluded: Heterozygote: 1 [25]
c.445delG	p.Asp149ThrfsTer97 (p.D149fs)	chr1:145415626	NA	NA	NA	NA	NA	—	—	—	4	Italy	Caucasian	8,18	Included: Homozygote: 2 [18]; Heterozygote: 1 [8] Excluded: Homozygote: 1 [18]
c.494T>A	p.Leu165Ter (p.L165*)	chr1:145415675	rs782431871.	NA	NA	8.36×10^-6^	8.32×10^-6^	—	—	—	2	Netherland	Caucasian	16,21	Included: Compound heterozygote: 2 [16, 21]
c.497A>G	p.His166Arg (p.H166R)	Chr1:145415678	NA	NA	NA	NA	NA	Probably damaging	Probably damaging	Deleterious	1	Arab	Caucasian	41	Included: Homozygote: 1 [41]
c.503C>A	p.Ala168Asp (p.A168D)	chr1:145415684	rs782125244	NA	NA	NA	NA	Probably damaging	Probably damaging	Neutral	1	Australia/England	Caucasian	18	Included: Homozygote: 1 [18]
c.509T>C	p.Phe170Ser (p.F170S)	chr1:145415690	NA	NA	NA	NA	NA	Probably damaging	Probably damaging	Deleterious	2	Italy	Caucasian	18	Included: Homozygote: 2 [18]
c.512G>T	p.Gly171Val (p.G171V)	chr1:145415693	rs782077224	NA	NA	NA	NA	Probably damaging	Probably damaging	Deleterious	NA	Not mentioned	Not mentioned	42	NA [42]
c.516C>G	p.Asp172Glu (p.D172E)	chr1:145415697	rs782708481	NA	NA	8.35×10^-6^	4.16×10^-6^	Probably damaging	Probably damaging	Deleterious	1	Italy	Caucasian	18	Included: Compound heterozygote: 1 [18]
c.515_516insC	p.His174ProfsTer23 (p.H174fs)	chr1:145415696_145415697insC	NA	NA	NA	NA	NA	—	—	—	1	Japan	East Asian	43,44	Included: Homozygote: 1 [43, 44]
c.526C>T	p.Arg176Cys (p.R176C)	chr1:145415707	NA	NA	NA	NA	NA	Probably damaging	Probably damaging	Deleterious	3	France	Caucasian	19,22,26,45	Included: Homozygote: 1 [45] ; Compound heterozygote: 1 [26] Excluded: Compound heterozygote: 1 [19, 22]
c.539A>G	p.His180Arg (p.H180R)	chr1:145415720	rs1395419937	NA	NA	NA	2.07×10^-5^	Benign	Benign	Neutral	1	France	Caucasian	26	Included: Compound heterozygote: 1 [26]
c.573G>T	p.Trp191Cys (p.W191C)	chr1:145415754	NA	NA	NA	NA	NA	Probably damaging	Probably damaging	Deleterious	1	Italy	Caucasian	18	Included: Homozygote: 1 [18]
c.575C>T	p.Pro192Leu (p.P192L)	chr1:145415756	NA	NA	NA	NA	NA	Probably damaging	Probably damaging	Deleterious	1	Pakistan	Caucasian	46	Included: Homozygote: 1 [46]
c.581T>C	p.Leu194Pro (p.L194P)	chr1:145415762	rs782682271	NA	NA	8.34×10^-6^	8.26×10^-6^	Probably damaging	Probably damaging	Deleterious	1	Pakistan	Caucasian	46	Included: Homozygote: 1 [46]
c.588T>G	p.Asn196Lys (p.N196K)	chr1:145415769	rs1020058448	NA	NA	NA	NA	Probably damaging	Probably damaging	Deleterious	1	Italy	Caucasian	8	Excluded: Heterozygote: 1 [8]
c.615C>G	p.Ser205Arg (p.S205R)	chr1:145415796	NA	NA	NA	NA	NA	Probably damaging	Probably damaging	Neutral	1	Italy	Caucasian	18	Included: Compound heterozygote: 1 [18]
c.665T>A	p.Ile222Asn (p.I222N)	chr1:145416320	rs74315325	NA	7.70×10^-5^	8.24×10^-6^	2.44×10^-5^	Probably damaging	Probably damaging	Deleterious	2	Southeast United States, Canada	Caucasian	5,36	Included: Compound heterozygote: 1 [36] Excluded: Compound heterozygote: 1 [5]
c.700_702delAAG	p.Lys234del (p.K234del)	chr1:145416355_145416357delAAG	NA	NA	NA	NA	NA	—	—	—	1	Europe	Caucasian	47	Included: Homozygote: 1 [47]
c.739T>A	p.Phe247Ile (p.F247I)	chr1:145416394	NA	NA	NA	NA	NA	Probably damaging	Probably damaging	Deleterious	1	Turkey	Caucasian	16	Included: Homozygote: 1 [16]
c.745G>C	p.Asp249His (p.D249H)	chr1:145416400	NA	NA	NA	NA	NA	Probably damaging	Probably damaging	Deleterious	2	Japan	East Asian	43,48-50	Included: Homozygote: 2 [43, 48–50]
c.749G>T	p.Gly250Val (p.G250V)	chr1:145416404	rs863224819	NA	NA	NA	3.23×10^-5^	Probably damaging	Probably damaging	Deleterious	1	Italy	Caucasian	18	Included: Compound heterozygote: 1 [18]
c.791C>T	p.Ser264Leu (p.S264L)	chr1:145416446	rs782576713	NA	NA	1.65×10^-5^	2.03×10^-5^	Possibly damaging	Benign	Neutral	1	Spanish	Caucasian	23	Excluded: Heterozygote: 1 [23]
c.806dupA	p.Asn269LysfsTer43 (p.N269fs)	chr1:145416461dupA	NA	NA	NA	NA	NA	—	—	—	1	England	Caucasian	18	Included: Compound heterozygote: 1 [18]
c.820G>A	p.Val274Met (p.V274M)	chr1:145416475	rs187777957	5.99×10^-4^	NA	9.88×10^-5^	8.53×10^-5^	Probably damaging	Probably damaging	Deleterious	1	China	East Asian	9	Included: Compound heterozygote: 1 [9]
c.842T>C	p.Ile281Thr (p.I281T)	chr1:145416497	rs74315326	NA	NA	NA	8.12×10^-6^	Probably damaging	Probably damaging	Deleterious	3	Greece, China	Caucasian, East Asian	5,9,10	Included: Homozygote: 1 [5]; Compound heterozygote: 2 [9, 10]
c.860T>G	p.Ile287Ser (p.I287S)	chr1:145416515	NA	NA	NA	NA	NA	Possibly damaging	Possibly damaging	Deleterious	1	China	East Asian	31	Included: Homozygote: 1 [31]
c.862C>T	p.Arg288Trp (p.R288W)	chr1:145416517	rs782493762	NA	NA	1.65×10^-5^	1.62×10^-5^	Probably damaging	Probably damaging	Deleterious	2	France	Caucasian	18,26,51	Included: Homozygote: 2 [18, 26, 51]
c.895A>G	p.Lys299Glu (p.K299E)	chr1:145416550	NA	NA	NA	NA	NA	Benign	Benign	Neutral	1	France	Caucasian	26	Included: Compound heterozygote: 1 [26]
c.904G>A	p.Glu302Lys (p.E302K)	chr1:145416559	rs143496559	3.99×10^-4^	3.08×10^-4^	2.64×10^-4^	2.72×10^-4^	Probably damaging	Possibly damaging	Deleterious	4	Brazil, France	Caucasian	12,27	Included: Heterozygote: 2 [12] Excluded: Heterozygote: 2 [27]
c.929C>G	p.Ala310Gly (p.A310G)	chr1:145416584	rs7540883	2.66×10^-2^	2.52×10^-2^	6.97×10^-3^	5.43×10^-3^	Probably damaging	Possibly damaging	Neutral	22	Brazil, United States (African American)	Caucasian, African American	12,34	Included: Heterozygote: 1 [12] Excluded: Heterozygote: 21 [34]
c.934C>T	p.Gln312Ter (p.Q312*)	chr1:145416589	NA	NA	NA	NA	NA	—	—	—	3	Japan, China	East Asian	9,48,49,52	Included: Homozygote: 1 [49, 52]; Compound heterozygote: 1 [48] Excluded: Homozygote: 1 [9]
c.950G>C	p.Cys317Ser (p.C317S)	chr1:145416605	NA	NA	NA	NA	NA	Probably damaging	Probably damaging	Deleterious	1	Italy	Caucasian	53	Included: Homozygote: 1 [53]
c.959G>T	p.Gly320Val (p.G320V)	chr1:145416614	rs74315323	NA	3.08×10^-4^	1.73×10^-4^	1.58×10^-4^	Probably damaging	Probably damaging	Deleterious	45	Bangladesh/United Kingdom, Ireland, Netherland, Southeast United States, United States, Slovakia, Kosovo, Germany, Denmark, Belgium, Canada, Hungary, Australia, Croatia, Brazil, Romania, Greece, Serbia, Italy, France, India	Caucasian	5,12,16,18-20,22,24,26-28,34,36,37,39,40,46,54-65	Included: Homozygote: 30 [5, 12, 16, 18–20, 22, 24, 26–28, 34, 36, 37, 39, 40, 46, 54–65]; Compound heterozygote: 7 [16, 34, 36, 37, 39, 40] Excluded: Compound heterozygote: 4 [5, 22, 24, 26]; Homozygote: 3 [5]; Heterozygote: 1 [27]
c.960dupG	p.Cys321ValfsTer21 (p.C321fs)	chr1:145416615_145416616insG	NA	NA	NA	NA	NA	—	—	—	2	Italy	Caucasian	7,18	Included: Compound heterozygote: 1 [18] Excluded: Heterozygote: 1 [7]
c.963C>G	p.Cys321Trp (p.C321W)	chr1:145416618	rs121434374	NA	NA	1.65×10^-5^	1.22×10^-5^	Probably damaging	Probably damaging	Deleterious	1	United States	Caucasian	34	Included: Compound heterozygote: 1 [34]
c.962_963delGCinsAA	p.Cys321Ter (p.C321*)	chr1:145416617_145416618delinsAA	NA	NA	NA	8.24×10^-6^	NA	—	—	—	6	China	East Asian	9-11,29	Included: Homozygote: 5 [9, 10, 29]; Heterozygote: 1 [11]
c.976C>T	p.Arg326Ter (p.R326*)	chr1:145416631	rs74315324	NA	7.70×10^-5^	1.65×10^-5^	1.22×10^-5^	—	—	—	2	Australia, Greece	Caucasian	5,28	Included: Compound heterozygote: 1 [28] Excluded: Compound heterozygote: 1 [5]
c.982_985delTCTC	p.Ser328AspfsTer10 (p.S328fs)	chr1: 145416637_145416640delTCTC	rs786205063	NA	NA	NA	NA	—	—	—	1	Slovakia	Caucasian	40	Included: Compound heterozygote: 1 [40]
c.1004G>A	p.Arg335Gln (p.R335Q)	chr1:145416659	rs377109351	NA	1.54×10^-4^	7.41×10^-5^	5.69×10^-5^	Possibly damaging	Benign	Neutral	2	France	Caucasian	27	Excluded: Heterozygote: 2 [27]
c.1006G>T	p.Gly336Ter (p.G336*)	chr1:145416661	NA	NA	NA	NA	NA	—	—	—	4	India	Caucasian	66	Included: Homozygote: 4 [66]
c.1026delT	p.Ala343ProfsTer24 (p.A343fs)	chr1:145416681delT	NA	NA	NA	NA	NA	—	—	—	1	Sri Lanka	Caucasian	37	Included: Homozygote: 1 [37]
c.1080delC	p.Cys361ValfsTer6 (p.C361fs)	chr1:145416735delC	NA	NA	NA	NA	NA	—	—	—	1	Greece	Caucasian	5	Included: Homozygote: 1 [5]
c.1081T>C	p.Cys361Arg (p.C361R)	chr1:145416736	NA	NA	NA	NA	NA	Probably damaging	Probably damaging	Deleterious	1	France	Caucasian	26	Excluded: Compound heterozygote: 1 [26]
c.1097T>A	p.Leu366Ter (p.L366*)	chr1:145416752	rs1205047449	NA	NA	NA	NA	—	—	—	1	Canada	Caucasian	20	Included: Homozygote: 1 [20]
c.1114A>G	p.Asn372Asp (p.N372D)	chr1:145416769	rs782156457	NA	NA	8.24×10^-6^	1.22×10^-5^	Possibly damaging	Benign	Deleterious	1	France	Caucasian	27	Excluded: Heterozygote: 1 [27]
c.1151C>T	p.Ala384Val (p.A384V)	chr1:145416806	NA	NA	NA	NA	NA	Possibly damaging	Possibly damaging	Neutral	1	France	Caucasian	26	Included: Compound heterozygote: 1 [26]
c.1153C>T	p.Arg385Ter (p.R385*)	chr1:145416808	rs782803011	NA	NA	8.24×10^-6^	2.03×10^-5^	—	—	—	3	Italy, North Africa	Caucasian, North African	18,26	Included: Homozygote: 3 [18, 26]
c.-89-4dupT	—	chr1:145414649	NA	NA	NA	NA	NA	—	—	—	1	France	Caucasian	19	Excluded: Heterozygote: 1 [19]
c.-36G>A	—	chr1:145414746	NA	NA	NA	NA	NA	—	—	—	2	India	Caucasian	66	Included: Compound heterozygote: 2 [66]
c.-1624G>A	—	chr1:145413158	NA	NA	NA	NA	NA	—	—	—	2	India	Caucasian	66	Included: Compound heterozygote: 2 [66]
Gene deletion	—	—	—	NA	NA	NA	NA	—	—	—	1	Canada	Caucasian	20	Included: Heterozygote: 1 [20]

*Abbreviations*: *1000G* 1000 genome project, *ESP* exome sequencing project, *ExAC* Exome Aggregation Consortium, *GnomAD* Genome Aggregation Database, *HJV*-HH *HJV*-related hereditary hemochromatosis, *NA* not available

### Genotype–phenotype correlation in *HJV*-variant homozygotes

No significant differences in clinical features were observed between cases with compound heterozygous mutation versus homozygous mutation. Next, 73 probands with homozygous mutation status were included in an exploration of genotype–phenotype correlation. Among them, 22 and 50 of the probands had pathogenic mutations in exon 3 and exon 4, respectively, and only one had exon 2 mutations (Table 5). Moreover, there were 51 cases with missense mutations, 14 cases with nonsense mutations, 7 cases with frame-shift mutations, and 1 case caused by deletion (Table 6).

**Table 5:** Correlations of mutation locations with phenotypes in *HJV*-HH homozygous cases

	Exon 2-3	Exon 4	*P*
N	23	50	
Male, n (%)	14 (60.87%)	29 (58.00%)	8.17 × 10^-1^_C_
Ethnicities Caucasian/East Asian/African, n (%)	20/3/0 (86.96/13.04/0.00)	44/5/1 (88.00/10.00/2.00)	7.04 × 10^-1^_F_^a^
Age at diagnosis (year)	23.00 (20.00, 26.00)	28.00 (24.00, 37.00)	**6.96 × 10** ^**-3**^_**W**_
Age at presentation (year)	22.50 (20.00, 25.50)	25.00 (20.00, 32.00)	1.71 × 10^-1^_W_
Disease onset before 30 years, n (%)	21 (91.30%)	33 (66.00%)	**2.40 × 10** ^**-2**^_**C**_
Serum parameters at presentation			
Serum ferritin (ng/ml)	3065.00 (2000.00, 4485.00)	4018.50 (2500.00, 6008.50)	1.23 × 10^-1^_W_
Transferrin saturation (%)	95.00 (90.00, 100.00)	96.00 (91.00, 100.00)	5.62 × 10^-1^_W_
Complications			
Cardiomyopathy, n (%)	7 (30.43%)	21 (42.00%)	3.45 × 10^-1^_C_
Skin hyperpigmentation, n (%)	5 (21.74%)	25 (50.00%)	**3.92 × 10** ^**-2**^_**F**_
Arthropathy, n (%)	4 (17.39%)	14 (28.00%)	3.94 × 10^-1^_F_
Endocrine abnormality			
Hypogonadism, n (%)	18 (78.26%)	31 (62.00%)	1.70 × 10^-1^_C_
Glucose intolerance, n (%)	6 (26.09%)	16 (32.00%)	6.09 × 10^-1^_C_
Osteopathy, n (%)	1 (4.35%)	4 (8.00%)	1.00 × 10^-1^_F_
Thyroid abnormality, n (%)	0 (0.00%)	3 (6.00%)	5.47 × 10^-1^_F_
Liver disease			
Abnormal liver function test, n (%)	8 (34.78%)	19 (38.00%)	7.91 × 10^-1^_C_
Liver iron deposition, n (%)	15 (65.22%)	35 (70.00%)	6.83 × 10^-1^_C_
Liver fibrosis, n (%)	12 (52.17%)	20 (40.00%)	3.30 × 10^-1^_C_
Liver cirrhosis, n (%)	7 (30.43%)	13 (26.00%))	6.93 × 10^-1^_C_
Liver biopsy, n (%)	17 (73.91%)	25 (50.00%)	5.48 × 10^-2^_C_
Therapy Phlebotomy/Chelating agent/Phlebotomy & Chelating agent/ND, n (%)	6/0/2/15 (26.09/0.00/8.70/65.22)	28/3/4/15 (56.00/6.00/8.00/30.00)	—

*Abbreviations*: *HJV*-HH *HJV-*related hereditary hemochromatosis, *ND* not described

Data are shown as n (%) or median (interquartile range). *P* values were calculated to assess the intergroup differences between homozygotes with exon 2-3 mutation and homozygotes with exon 4 mutation using chi-square test, Fisher’s exact test, or Wilcoxon test as appropriate. _C_, on chi-square test; _F_, on Fisher’s exact test; _W_, on Wilcoxon test. ^a^, compared the proportions of Caucasians and East Asians. *P* values <0.05 are denoted in bold and underlined

**Table 6:** Correlations of mutation types with phenotypes in *HJV*-HH homozygous cases

	Frameshift	Nonsense	Missense	Deletion	*P*_*frameshift-nonsense*_	*P*_*frameshift -missense*_	*P*_*nonsense-missense*_
N	7	14	51	1			
Male, n (%)	5 (71.43%)	11 (78.57%)	27 (52.94%)	0 (0.00%)	1.00 × 10^-0^_F_	4.42 × 10^-1^_F_	1.27 × 10^-1^_F_
Ethnicities Caucasian/East Asian/African, n (%)	6/1/0 (85.71/14.29/0.00)	9/4/1 (64.29/28.57/7.14)	48/3/0 (94.12/5.88/0.00)	1/0/0 (100.00/0.00/0.00)	6.13 × 10^-1^_F_^a^	4.11 × 10^-1^_F_^a^	**2.68 × 10** ^**-2**^_**F**_^**a**^
Age at diagnosis (year)	21.00 (17.00, 25.00)	25.00 (19.00, 45.00)	28.00 (23.00, 32.00)	20.00	*P*_*frameshift-nonsense-missense*_ = 1.81 × 10^-1^_K_		
Age at presentation (year)	21.00 (17.00, 25.00)	24.00 (19.00, 25.00)	25.00 (21.00, 31.00)	—	*P*_*frameshift-nonsense-missense*_ = 2.34 × 10^-1^_K_		
Disease onset before 30 years, n (%)	7 (100.00%)	9 (64.29%)	37 (72.55%)	1 (100.00%)	1.24 × 10^-1^_F_	1.78 × 10^-1^_F_	5.29 × 10^-1^_F_
Serum parameters at presentation							
Serum ferritin (ng/ml)	4840.00 (2800.00, 5999.00)	2736.50 (1700.00, 7000.00)	3728.0 (2500.00, 5574.50)	1955.00	*P*_*frameshift-nonsense-missense*_ = 6.93 × 10^-1^_K_		
Transferrin saturation (%)	96.50 (94.00, 100.00)	94.00 (89.50, 97.30)	96.00 (90.00, 100.00)	—	*P*_*frameshift-nonsense-missense*_ = 3.73 × 10^-1^_K_		
Complications							
Cardiomyopathy, n (%)	3 (42.86%)	6 (42.86%)	18 (35.29%)	1 (100.00%)	1.00 × 10^-0^_F_	6.96 × 10^-1^_F_	6.04 × 10^-1^_C_
Skin hyperpigmentation, n (%)	2 (28.57%)	7 (50.00%)	20 (39.22%)	1 (100.00%)	6.42 × 10^-1^_F_	6.98 × 10^-1^_F_	4.68 × 10^-1^_C_
Arthropathy, n (%)	0 (0.00%)	2 (14.29%)	15 (29.41%)	1 (100.00%)	5.33 × 10^-1^_F_	1.73 × 10^-1^_F_	3.23 × 10^-1^_F_
Endocrine abnormality							
Hypogonadism, n (%)	6 (85.71%)	5 (35.71%)	37 (72.55%)	1 (100.00%)	6.35 × 10^-2^_F_	6.64 × 10^-1^_F_	**2.43 × 10** ^**-2**^_**F**_
Glucose intolerance, n (%)	3 (42.86%)	7 (50.00%)	12 (23.53%)	0 (0.00%)	1.00 × 10^-0^_F_	3.60 × 10^-1^_F_	5.37 × 10^-2^_C_
Osteopathy, n (%)	0 (0.00%)	0 (0.00%)	4 (7.84%)	1 (100.00%)	—	1.00 × 10^-0^_F_	1.00 × 10^-0^_F_
Thyroid abnormality, n (%)	0 (0.00%)	1 (7.14%)	2 (3.92%)	0 (0.00%)	1.00 × 10^-0^_F_	1.00 × 10^-0^_F_	5.23 × 10^-1^_F_
Liver disease							
Abnormal liver function test, n (%)	3 (42.86%)	4 (28.57%)	19 (37.25%)	1 (100.00%)	6.38 × 10^-1^_F_	1.00 × 10^-0^_F_	7.54 × 10^-1^_F_
Liver iron deposition, n (%)	5 (71.43%)	11 (78.57%)	33 (64.71%)	1 (100.00%)	1.00 × 10^-0^_F_	1.00 × 10^-0^_F_	5.20 × 10^-1^_F_
Liver fibrosis, n (%)	5 (71.43%)	3 (21.43%)	24 (47.06%)	0 (0.00%)	5.55 × 10^-2^_F_	4.23 × 10^-1^_F_	1.27 × 10^-1^_F_
Liver cirrhosis, n (%)	2 (28.57%)	3 (21.43%)	14 (27.45%)	1 (100.00%)	1.00 × 10^-0^_F_	1.00 × 10^-0^_F_	7.45 × 10^-1^_F_
Liver biopsy, n (%)	6 (85.71%)	4 (28.57%)	31 (60.78%)	1 (100.00%)	**2.37 × 10** ^**-2**^_**F**_	4.03 × 10^-1^_F_	**3.93 × 10** ^**-2**^_**F**_
Therapy Phlebotomy/Chelating agent/Phlebotomy & Chelating agent/ND, n (%)	3/0/1/3 (42.86/0.00/7.14/42.86)	9/1/0/4 (64.29/7.14/0.`00/28.57)	21/2/5/23 (41.18/3.92/9.80/45.10)	1/0/0/0 (100.00/0.00/0.00/0.00)	—	—	—

*Abbreviations*: *HJV*-HH *HJV*-related hereditary hemochromatosis, *ND* not described

Data are shown as n (%) or median (interquartile range). *P* values were calculated to assess the intergroup differences among homozygotes with frame-shift mutation, nonsense mutation, and missense mutation using chi-square test, Fisher’s exact test, or Kruskal-Wallis test as appropriate. _C_, on chi-square test; _F_, on Fisher’s exact test; _K_, on Kruskal-Wallis test. ^a^, compared the proportions of Caucasians and East Asians. *P* values <0.05 are denoted in bold and underlined

Compared to those with exon 4 mutation, homozygotes with mutations in exons 2 and 3 displayed a significantly earlier age at diagnosis (median [interquartile range, IQR]: 23.00 [20.00, 26.00] vs. 28.00 [24.00, 37.00], *P*=6.96×10^-3^). Twenty-one (91.30%) cases with exon 2-3 variants were early-onset, whereas 66.00% of those with exon 4 variants were early-onset (*P*=2.40×10^-2^). The SF and TS levels were comparable, as well as the prevalence rates of most of the complications, except that the homozygotes with exon 4 variants showed a higher prevalence of skin hyperpigmentation or freckles than did those with exon 2-3 variants (*P*=3.92×10^-2^; Table 5).

On comparisons among mutation types, no significant differences were detected in age at diagnosis, age at presentation, SF, or TS. Notably, a greater proportion of probands with missense mutations presented with hypogonadism (72.55%) compared with that among those with nonsense mutations (35.71%; *P*=2.43×10^-2^). Liver biopsy was accepted by more probands with frame-shift or missense mutations accepted liver biopsy (85.71%, 60.78%, respectively) than by those with nonsense mutations (28.57%, *P*=2.37×10^-2^, 3.93×10^-2^; Table 6).

### Therapies and outcomes among biallelic *HJV* mutation cases

As shown in Additional file 3, therapy information was provided for 70 cases. Among them, 60 cases were treated with phlebotomy, 3 with chelating agents, and 7 with phlebotomy in combination with chelating agents.

The outcome data were provided for 40 cases. Among them, five cases who had previously diagnosed with cardiomyopathy expired after diagnosis of HH-*HJV* (three of cardiac failure and two of sepsis). The patients in all other cases experienced varying degrees of improvement after therapy administration, and all cases reported complete or partial iron depletion.

Fifteen cases reported improvement after treatment, and in 12 of these cases, liver function was restored to normal. Seven cases that presented with cardiomyopathy showed significant improvement in cardiac function after therapeutic phlebotomy in combine with or without iron chelation agent administration, of which six achieved completely normalized or nearly normalized heart function. One achieved complete recovery of hypogonadism after treatment with phlebotomy and deferasirox. Two cases achieved complete recovery by iron depletion, one of which had been previously treated with insulin and was able to discontinue insulin therapy. In addition, two cases experienced improvement in bone density after phlebotomy.

## Discussion

HH is one of the most common genetic disorders in the Northern European population, affecting approximately 1 in 200 people [67]. In European populations, *HFE* homozygous and compound heterozygous mutations account for 60–95% of iron overload cases [68]. Due to the high frequency of the *HFE* C282Y mutation, a simple genetic test can confirm the diagnosis in most of these patients. However, in other parts of the world, *HFE* mutation is less common. For example, the majority of Asian HH cases are due to non-*HFE* mutation [13, 37]. A previous study based on data from the publicly available next-generation sequencing (NGS) databases demonstrated that all recessive inherited non-*HFE* forms of HH were predicted to be extremely rare around the world [3]. This rarity makes the diagnosis and management of *HJV*-HH a great challenge, and indeed, this diagnosis was frequently delayed in the affected individuals. This review systematically characterized the reported genotypic and phenotypic spectra of *HJV*-HH in multiple ethnicities by analyzing the data for 132 eligible *HJV*-variant cases. To our knowledge, this is the first study to investigate the phenotype–genotype correlation of *HJV*-HH.

The *HJV* gene was first identified in 2004 in a group of JH patients from Greece, Canada, and France [5]. HJV protein is a membrane protein that is highly expressed in the liver, skeletal muscle and heart and plays a role in iron absorption and release from cells in addition to having anti-inflammatory properties [4]. As a member of the repulsive guidance molecule (RGM) protein family, the longest isoform of the HJV protein consists of several functional motifs with an N-terminal signal peptide (amino acids [aa] 1–40), a conserved RGD motif (aa 98–100), a partial von Willebrand type D (vWD) domain (aa 167–310), and a C-terminal glycosylphosphatidylinositol (GPI) anchor domain (aa 403–426) [4]. It is highly conserved between species, except for the signal peptide and GPI anchor domain [5]. To date, 75 *HJV* variants of multiple mutation types have been identified in *HJV*-related iron-overload cases, including missense, nonsense, frame-shift, and in-frame mutations in exons 2, 3 and 4, as well as insertion in the splicing site and substitutions in the 5′ UTR. Most of the mutations are localized in evolutionally conserved residues [5]. Interestingly, multiple alterations at the positions of cysteine 80 [28, 36, 37], glycine 99 [5, 18, 37], phenylalanine 103 [7, 9], cysteine 321 [7, 9–11, 18, 29, 34], and cysteine 361 [5, 26] have been identified, which further strengthens the evidence that these regions have essential biological functions. In addition to the deleterious variants, loss-of-heterozygosity (LOH) resulting from gene deletion and uniparental disomy also has been detected in *HJV*-HH cases [20, 32], which emphasizes the involvement of novel mechanisms in the pathogenesis of HH. Thus, the multiplex ligation-dependent probe amplification method for detecting LOH should be considered in combination with sequencing of targeted gene regions when appropriate.

By associating the variants with the family origins, we observed that approximately two-thirds of the variations have been reported only in single cases or families, which was in accordance with the sporadic nature of the disease. Notably, one-third of them were recurrent mutations, most of which were detected only in the geographic origin of the respective family. Strong ethnic differences were identified in the genotype profile. G320V [5, 12, 16, 18–20, 22, 26–28, 34, 36, 37, 39, 40, 46, 54–65] and L101P [18, 26, 27, 36], the most commonly reported substitutions, were restricted to Caucasian ancestry. Among those reported in Caucasians more than three times, D149fs, R176C, and G336* were observed in Italian, French, or Indian cases only [8, 18, 19, 22, 26, 45, 66], suggesting that cases originating from different nations had their own recurrent mutations. [Q6H; C321*] *in cis* was the predominant genotype in Chinese *HJV*-HH [9–11, 29]. C321* was predicted to produce a truncated HJV protein that fails to present on the cell surface [69], whereas Q6H, which is localized in the signal peptide region, caused no or only slight alteration in HJV localization and was identified in cases with C321* only. Similarly, the other frequently detected variant in the signal peptide region, E3D, was only identified in those with *SUGP2* R639Q mutation, a deleterious mutation that affects the BMP/SMAD pathway [9]. Both Q6H and E3D were predicted to be benign and were suggested as indicators of causative variants of HH [9]. D249H and Q312* were the most common mutations in Japanese cases [43, 48–50, 52]. Q312* was also detected in Chinese cases [9], suggesting its potential as a hyper mutant spot shared by the East Asian populations. Moreover, only three variations were reported across different races, including I281T [5, 9, 10], A310G [12, 34], and R385* [18, 26]. To some extent, these findings imply the existence of important hyper mutant regions across different ethnicities.

The clinical spectrum of *HJV*-related HH varied with respect to ethnicity. First, our results confirmed that males and females were equally affected in Caucasian probands with biallelic mutation [14]. However, interestingly, males accounted for 11 of the 13 probands with biallelic mutation of East Asian ancestry, indicating the first time that East Asian males are more vulnerable than females. The phenomenon could be explained by iron loss through menstruation after puberty, which is believed to result in a less severe phenotype in females. In Caucasians, this natural advantage among females could be partially diminished because of the relatively high consumption of red meat in the Western diet, which might interact with the risk of hemochromatosis. However, considering the limited sample size of East Asians in this review, whether the findings can reflect the true distribution among East Asians requires further investigation.

Second, ethnic discrepancy in the clinical complications of *HJV*-HH was identified. Hypogonadism and arthropathy were more prevalent in Caucasian probands than in East Asian probands, despite the comparable age at diagnosis and SF and TS levels. In contrast, the prevalence rates of glucose intolerance and cardiac and hepatic complications appeared relatively higher in East Asians, but the differences were not statistically significant. The underlying mechanisms remain unclear. However, we suspect that ethnic differences in the susceptibility of target tissues might contribute to the potential differences. Considering diabetes as an example, β-cell function is considered more vulnerable in East Asians than in Caucasians [70]. In this study, in addition to a higher rate of diabetes, we found that insulin was used to treat diabetes only in East Asian cases [10, 43, 49], whereas oral anti-diabetic drugs were used in the two Caucasian cases with diabetes [16, 21, 34]. In addition, better awareness of hemochromatosis among Caucasians than among other ethnicities and populations with different cultural backgrounds could confound the above results.

Biallelic *HJV* mutations are known as the major cause of JH, but not all of the *HJV* biallelic mutation cases developed JH. In this review, over three-fourths of the probands were early-onset cases. Hypogonadism was more prevalent in early-onset *HJV*-HH, whereas liver iron deposition occurred with a higher prevalence in the late-onset cases, suggesting phenotypic variation and potential differences in pathogenesis between the two groups. In the genotype–phenotype correlation study, although we were unable to explore and compare the phenotypes of single variants due to the sporadic nature and complexity of disease, we were able to identify that more than 90% of the homozygotes with mutation in exons 2-3 developed hemochromatosis before age 30, compared with only 66% among homozygotes with exon 4 mutation, indicating that the genetic defects in exons 2-3 may have a greater deleterious impact to HJV function. Although the underlying biological mechanisms have yet to be elucidated, our findings emphasize the existence of genotype–phenotype correlation.

Experimental studies indicated that loss of HJV membrane export is central to the pathogenesis of iron overload [69, 71]. Currently reported nonsense mutations, such as Q312*, C321*, R326*, G336*, L366*, and R385*, were suspected to induce a truncated HJV protein lacking the GPI anchor motif, resulting in defective targeting of HJV to the plasma membrane. For example, the R326* mutant is mainly retained in the endoplasmic reticulum *in vitro* [69]. Frame-shift mutations that alter the reading frame of the protein are generally considered deleterious, because this leads to completely altered translation. The missense mutations can have diverse effects in the pathogenesis of iron overload. *In vivo* studies demonstrated that some, including G320V, W191C, and F170S [69], as well as mutant at TMPRSS6 cleavage site arginine 176 and 288, can cause a significant reduction of membrane-anchored HJV, which then impairs the activation of the signaling pathway involved in hepcidin up-regulation. Moreover, autoproteolysis does not occur for the arginine 176 and 288 mutant, resulting in the loss of its ability as a BMP co-receptor in the regulation of hepcidin [71]. Researchers also hypothesized that G99V and C119F cause a defect in the binding to BMP or to co-receptors, which generates an inadequate activation signal for hepcidin production [69]. L101P, located near the RGD region, is speculated to result in an alteration of RGD function [5]. Ideally, stratifying the mutations by their biological function in the regulation of hepcidin may be a better way to investigate the phenotype–genotype correlation, because of the tremendous differences in the pathogenic mechanisms. However, in the present study, we found a tendency toward a relatively earliest onset age for the frame-shift mutation homozygotes compared to that of the nonsense or missense mutation homozygotes. Interestingly, the nonsense mutation homozygotes showed the lowest prevalence of hypogonadism, as well as the lowest rate of accepting liver biopsy, compared to the missense or frameshift groups, indicating the nonsense mutations could be less harmful to the target tissues. Overall, further comparisons of biological mechanisms among the different types of mutations are needed.

Therapeutic phlebotomy and/or chelating agents were commonly used for treating hemochromatosis. Previous studies suggested that it may take longer for excess iron to removed and target levels of iron parameters to be reached following phlebotomy in JH. If not appropriately treated in the early stages of symptomatic presentation, cardiac failure becomes a leading cause of death in these patients [6]. In this review, seven *HJV*-HH cases showed significant improvement in cardiac function after therapy administration, most of which even achieved normalized or nearly normalize heart function [6], suggesting that the currently available therapies, if adequately applied, can provide a great chance of recovery from cardiomyopathy in *HJV*-HH. Notably, while some patients died from complications of cardiomyopathy [5, 33, 36, 56], two cases with cardiomyopathy died of sepsis [18, 49, 51]. This emphasizes the need for heightened vigilance not only for cardiac dysfunction but also for sepsis among *HJV*-HH patients, especially those who have already presented myocardial involvement.

The results further suggested that the reversal of endocrine dysfunction is a greater challenge compared to the improvement of hepatic function in *HJV*-HH. In hemochromatosis, hypogonadism is mainly secondary to the selective deposition of iron in the gonadotropin-producing cells of the pituitary gland, leading to impaired hormone secretion [1]. It was previously reported that gonadotropic cell dysfunction induced by the excessive iron overload of HH could be reversed if appropriate treatment is initiated early enough [72]. Sex hormone replacement therapy offers rapid correction of sexual problems and is commonly used in these patients, but iron depletion is the key to normalization of this disorder. In this review, hypogonadism was resolved in one of the juvenile-onset cases by applying phlebotomy in combination with deferasirox [12, 60]. Previous cohort studies suggested a favorable effect could be achieved for glucose control in hemochromatosis cases [10, 12, 16, 21, 43, 49, 60]. Diabetes in hemochromatosis involves two major mechanisms, beta-cell damage and insulin resistance, due to hepatic damage. In our study, two *HJV*-HH cases achieved diabetes recovery with iron depletion [10, 12, 60]. One had abnormal liver function test results but did not have cirrhosis, while the other had hepatic cirrhosis and was previously treated with insulin. After therapeutic phlebotomy, both patients achieved euglycemia and improved liver function. Osteoporosis in hemochromatosis might attributed to iron overload and its-related hypogonadism, liver failure, or parathyroid defects [1]. In the present review, most cases with osteoporosis or osteopenia also had hypogonadism, while half the cases also had liver cirrhosis. Two cases achieved partial improvement in bone density after phlebotomy [16]. Based on these results, although complete resolution of endocrine disorders was possible in *HJV*-HH, it was seldom achieved. Efficient strategies to prevent, early diagnose and reverse endocrine dysfunction in *HJV*-HH are urgently needed. However, the relationships of age, sex, genotype, and clinical features with disease outcomes are difficult to further clarify due to the complexities of the clinical manifestations and the limitation of a review study design; these relationships are of clinical significance though and need to be investigated in the future.

Finally, although the classic mode of *HJV*-HH inheritance follows an autosomal recessive pattern, the heterozygous status of *HJV* was recently reported to cause iron-overload phenotypes or even middle-age onset hemochromatosis in some cases [7–12], supporting the additive effect of the *HJV* gene in the determination of phenotype. A similar hereditary pattern was previously observed in *HFE*-HH. Studies indicated that individuals who were heterozygous for *HFE* C282Y or H63D showed elevated SF and TS levels, but did not develop complications of iron overload [73, 74]. In this review, six cases with one *HJV* mutation developed complications [7, 9, 11]. Ten *HJV* heterozygous cases also had heterozygous mutations in another HH-related gene, which implies a digenic inheritance mode [8, 9, 12]. In addition, the possibility of the existence of an unknown causal gene cannot be excluded. More heterozygous probands and a higher prevalence of complications were reported in East Asians, suggesting that East Asians might be more vulnerable to the monoallelic gene deficiency than Caucasians. Thus, these findings emphasize the importance of screening and the management of asymptomatic heterozygotes in *HJV*-HH families to avoid unwanted outcomes.

In conclusion, the current study provides a systematic review of the genotypic and phenotypic spectra of *HJV*-HH worldwide. Although the sporadic nature of *HJV*-HH determined that sequencing of the targeted genes related to HH will be the best approach to pursue a definitive diagnosis for quite a long time, knowledge of the cross-ethnicity and ethnicity-specific mutations will be beneficial for the interpretation of sequencing data as well as the establishment of ethnicity-specific approaches to genetic testing. Heterozygous relatives of *HJV*-HH patients should also be accessed for iron-overload disease. Given the large genetic and phenotypic diversity of this disease, the accumulation of data from future clinical cases is of great significance to provide better guidance for the diagnosis, prognosis and management of *HJV*-related disease, which should take into consideration patients’ ethnicity, geographical region, and genetic predisposition.

## Additional files

Additional file 1: Search terms used for database searches. (DOCX 18 kb)

Additional file 2: Included and excluded articles. (DOCX 161 kb)

Additional file 3: Clinical findings for cases with biallelic mutations. (DOCX 96 kb)

Additional file 4: Clinical findings for cases with monoallelic mutation. (DOCX 56 kb)

## Data Availability

All data generated or analyzed during this study are included in this published article and its supplementary information files.

## Abbreviations

1000G: 1000 genome project
ESP: Exome sequencing project
ExAC: Exome aggregation consortium
GnomAD: Genome aggregation database
HGVS: Human genome variation society
*HJV*-HH: *HJV*-related hereditary hemochromatosis
NA: Not available
ND: Not described
TM: Transmembrane domain
vWF Type D: von Willebrand factor type D domain
