# Supplementary material for: Genotypic and phenotypic spectra of hemojuvelin mutations in primary hemochromatosis patients: a systematic review
Source: Orphanet J Rare Dis. 2019 Jul 8;14:171. doi: 10.1186/s13023-019-1097-2 (PMC6615163; doi:10.1186/s13023-019-1097-2)
Supplement: Supplementary file 1 — Search terms used for database searches. (DOCX 18 kb) [file 13023_2019_1097_MOESM1_ESM.docx]

**Additional file 1.** Search terms used for database searches

The following search term was applied in PubMed.

((("Hemochromatosis, type 2"[Supplementary Concept] OR "Hemochromatosis, type 2"[All Fields]) OR ("Hemochromatosis, type 2"[Supplementary Concept] OR "Hemochromatosis, type 2"[All Fields] OR "juvenile hemochromatosis"[All Fields])) OR ("Hemochromatosis, type 2"[Supplementary Concept] OR "Hemochromatosis, type 2"[All Fields] OR "juvenile hemochromatosis"[All Fields])) OR ((HJV[All Fields] OR hemojuvelin[All Fields]) OR HFE2[All Fields])
