# Supplementary material for: Genotypic and phenotypic spectra of hemojuvelin mutations in primary hemochromatosis patients: a systematic review
Source: Orphanet J Rare Dis. 2019 Jul 8;14:171. doi: 10.1186/s13023-019-1097-2 (PMC6615163; doi:10.1186/s13023-019-1097-2)
Supplement: Supplementary file 2 — Included and excluded articles. (DOCX 161 kb) [file 13023_2019_1097_MOESM2_ESM.docx]

**Additional file 2.** Included and excluded articles

1. **Included articles n=57**

| **ID** | **Author** | **Title** | **Journal** | **Year** |
| --- | --- | --- | --- | --- |
| 1 | Burri, E; Decker, M; Eriksson, U; Buser, P; Hunziker, L | [28-year old patient with successfully treated dilatative cardiomyopathy] | Internist (Berl) | 2008 |
| 2 | Eisold, M; Gehrke, S; Stremmel, W; Gugler, R | [A young diabetic with small-nodule liver cirrhosis, high transferrin saturation and negative HFE test] | Dtsch Med Wochenschr | 2005 |
| 3 | Berg, L B; Milman, N T; Friis-Hansen, L; Jensen, P D; Frund, T | [Juvenile haemochromatosis caused by a homozygous Gly320Val mutation in the haemojuvelin gene] | Ugeskr Laeger | 2013 |
| 4 | Yuanfeng, L; Hongxing, Z; Haitao, Z; Xiaobo, P; Lili, B; Fuchu, H; Zewu, Q; Gangqiao, Z | [Mutation analysis of the pathogenic gene in a Chinese family with hereditary hemochromatosis] | Yi Chuan | 2014 |
| 5 | Liu, G; Niu, S; Dong, A; Cai, H; Anderson, G J; Han, B; Nie, G | A Chinese family carrying novel mutations in SEC23B and HFE2, the genes responsible for congenital dyserythropoietic anaemia II (CDA II) and primary iron overload, respectively | Br J Haematol | 2012 |
| 6 | Lee, P; Promrat, K; Mallette, C; Flynn, M; Beutler, E | A juvenile hemochromatosis patient homozygous for a novel deletion of cDNA nucleotide 81 of hemojuvelin | Acta Haematol | 2006 |
| 7 | Cherfane, C; Lee, P; Guerin, L; Brown, K | A late presentation of a fatal disease: juvenile hemochromatosis | Case Rep Med | 2013 |
| 8 | Nagayoshi, Y; Nakayama, M; Suzuki, S; Hokamaki, J; Shimomura, H; Tsujita, K; Fukuda, M; Yamashita, T; Nakamura, Y; Sugiyama, S; Ogawa, H | A Q312X mutation in the hemojuvelin gene is associated with cardiomyopathy due to juvenile haemochromatosis | Eur J Heart Fail | 2008 |
| 9 | Ravasi, G; Pelucchi, S; Mariani, R; Silvestri, L; Camaschella, C; Piperno, A | A severe hemojuvelin mutation leading to late onset of HFE2-hemochromatosis | Dig Liver Dis | 2018 |
| 10 | Kaneko, Y; Miyajima, H; Piperno, A; Tomosugi, N; Hayashi, H; Morotomi, N; Tsuchida, K; Ikeda, T; Ishikawa, A; Ota, Y; Wakusawa, S; Yoshioka, K; Kono, S; Pelucchi, S; Hattori, A; Tatsumi, Y; Okada, T; Yamagishi, M | Measurement of serum hepcidin-25 levels as a potential test for diagnosing hemochromatosis and related disorders | J Gastroenterol | 2010 |
| 11 | Pauwels, R; Vandecasteele, E; Devos, D; Pauwels, W; De Pauw, M | An unexpected cause of liver cirrhosis and cardiomyopathy in a young man | Acta Clin Belg | 2017 |
| 12 | Lanktree, M B; Sadikovic, B; Waye, J S; Levstik, A; Lanktree, B B; Yudin, J; Crowther, M A; Pare, G; Adams, P C | Clinical evaluation of a hemochromatosis next-generation sequencing gene panel | Eur J Haematol | 2017 |
| 13 | Hattori, A; Miyajima, H; Tomosugi, N; Tatsumi, Y; Hayashi, H; Wakusawa, S | Clinicopathological study of Japanese patients with genetic iron overload syndromes | Pathol Int | 2012 |
| 14 | Murugan, R C; Lee, P L; Kalavar, M R; Barton, J C | Early age-of-onset iron overload and homozygosity for the novel hemojuvelin mutation HJV R54X (exon 3; c.160A-->T) in an African American male of West Indies descent | Clin Genet | 2008 |
| 15 | van Dijk, B A; Kemna, E H; Tjalsma, H; Klaver, S M; Wiegerinck, E T; Goossens, J P; Slee, P H; Breuning, M H; Swinkels, D W | Effect of the new HJV-L165X mutation on penetrance of HFE | Blood | 2007 |
| 16 | Farrell, C P; Parker, C J; Phillips, J D | Exome sequencing for molecular characterization of non-HFE hereditary hemochromatosis | Blood Cells Mol Dis | 2015 |
| 17 | Lee, P L; Beutler, E; Rao, S V; Barton, J C | Genetic abnormalities and juvenile hemochromatosis: mutations of the HJV gene encoding hemojuvelin | Blood | 2004 |
| 18 | Cunat, S; Giansily-Blaizot, M; Bismuth, M; Blanc, F; Dereure, O; Larrey, D; Quellec, A L; Pouderoux, P; Rose, C; Raingeard, I; Renard, E; Schved, J F; Aguilar-Martinez, P | Global sequencing approach for characterizing the molecular background of hereditary iron disorders | Clin Chem | 2007 |
| 19 | Varkonyi, J; Lueff, S; Szucs, N; Pozsonyi, Z; Toth, A; Karadi, I; Pietrangelo, A | Hemochromatosis and hemojuvelin G320V homozygosity in a Hungarian woman | Acta Haematol | 2010 |
| 20 | Lee, P L; Barton, J C; Brandhagen, D; Beutler, E | Hemojuvelin (HJV) mutations in persons of European, African-American and Asian ancestry with adult onset haemochromatosis | Br J Haematol | 2004 |
| 21 | Wallace, D F; Dixon, J L; Ramm, G A; Anderson, G J; Powell, L W; Subramaniam, N | Hemojuvelin (HJV)-associated hemochromatosis: analysis of HJV and HFE mutations and iron overload in three families | Haematologica | 2005 |
| 22 | Neroldova, M; Frankova, S; Stranecky, V; Honsova, E; Luksan, O; Benes, M; Michalova, K; Kmoch, S; Jirsa, M | Hereditary haemochromatosis caused by homozygous HJV mutation evolved through paternal disomy | Clin Genet | 2015 |
| 23 | Santos, P C; Cancado, R D; Pereira, A C; Schettert, I T; Soares, R A; Pagliusi, R A; Hirata, R D; Hirata, M H; Teixeira, A C; Figueiredo, M S; Chiattone, C S; Krieger, J E; Guerra-Shinohara, E M | Hereditary hemochromatosis: mutations in genes involved in iron homeostasis in Brazilian patients | Blood Cells Mol Dis | 2011 |
| 24 | Gehrke, S G; Pietrangelo, A; Kascak, M; Braner, A; Eisold, M; Kulaksiz, H; Herrmann, T; Hebling, U; Bents, K; Gugler, R; Stremmel, W | HJV gene mutations in European patients with juvenile hemochromatosis | Clin Genet | 2005 |
| 25 | Santos, P C; Cancado, R D; Pereira, A C; Chiattone, C S; Krieger, J E; Guerra-Shinohara, E M | HJV hemochromatosis, iron overload, and hypogonadism in a Brazilian man: treatment with phlebotomy and deferasirox | Acta Haematol | 2010 |
| 26 | Janosi, A; Andrikovics, H; Vas, K; Bors, A; Hubay, M; Sapi, Z; Tordai, A | Homozygosity for a novel nonsense mutation (G66X) of the HJV gene causes severe juvenile hemochromatosis with fatal cardiomyopathy | Blood | 2005 |
| 27 | Militaru, M S; Popp, R A; Trifa, A P | Homozygous G320V mutation in the HJV gene causing juvenile hereditary haemochromatosis type A. A case report | J Gastrointestin Liver Dis | 2010 |
| 28 | Huang, F W; Rubio-Aliaga, I; Kushner, J P; Andrews, N C; Fleming, M D | Identification of a novel mutation (C321X) in HJV | Blood | 2004 |
| 29 | Biasiotto, G; Roetto, A; Daraio, F; Polotti, A; Gerardi, G M; Girelli, D; Cremonesi, L; Arosio, P; Camaschella, C | Identification of new mutations of hepcidin and hemojuvelin in patients with HFE C282Y allele | Blood Cells Mol Dis | 2004 |
| 30 | Wang, Y; "Du Y"; Liu, G; Guo, S; Hou, B; Jiang, X; Han, B; Chang, Y; Nie, G | Identification of novel mutations in HFE, HFE2, TfR2, and SLC40A1 genes in Chinese patients affected by hereditary hemochromatosis | Int J Hematol | 2017 |
| 31 | Lok, C Y; Merryweather-Clarke, A T; Viprakasit, V; Chinthammitr, Y; Srichairatanakool, S; Limwongse, C; Oleesky, D; Robins, A J; Hudson, J; Wai, P; Premawardhena, A; de Silva, H J; Dassanayake, A; McKeown, C; Jackson, M; Gama, R; Khan, N; Newman, W; Banait, G; Chilton, A; Wilson-Morkeh, I; Weatherall, D J; Robson, K J | Iron overload in the Asian community | Blood | 2009 |
| 32 | Ikuta, K; Hatayama, M; Addo, L; Toki, Y; Sasaki, K; Tatsumi, Y; Hattori, A; Kato, A; Kato, K; Hayashi, H; Suzuki, T; Kobune, M; Tsutsui, M; Gotoh, A; Aota, Y; Matsuura, M; Hamada, Y; Tokuda, T; Komatsu, N; Kohgo, Y | Iron overload patients with unknown etiology from national survey in Japan | Int J Hematol | 2017 |
| 33 | Ramzan, K; Imtiaz, F; Al-Ashgar, H I; AlSayed, M; Sulaiman, R A | Juvenile hemochromatosis and hepatocellular carcinoma in a patient with a novel mutation in the HJV gene | Eur J Med Genet | 2017 |
| 34 | Pelusi, S; Rametta, R; Della, Corte C; Congia, R; Dongiovanni, P; Pulixi, E A; Fargion, S; Fracanzani, A L; Nobili, V; Valenti, L | Juvenile hemochromatosis associated with heterozygosity for novel hemojuvelin mutations and with unknown cofactors | Ann Hepatol | 2014 |
| 35 | Aguilar-Martinez, P; Lok, C Y; Cunat, S; Cadet, E; Robson, K; Rochette, J | Juvenile hemochromatosis caused by a novel combination of hemojuvelin G320V/R176C mutations in a 5-year old girl | Haematologica | 2007 |
| 36 | Daraio, F; Ryan, E; Gleeson, F; Roetto, A; Crowe, J; Camaschella, C | Juvenile hemochromatosis due to G320V/Q116X compound heterozygosity of hemojuvelin in an Irish patient | Blood Cells Mol Dis | 2005 |
| 37 | Brakensiek, K; Fegbeutel, C; Malzer, M; Struber, M; Kreipe, H; Stuhrmann, M | Juvenile hemochromatosis due to homozygosity for the G320V mutation in the HJV gene with fatal outcome | Clin Genet | 2009 |
| 38 | Filali, M; Le Jeunne, C; Durand, E; Grinda, J M; Roetto, A; Daraio, F; Bruneval, P; Jeunemaitre, X; Gimenez-Roqueplo, A P | Juvenile hemochromatosis HJV-related revealed by cardiogenic shock | Blood Cells Mol Dis | 2004 |
| 39 | Malekzadeh, M M; Radmard, A R; Nouroozi, A; Akbari, M R; Amini, M; Navabakhsh, B; Caleffi, A; Pietrangelo, A; Malekzadeh, R | Juvenile Hemochromatosis, Genetic Study and Long-term Follow up after Therapy | Middle East J Dig Dis | 2014 |
| 40 | Altes, A; Bach, V; Ruiz, A; Esteve, A; Felez, J; Remacha, A F; Sarda, M P; Baiget, M | Mutations in HAMP and HJV genes and their impact on expression of clinical hemochromatosis in a cohort of 100 Spanish patients homozygous for the C282Y mutation of HFE gene | Ann Hematol | 2009 |
| 41 | Papanikolaou, G; Samuels, M E; Ludwig, E H; MacDonald, M L; Franchini, P L; Dube, M P; Andres, L; MacFarlane, J; Sakellaropoulos, N; Politou, M; Nemeth, E; Thompson, J; Risler, J K; Zaborowska, C; Babakaiff, R; Radomski, C C; Pape, T D; Davidas, O; Christakis, J; Brissot, P; Lockitch, G; Ganz, T; Hayden, M R; Goldberg, Y P | Mutations in HFE2 cause iron overload in chromosome 1q-linked juvenile hemochromatosis | Nat Genet | 2004 |
| 42 | de Diego, C; Opazo, S; Sanchez-Castano, A; Martinez-Castro, P | New HJV mutation in a patient with hyperferritinemia and H63D homozygosity for the HFE gene | Int J Hematol | 2007 |
| 43 | Ka, C; Le Gac, G; Letocart, E; Gourlaouen, I; Martin, B; Ferec, C | Phenotypic and functional data confirm causality of the recently identified hemojuvelin p.r176c missense mutation | Haematologica | 2007 |
| 44 | Cooray, S D; Heerasing, N M; Selkrig, L A; Subramaniam, V N; Hamblin, P S; McDonald, C J; McLean, C A; McNamara, E; Leet, A S; Roberts, S K | Reversal of end-stage heart failure in juvenile hemochromatosis with iron chelation therapy: a case report | J Med Case Rep | 2018 |
| 45 | Lanzara, C; Roetto, A; Daraio, F; Rivard, S; Ficarella, R; Simard, H; Cox, T M; Cazzola, M; Piperno, A; Gimenez-Roqueplo, A P; Grammatico, P; Volinia, S; Gasparini, P; Camaschella, C | Spectrum of hemojuvelin gene mutations in 1q-linked juvenile hemochromatosis | Blood | 2004 |
| 46 | Le Gac, G; Scotet, V; Ka, C; Gourlaouen, I; Bryckaert, L; Jacolot, S; Mura, C; Ferec, C | The recently identified type 2A juvenile haemochromatosis gene (HJV), a second candidate modifier of the C282Y homozygous phenotype | Hum Mol Genet | 2004 |
| 47 | Koyama, C; Hayashi, H; Wakusawa, S; Ueno, T; Yano, M; Katano, Y; Goto, H; Kidokoro, R | Three patients with middle-age-onset hemochromatosis caused by novel mutations in the hemojuvelin gene | J Hepatol | 2005 |
| 48 | Li, S; Xue, J; Chen, B; Wang, Q; Shi, M; Xie, X; Zhang, L | Two middle-age-onset hemochromatosis patients with heterozygous mutations in the hemojuvelin gene in a Chinese family | Int J Hematol | 2014 |
| 49 | Lv, T; Zhang, W; Xu, A; Li, Y; Zhou, D; Zhang, B; Li, X; Zhao, X; Wang, Y; Wang, X; Duan, W; Wang, Q; Xu, H; Zheng, J; Zhao, R; Zhu, L; Dong, Y; Lu, L; Chen, Y; Long, J; Zheng, S; Wang, W; You, H; Jia, J; Ou, X; Huang, J | Non-HFE mutations in haemochromatosis in China: combination of heterozygous mutations involving HJV signal peptide variants | J Med Genet | 2018 |
| 50 | Dhillon, B K; Chopra, G; Jamwal, M; Chandak, G R; Duseja, A; Malhotra, P; Chawla, Y K; Garewal, G; Das, R | Adult onset hereditary hemochromatosis is associated with a novel recurrent Hemojuvelin (HJV) gene mutation in north Indians | Blood Cells Mol Dis | 2018 |
| 51 | Smit SL, Peters TMA, Gisbertz IAM, Moolenaar W, Hendriks Y, Vincent HH, Houtsma D, Loosveld OJL, van Herwaarden AE, Rennings AJM, Swinkels DW. | Variable workup calls for guideline development for type 2A hereditary haemochromatosis. | Neth J Med | 2018 |
| 52 | Hamdi-Rozé H, Ben Ali Z, Ropert M, Detivaud L, Aggoune S, Simon D, Pelletier G, Deugnier Y, David V, Bardou-Jacquet E | Variable expressivity of HJV related hemochromatosis: "Juvenile" hemochromatosis? | Blood Cells Mol Dis | 2018 |
| 53 | Lee, P L; Beutler, E | Regulation of hepcidin and iron-overload disease | Annu Rev Pathol | 2009 |
| 54 | Maeda, T; Nakamaki, T; Saito, B; Nakashima, H; Ariizumi, H; Yanagisawa, K; Hattori, A; Tatsumi, Y; Hayashi, H; Suzuki, K; Tomoyasu, S | Hemojuvelin hemochromatosis receiving iron chelation therapy with deferasirox: improvement of liver disease activity, cardiac and hematological function | Eur J Haematol | 2012 |
| 55 | Angelopoulos, N; Papanikolaou, G; Noutsou, M; Rombopoulos, G; Goula, A; Tolis, G | Glucose metabolism, insulin secretion and insulin sensitivity in juvenile hemochromatosis. A case report and review of the literature | Exp Clin Endocrinol Diabetes | 2007 |
| 56 | Angelopoulos, N G; Goula, A K; Papanikolaou, G; Tolis, G | Osteoporosis in HFE2 juvenile hemochromatosis. A case report and review of the literature | Osteoporos Int | 2006 |
| 57 | Angelopoulos, N G; Goula, A; Dimitriou, E; Tolis, G | Reversibility of hypogonadotropic hypogonadism in a patient with the juvenile form of hemochromatosis | Fertil Steril | 2005 |

1. **Records excluded after title and abstract screened with reasons n=465**

| **ID** | **Exclusion reason** | **Author** | **Title** | **Journal** | **Year** |
| --- | --- | --- | --- | --- | --- |
| 1 | Experimental study | Xu, A; Li, Y; Chen, W; Li, X; Zhang, W; Li, S; Wu, L; Wu, Z; Zhang, B; Ou, X; Huang, J | "Haemochromatotic" characteristics of the human BEL-7402 cell line | Br J Haematol | 2017 |
| 2 | Genetic sequencing not performed | OLIVER-PASCUAL, E; SANZ, IBANEZ J; MARTINEZ, PAVON J; CASTILLO, E; ELOSEGUI, C; OLIVER, A; FERNANDEZ-BASABE, E; PURAS, E | [A curious case of juvenile hemochromatosis with cirrhotic hepatosplenomegaly, hemolytic anemia, labile diabetes with melanodermia, hypophysogonadal insufficiency and myocardial infarction] | Rev Esp Enferm Apar Dig Nutr | 1960 |
| 3 | SNP study | Piao, W; Wang, L; Zhang, T; Wang, Z; Shangguan, Shaofang; Sun, J; Huo, J | [Association of genetic polymorphisms in HjV-BMPs-SMADs pathway of hepcidin regulation with the incidence of anemia in Chinese adolescent aged 12 -18 years old] | Wei Sheng Yan Jiu | 2016 |
| 4 | Genetic sequencing not performed | PORTELLA, A; GUIDA, V; VALERIO, V | [Case of juvenile hemochromatosis with endocrine changes] | Riforma Med | 1957 |
| 5 | Not primary hemochromatosis | Wang, L; Liu, G; Zhang, Q; Cai, H; Niu, S W; Han, B; Nie, G J | [Congenital dyserythropoietic anemia type II with novel mutations in SEC23B and HFE2 genes: a Chinese family survey] | Zhonghua Xue Ye Xue Za Zhi | 2013 |
| 6 | Review | Brissot, P; Laine, F; Moirand, R; Loreal, O | [Genetics and physiopathology of hemochromatosis] | Rev Prat | 2000 |
| 7 | Review | Le Gall, J Y; Jouanolle, A M; Fergelot, P; Mosser, J; David, V | [Genetics of hereditary iron overload] | Bull Acad Natl Med | 2004 |
| 8 | Review | Brissot, P | [Haemochromatoses. New understanding, new treatments] | Gastroenterol Clin Biol | 2009 |
| 9 | Review | PALACIO, J; SANCHEZ, B; HOJMAN, D; PEREZ, ACEBO H | [Hemochromatosis. Juvenile cardiac form] | Prensa Med Argent | 1960 |
| 10 | Review | Sela, B A | [Hepcidin--the discovery of a small protein with a pivotal role in iron homeostasis] | Harefuah | 2008 |
| 11 | Review | de Korwin, J D | [Hereditary and acquired iron overload] | Nephrol Ther | 2006 |
| 12 | Review | Sikorska, K; Bielawski, K P; Romanowski, T; Stalke, P | [Hereditary hemochromatosis: the most frequent inherited human disease] | Postepy Hig Med Dosw (Online) | 2006 |
| 13 | Review | Cojocariu, C; Trifan, A; Stanciu, C | [Hereditary hemochromatosis] | Rev Med Chir Soc Med Nat Iasi | 2007 |
| 14 | Review | Le Gall, J Y; Jouanolle, A M; Mosser, J; David, V | [Human iron metabolism] | Bull Acad Natl Med | 2005 |
| 15 | Review | Smirnov, O A | [Hypersiderosis and dissiderosis in the context of data on hemochromatosis microelementosis] | Arkh Patol | 2008 |
| 16 | Review | Toxqui, L; De Piero, A; Courtois, V; Bastida, S; Sanchez-Muniz, F J; Vaquero, M P | [Iron deficiency and overload. Implications in oxidative stress and cardiovascular health] | Nutr Hosp | 2010 |
| 17 | Review | Omar, S; Feki, M; Kaabachi, N | [Iron metabolism, overview and recent insights] | Ann Biol Clin (Paris) | 2006 |
| 18 | Review | Vantyghem, M C; Girardot, C; Boulogne, A; Wemeau, J L | [Iron overload and insulin resistance] | Presse Med | 2005 |
| 19 | Genetic sequencing not performed | NUSSBAUMER, T; PLATTNER, H C; RYWLIN, A | [Juvenile hemochromatosis in three sisters and one brother associated with consanguinity of the parents; anatomo-clinical and genetic study of the endocrino-hepato-myocardial syndrome] | J Genet Hum | 1952 |
| 20 | Review | Cadet, E; Perez, A S; Capron, D; Rochette, J | [Molecular basis in hereditary haemochromatosis] | Rev Med Interne | 2005 |
| 21 | Not HJV gene-related disease | Wu, S; Yan, C; Shen, X | [Molecular genetic susceptibility to lead poisoning] | Wei Sheng Yan Jiu | 2004 |
| 22 | Review | Aguilar-Martinez, P | [Non-HFE-related hereditary iron overload] | Presse Med | 2007 |
| 23 | Review | Raszeja-Wyszomirska, J; Lawniczak, M; Milkiewicz, P | [Novel aspects of pathogenesis of hereditary hemochromatosis] | Pol Merkur Lekarski | 2008 |
| 24 | Review | Loreal, O; Ropert, M; Mosser, A; Dehais, V; Deugnier, Y; David, V; Brissot, P; Jouanolle, A M | [Pathophysiology and genetics of classic HFE (type 1) hemochromatosis] | Presse Med | 2007 |
| 25 | Review | Iwai, K; Hishikawa, K | [Primary hemochromatosis as a disease provoked by hepcidin deficiency?] | Nihon Rinsho | 2006 |
| 26 | Review | Borch-Iohnsen, B; Hagve, T A; Hauge, A; Thorstensen, K | [Regulation of the iron metabolism] | Tidsskr Nor Laegeforen | 2009 |
| 27 | Not primary hemochromatosis | Perez-Aguilar, F; Benlloch, S; Berenguer, M | [Study of patients referred for elevated ferritin levels and/or transferrin saturation: significance of non-alcoholic fatty liver disease] | Gastroenterol Hepatol | 2004 |
| 28 | Not primary hemochromatosis | Sun, G; Tan, Z; Fan, L; Wang, J; Yang, Y; Zhang, W | 1q21.1 microduplication in a patient with mental impairment and congenital heart defect | Mol Med Rep | 2015 |
| 29 | Experimental study | Verga, Falzacappa MV; Casanovas, G; Hentze, M W; Muckenthaler, M U | A bone morphogenetic protein (BMP)-responsive element in the hepcidin promoter controls HFE2-mediated hepatic hepcidin expression and its response to IL-6 in cultured cells | J Mol Med (Berl) | 2008 |
| 30 | Experimental study | Tsuchiya, H; Sakabe, T; Akechi, Y; Ikeda, R; Nishio, R; Terabayashi, K; Matsumi, Y; Hoshikawa, Y; Kurimasa, A; Shiota, G | A close association of abnormal iron metabolism with steatosis in the mice fed a choline-deficient diet | Biol Pharm Bull | 2010 |
| 31 | Not HJV gene-related disease | Milet, J; Le Gac, G; Scotet, V; Gourlaouen, I; Theze, C; Mosser, J; Bourgain, C; Deugnier, Y; Ferec, C | A common SNP near BMP2 is associated with severity of the iron burden in HFE p.C282Y homozygous patients: a follow-up study | Blood Cells Mol Dis | 2010 |
| 32 | Experimental study | Gutschow, P; Schmidt, P J; Han, H; Ostland, V; Bartnikas, T B; Pettiglio, M A; Herrera, C; Butler, J S; Nemeth, E; Ganz, T; Fleming, M D; Westerman, M | A competitive enzyme-linked immunosorbent assay specific for murine hepcidin-1: correlation with hepatic mRNA expression in established and novel models of dysregulated iron homeostasis | Haematologica | 2015 |
| 33 | Experimental study | Padda, R S; Gkouvatsos, K; Guido, M; Mui, J; Vali, H; Pantopoulos, K | A high-fat diet modulates iron metabolism but does not promote liver fibrosis in hemochromatotic Hjv(-)/(-) mice | Am J Physiol Gastrointest Liver Physiol | 2015 |
| 34 | Not HJV gene-related disease | Delatycki, M B; Allen, K J; Gow, P; MacFarlane, J; Radomski, C; Thompson, J; Hayden, M R; Goldberg, Y P; Samuels, M E | A homozygous HAMP mutation in a multiply consanguineous family with pseudo-dominant juvenile hemochromatosis | Clin Genet | 2004 |
| 35 | Experimental study | Huang, F W; Pinkus, J L; Pinkus, G S; Fleming, M D; Andrews, N C | A mouse model of juvenile hemochromatosis | J Clin Invest | 2005 |
| 36 | Not primary hemochromatosis | Melis, M A; Cau, M; Congiu, R; Sole, G; Barella, S; Cao, A; Westerman, M; Cazzola, M; Galanello, R | A mutation in the TMPRSS6 gene, encoding a transmembrane serine protease that suppresses hepcidin production, in familial iron deficiency anemia refractory to oral iron | Haematologica | 2008 |
| 37 | Not primary hemochromatosis | Pagani, A; Colucci, S; Bocciardi, R; Bertamino, M; Dufour, C; Ravazzolo, R; Silvestri, L; Camaschella, C | A new form of IRIDA due to combined heterozygous mutations of TMPRSS6 and ACVR1A encoding the BMP receptor ALK2 | Blood | 2017 |
| 38 | Not HJV gene-related disease | Pointon, J J; Lok, C Y; Shearman, J D; Suckling, R J; Rochette, J; Merryweather-Clarke, A T; Robson, K J | A novel HFE mutation (c.del478) results in nonsense-mediated decay of the mutant transcript in a hemochromatosis patient | Blood Cells Mol Dis | 2009 |
| 39 | Experimental study | Koliaraki, V; Marinou, M; Vassilakopoulos, T P; Vavourakis, E; Tsochatzis, E; Pangalis, G A; Papatheodoridis, G; Stamoulakatou, A; Swinkels, D W; Papanikolaou, G; Mamalaki, A | A novel immunological assay for hepcidin quantification in human serum | PLoS One | 2009 |
| 40 | Not primary hemochromatosis | Choi, H S; Yang, H R; Song, S H; Seo, J Y; Lee, K O; Kim, H J | A novel mutation Gly603Arg of TMPRSS6 in a Korean female with iron-refractory iron deficiency anemia | Pediatr Blood Cancer | 2012 |
| 41 | Experimental study | Moreno-Carralero, M I; Munoz-Munoz, J A; Cuadrado-Grande, N; Lopez-Rodriguez, R; Jose, Hernandez-Alfaro M; Del-Castillo-Rueda, A; Enriquez-de-Salamanca, R; Mendez, M; Moran-Jimenez, M J | A novel mutation in the SLC40A1 gene associated with reduced iron export in vitro | Am J Hematol | 2014 |
| 42 | Not primary hemochromatosis | Altamura, S; D'Alessio, F; Selle, B; Muckenthaler, M U | A novel TMPRSS6 mutation that prevents protease auto-activation causes IRIDA | Biochem J | 2010 |
| 43 | Experimental study | Chen, W; Sun, C C; Chen, S; Meynard, D; Babitt, J L; Lin, H Y | A novel validated enzyme-linked immunosorbent assay to quantify soluble hemojuvelin in mouse serum | Haematologica | 2013 |
| 44 | Experimental study | Yoshikawa, O; Ebata, Y; Tsuchiya, H; Kawahara, A; Kojima, C; Ikeda, Y; Hama, S; Kogure, K; Shudo, K; Shiota, G | A retinoic acid receptor agonist tamibarotene suppresses iron accumulation in the liver | Obesity (Silver Spring) | 2013 |
| 45 | Not HJV gene-related disease | Piao, W; Wang, L; Zhang, T; Wang, Z; Shangguan, S; Sun, J; Huo, J | A single-nucleotide polymorphism in transferrin is associated with soluble transferrin receptor in Chinese adolescents | Asia Pac J Clin Nutr | 2017 |
| 46 | Review | Chen, J; Chloupkova, M | Abnormal iron uptake and liver cancer | Cancer Biol Ther | 2009 |
| 47 | Not HJV gene-related disease | Vercesi, E; Cerani, P; Rolandi, V; Rovati, A; Bergamaschi, G | Abnormal regulation of HFE mRNA expression does not contribute to primary iron overload | Haematologica | 2000 |
| 48 | Not primary hemochromatosis | Przybylowski, P; Wasilewski, G; Golabek, K; Bachorzewska-Gajewska, H; Dobrzycki, S; Koc-Zorawska, E; Malyszko, J | Absolute and Functional Iron Deficiency Is a Common Finding in Patients With Heart Failure and After Heart Transplantation | Transplant Proc | 2016 |
| 49 | Experimental study | Sebastiani, G; Gkouvatsos, K; Maffettone, C; Busatto, G; Guido, M; Pantopoulos, K | Accelerated CCl4-induced liver fibrosis in Hjv-/- mice, associated with an oxidative burst and precocious profibrogenic gene expression | PLoS One | 2011 |
| 50 | Experimental study | Canali, S; Core, A B; Zumbrennen-Bullough, K B; Merkulova, M; Wang, C Y; Schneyer, A L; Pietrangelo, A; Babitt, J L | Activin B Induces Noncanonical SMAD1/5/8 Signaling via BMP Type I Receptors in Hepatocytes: Evidence for a Role in Hepcidin Induction by Inflammation in Male Mice | Endocrinology | 2016 |
| 51 | Experimental study | van Swelm, R P; Laarakkers, C M; Blous, L; Peters, J G; Blaney, Davidson EN; van der Kraan, P M; Swinkels, D W; Masereeuw, R; Russel, F G | Acute acetaminophen intoxication leads to hepatic iron loading by decreased hepcidin synthesis | Toxicol Sci | 2012 |
| 52 | Experimental study | Das, S K; Zhabyeyev, P; Basu, R; Patel, V B; Dyck, JRB; Kassiri, Z; Oudit, G Y | Advanced iron-overload cardiomyopathy in a genetic murine model is rescued by resveratrol therapy | Biosci Rep | 2018 |
| 53 | Review | Fleming, R E | Advances in understanding the molecular basis for the regulation of dietary iron absorption | Curr Opin Gastroenterol | 2005 |
| 54 | Experimental study | Costa, E; Fernandes, J; Ribeiro, S; Sereno, J; Garrido, P; Rocha-Pereira, P; Coimbra, S; Catarino, C; Belo, L; Bronze-da-Rocha, E; Vala, H; Alves, R; Reis, F; Santos-Silva, A | Aging is Associated with Impaired Renal Function, INF-gamma Induced Inflammation and with Alterations in Iron Regulatory Proteins Gene Expression | Aging Dis | 2014 |
| 55 | Experimental study | Bolondi, G; Garuti, C; Corradini, E; Zoller, H; Vogel, W; Finkenstedt, A; Babitt, J L; Lin, H Y; Pietrangelo, A | Altered hepatic BMP signaling pathway in human HFE hemochromatosis | Blood Cells Mol Dis | 2010 |
| 56 | Experimental study | Abbasi, M H; Fatima, S; Khawar, M B; Jahan, S; Sheikh, N | An In Vivo Study on Intoxicating Effects of Nerium oleander Water Based Extract on Multiorgans of Wistar Rat | Can J Gastroenterol Hepatol | 2018 |
| 57 | Experimental study | Schmidt, P J; Toudjarska, I; Sendamarai, A K; Racie, T; Milstein, S; Bettencourt, B R; Hettinger, J; Bumcrot, D; Fleming, M D | An RNAi therapeutic targeting Tmprss6 decreases iron overload in Hfe(-/-) mice and ameliorates anemia and iron overload in murine beta-thalassemia intermedia | Blood | 2013 |
| 58 | Not primary hemochromatosis | Castiglioni, E; Finazzi, D; Goldwurm, S; Pezzoli, G; Forni, G; Girelli, D; Maccarinelli, F; Poli, M; Ferrari, M; Cremonesi, L; Arosio, P | Analysis of nucleotide variations in genes of iron management in patients of Parkinson's disease and other movement disorders | Parkinsons Dis | 2010 |
| 59 | Not primary hemochromatosis | Camberlein, E; Zanninelli, G; Detivaud, L; Lizzi, A R; Sorrentino, F; Vacquer, S; Troadec, M B; Angelucci, E; Abgueguen, E; Loreal, O; Cianciulli, P; Lai, M E; Brissot, P | Anemia in beta-thalassemia patients targets hepatic hepcidin transcript levels independently of iron metabolism genes controlling hepcidin expression | Haematologica | 2008 |
| 60 | Review | Roy, C N; Andrews, N C | Anemia of inflammation: the hepcidin link | Curr Opin Hematol | 2005 |
| 61 | Review | Latunde-Dada, G O; McKie, A T; Simpson, R J | Animal models with enhanced erythropoiesis and iron absorption | Biochim Biophys Acta | 2006 |
| 62 | Not primary hemochromatosis | Kovac, S; Boser, P; Cui, Y; Ferring-Appel, D; Casarrubea, D; Huang, L; Fung, E; Popp, A; Mueller, B K; Hentze, M W | Anti-hemojuvelin antibody corrects anemia caused by inappropriately high hepcidin levels | Haematologica | 2016 |
| 63 | Experimental study | Boser, P; Seemann, D; Liguori, M J; Fan, L; Huang, L; Hafner, M; Popp, A; Mueller, B K | Anti-repulsive Guidance Molecule C (RGMc) Antibodies Increases Serum Iron in Rats and Cynomolgus Monkeys by Hepcidin Downregulation | AAPS J | 2015 |
| 64 | Review | Young, J | Approach to the male patient with congenital hypogonadotropic hypogonadism | J Clin Endocrinol Metab | 2012 |
| 65 | Genetic sequencing not performed | Vaiopoulos, G; Papanikolaou, G; Politou, M; Jibreel, I; Sakellaropoulos, N; Loukopoulos, D | Arthropathy in juvenile hemochromatosis | Arthritis Rheum | 2003 |
| 66 | Not primary hemochromatosis | Callon, C; Wood, E; Marsh, D; Li, K; Montaner, J; Kerr, T | Barriers and facilitators to methadone maintenance therapy use among illicit opiate injection drug users in Vancouver | J Opioid Manag | 2006 |
| 67 | Experimental study | Gibert, Y; Lattanzi, V J; Zhen, A W; Vedder, L; Brunet, F; Faasse, S A; Babitt, J L; Lin, H Y; Hammerschmidt, M; Fraenkel, P G | BMP signaling modulates hepcidin expression in zebrafish embryos independent of hemojuvelin | PLoS One | 2011 |
| 68 | Not primary hemochromatosis | Shi, Y J; Pan, X T | BMP6 and BMP4 expression in patients with cancer-related anemia and its relationship with hepcidin and s-HJV | Genet Mol Res | 2016 |
| 69 | Experimental study | Rausa, M; Pagani, A; Nai, A; Campanella, A; Gilberti, M E; Apostoli, P; Camaschella, C; Silvestri, L | Bmp6 expression in murine liver non parenchymal cells: a mechanism to control their high iron exporter activity and protect hepatocytes from iron overload? | PLoS One | 2015 |
| 70 | Experimental study | Andriopoulos, B Jr; Corradini, E; Xia, Y; Faasse, S A; Chen, S; Grgurevic, L; Knutson, M D; Pietrangelo, A; Vukicevic, S; Lin, H Y; Babitt, J L | BMP6 is a key endogenous regulator of hepcidin expression and iron metabolism | Nat Genet | 2009 |
| 71 | Experimental study | Casanovas, G; Mleczko-Sanecka, K; Altamura, S; Hentze, M W; Muckenthaler, M U | Bone morphogenetic protein (BMP)-responsive elements located in the proximal and distal hepcidin promoter are critical for its response to HJV/BMP/SMAD | J Mol Med (Berl) | 2009 |
| 72 | Experimental study | Canali, S; Wang, C Y; Zumbrennen-Bullough, K B; Bayer, A; Babitt, J L | Bone morphogenetic protein 2 controls iron homeostasis in mice independent of Bmp6 | Am J Hematol | 2017 |
| 73 | Experimental study | Babitt, J L; Huang, F W; Wrighting, D M; Xia, Y; Sidis, Y; Samad, T A; Campagna, J A; Chung, R T; Schneyer, A L; Woolf, C J; Andrews, N C; Lin, H Y | Bone morphogenetic protein signaling by hemojuvelin regulates hepcidin expression | Nat Genet | 2006 |
| 74 | Experimental study | Truksa, J; Peng, H; Lee, P; Beutler, E | Bone morphogenetic proteins 2, 4, and 9 stimulate murine hepcidin 1 expression independently of Hfe, transferrin receptor 2 (Tfr2), and IL-6 | Proc Natl Acad Sci U S A | 2006 |
| 75 | Review | Parrow, N L; Fleming, R E | Bone morphogenetic proteins as regulators of iron metabolism | Annu Rev Nutr | 2014 |
| 76 | Review | Gulati, V; Harikrishnan, P; Palaniswamy, C; Aronow, W S; Jain, D; Frishman, W H | Cardiac involvement in hemochromatosis | Cardiol Rev | 2014 |
| 77 | Not primary hemochromatosis | Roy, N B; Myerson, S; Schuh, A H; Bignell, P; Patel, R; Wainscoat, J S; McGowan, S; Marchi, E; Atoyebi, W; Littlewood, T; Chacko, J; Vyas, P; Killick, S B | Cardiac iron overload in transfusion-dependent patients with myelodysplastic syndromes | Br J Haematol | 2011 |
| 78 | Experimental study | Stirnberg, M; Maurer, E; Arenz, K; Babler, A; Jahnen-Dechent, W; Gutschow, M | Cell surface serine protease matriptase-2 suppresses fetuin-A/AHSG-mediated induction of hepcidin | Biol Chem | 2015 |
| 79 | Experimental study | Sheikh, N; Dudas, J; Ramadori, G | Changes of gene expression of iron regulatory proteins during turpentine oil-induced acute-phase response in the rat | Lab Invest | 2007 |
| 80 | Experimental study | Wang, H; An, P; Xie, E; Wu, Q; Fang, X; Gao, H; Zhang, Z; Li, Y; Wang, X; Zhang, J; Li, G; Yang, L; Liu, W; Min, J; Wang, F | Characterization of ferroptosis in murine models of hemochromatosis | Hepatology | 2017 |
| 81 | Experimental study | Farah, J; Trianni, A; Ciraj-Bjelac, O; Clairand, I; De Angelis, C; Delle, Canne S; Hadid, L; Huet, C; Jarvinen, H; Negri, A; Novak, L; Pinto, M; Siiskonen, T; Waryn, M J; Knezevic, Z | Characterization of XR-RV3 GafChromic((R)) films in standard laboratory and in clinical conditions and means to evaluate uncertainties and reduce errors | Med Phys | 2015 |
| 82 | Not HJV gene-related disease | Rivard, S R; Mura, C; Simard, H; Simard, R; Grimard, D; Le Gac, G; Raguenes, O; Ferec, C; De Braekeleer, M | Clinical and molecular aspects of juvenile hemochromatosis in Saguenay-Lac-Saint-Jean (Quebec, canada) | Blood Cells Mol Dis | 2000 |
| 83 | Review | O'Neil, J; Powell, L | Clinical aspects of hemochromatosis | Semin Liver Dis | 2005 |
| 84 | Review | Camaschella, C; Roetto, A; Papanikolaou, G | Commentary: Juvenile hemochromatosis in a Spanish family (by Montes-Cano et al.) | Blood Cells Mol Dis | 2002 |
| 85 | SNP study | Milet, J; Dehais, V; Bourgain, C; Jouanolle, A M; Mosser, A; Perrin, M; Morcet, J; Brissot, P; David, V; Deugnier, Y; Mosser, J | Common variants in the BMP2, BMP4, and HJV genes of the hepcidin regulation pathway modulate HFE hemochromatosis penetrance | Am J Hum Genet | 2007 |
| 86 | Experimental study | Malik, I A; Naz, N; Sheikh, N; Khan, S; Moriconi, F; Blaschke, M; Ramadori, G | Comparison of changes in gene expression of transferrin receptor-1 and other iron-regulatory proteins in rat liver and brain during acute-phase response | Cell Tissue Res | 2011 |
| 87 | Experimental study | Lin, L; Goldberg, Y P; Ganz, T | Competitive regulation of hepcidin mRNA by soluble and cell-associated hemojuvelin | Blood | 2005 |
| 88 | Experimental study | Kuninger, D; Kuns-Hashimoto, R; Kuzmickas, R; Rotwein, P | Complex biosynthesis of the muscle-enriched iron regulator RGMc | J Cell Sci | 2006 |
| 89 | Experimental study | Gkouvatsos, K; Wagner, J; Papanikolaou, G; Sebastiani, G; Pantopoulos, K | Conditional disruption of mouse HFE2 gene: maintenance of systemic iron homeostasis requires hepatic but not skeletal muscle hemojuvelin | Hepatology | 2011 |
| 90 | Experimental study | Severyn, C J; Rotwein, P | Conserved proximal promoter elements control repulsive guidance molecule c/hemojuvelin (Hfe2) gene transcription in skeletal muscle | Genomics | 2010 |
| 91 | Review | Zhang, A S | Control of systemic iron homeostasis by the hemojuvelin-hepcidin axis | Adv Nutr | 2010 |
| 92 | Not primary hemochromatosis | Aigner, E; Theurl, I; Haufe, H; Seifert, M; Hohla, F; Scharinger, L; Stickel, F; Mourlane, F; Weiss, G; Datz, C | Copper availability contributes to iron perturbations in human nonalcoholic fatty liver disease | Gastroenterology | 2008 |
| 93 | Experimental study | Ramey, G; Deschemin, J C; Vaulont, S | Cross-talk between the mitogen activated protein kinase and bone morphogenetic protein/hemojuvelin pathways is required for the induction of hepcidin by holotransferrin in primary mouse hepatocytes | Haematologica | 2009 |
| 94 | Experimental study | Yang, F; West, AP Jr; Bjorkman, P J | Crystal structure of a hemojuvelin-binding fragment of neogenin at 1.8A | J Struct Biol | 2011 |
| 95 | Not primary hemochromatosis | Borland, E M; Ledermann, J P; Powers, A M | Culex Tarsalis Mosquitoes as Vectors of Highlands J Virus | Vector Borne Zoonotic Dis | 2016 |
| 96 | Review | Brissot, P; Troadec, M B; Bardou-Jacquet, E; Le Lan, C; Jouanolle, A M; Deugnier, Y; Loreal, O | Current approach to hemochromatosis | Blood Rev | 2008 |
| 97 | Review | Brissot, P; de Bels, F | Current approaches to the management of hemochromatosis | Hematology Am Soc Hematol Educ Program | 2006 |
| 98 | Not primary hemochromatosis | Troadec, M B; Laine, F; Daniel, V; Rochcongar, P; Ropert, M; Cabillic, F; Perrin, M; Morcet, J; Loreal, O; Olbina, G; Westerman, M; Nemeth, E; Ganz, T; Brissot, P | Daily regulation of serum and urinary hepcidin is not influenced by submaximal cycling exercise in humans with normal iron metabolism | Eur J Appl Physiol | 2009 |
| 99 | Not HJV gene-related disease | Le Tertre, M; Ka, C; Guellec, J; Gourlaouen, I; Ferec, C; Callebaut, I; Le Gac, G | Deciphering the molecular basis of ferroportin resistance to hepcidin: Structure/function analysis of rare SLC40A1 missense mutations found in suspected hemochromatosis type 4 patients | Transfus Clin Biol | 2017 |
| 100 | Not primary hemochromatosis | Shucheng, G; Chunkang, C; Youshan, Z; Juan, G; Chengming, F; Xi, Z; Chao, X; Xiao, L | Decitabine treatment could ameliorate primary iron-overload in myelodysplastic syndrome patients | Cancer Invest | 2015 |
| 101 | Experimental study | Frydlova, J; Fujikura, Y; Vokurka, M; Necas, E; Krijt, J | Decreased hemojuvelin protein levels in mask mice lacking matriptase-2-dependent proteolytic activity | Physiol Res | 2013 |
| 102 | Experimental study | Silvestri, L; Pagani, A; Fazi, C; Gerardi, G; Levi, S; Arosio, P; Camaschella, C | Defective targeting of hemojuvelin to plasma membrane is a common pathogenetic mechanism in juvenile hemochromatosis | Blood | 2007 |
| 103 | Experimental study | Nick, H; Allegrini, P R; Fozard, L; Junker, U; Rojkjaer, L; Salie, R; Niederkofler, V; O'Reilly, T | Deferasirox reduces iron overload in a murine model of juvenile hemochromatosis | Exp Biol Med (Maywood) | 2009 |
| 104 | Experimental study | Li, J; Zhang, P; Liu, H; Ren, W; Song, J; Rao, E; Takahashi, E; Zhou, Y; Li, W; Chen, X | Deficits of learning and memory in Hemojuvelin knockout mice | J Vet Med Sci | 2015 |
| 105 | Experimental study | Latour, C; Besson-Fournier, C; Gourbeyre, O; Meynard, D; Roth, M P; Coppin, H | Deletion of BMP6 worsens the phenotype of HJV-deficient mice and attenuates hepcidin levels reached after LPS challenge | Blood | 2017 |
| 106 | Experimental study | Tawfik, A; Gnana-Prakasam, J P; Smith, S B; Ganapathy, V | Deletion of hemojuvelin, an iron-regulatory protein, in mice results in abnormal angiogenesis and vasculogenesis in retina along with reactive gliosis | Invest Ophthalmol Vis Sci | 2014 |
| 107 | Experimental study | Nai, A; Pagani, A; Mandelli, G; Lidonnici, M R; Silvestri, L; Ferrari, G; Camaschella, C | Deletion of TMPRSS6 attenuates the phenotype in a mouse model of beta-thalassemia | Blood | 2012 |
| 108 | Experimental study | Bian, Y H; Xu, C; Li, J; Xu, J; Zhang, H; "Du SJ" | Development of a transgenic zebrafish model expressing GFP in the notochord, somite and liver directed by the hfe2 gene promoter | Transgenic Res | 2011 |
| 109 | Experimental study | Filippi, G; Arrigoni, F; Bertini, L; De Gioia, L; Zampella, G | DFT dissection of the reduction step in H2 catalytic production by [FeFe]-hydrogenase-inspired models: can the bridging hydride become more reactive than the terminal isomer? | Inorg Chem | 2015 |
| 110 | Review | Salgia, R J; Brown, K | Diagnosis and management of hereditary hemochromatosis | Clin Liver Dis | 2015 |
| 111 | Genetic sequencing not performed | De Gobbi, M; Caruso, R; Daraio, F; Chianale, F; Pinto, R M; Longo, F; Piga, A; Camaschella, C | Diagnosis of juvenile hemochromatosis in an 11-year-old child combining genetic analysis and non-invasive liver iron quantitation | Eur J Pediatr | 2003 |
| 112 | Review | Bardou-Jacquet, E; Brissot, P | Diagnostic evaluation of hereditary hemochromatosis (HFE and non-HFE) | Hematol Oncol Clin North Am | 2014 |
| 113 | Experimental study | Latour, C; Besson-Fournier, C; Meynard, D; Silvestri, L; Gourbeyre, O; Aguilar-Martinez, P; Schmidt, P J; Fleming, M D; Roth, M P; Coppin, H | Differing impact of the deletion of hemochromatosis-associated molecules HFE and transferrin receptor-2 on the iron phenotype of mice lacking bone morphogenetic protein 6 or hemojuvelin | Hepatology | 2016 |
| 114 | Unrelated article | Carlson, M R; Gilbert-Wilson, R; Gray, D R; Mitra, J; Rauchfuss, T B; Richers, C P | Diiron Dithiolate Hydrides Complemented with Proton-Responsive Phosphine-Amine Ligands | Eur J Inorg Chem | 2017 |
| 115 | Review | Munoz, M; Garcia-Erce, J A; Remacha, A F | Disorders of iron metabolism. Part II: iron deficiency and iron overload | J Clin Pathol | 2011 |
| 116 | Not HJV gene-related disease | Barton, J C; Lee, P L | Disparate phenotypic expression of ALAS2 R452H (nt 1407 G --> A) in two brothers, one with severe sideroblastic anemia and iron overload, hepatic cirrhosis, and hepatocellular carcinoma | Blood Cells Mol Dis | 2006 |
| 117 | Experimental study | Liu, Y Q; Chang, Y Z; Zhao, B; Wang, H T; Duan, X L | Does hepatic hepcidin play an important role in exercise-associated anemia in rats? | Int J Sport Nutr Exerc Metab | 2011 |
| 118 | Review | Njajou, O T; de Jong, G; Berghuis, B; Vaessen, N; Snijders, P J; Goossens, J P; Wilson, J H; Breuning, M H; Oostra, B A; Heutink, P; Sandkuijl, L A; van Duijn, C M | Dominant hemochromatosis due to N144H mutation of SLC11A3: clinical and biological characteristics | Blood Cells Mol Dis | 2002 |
| 119 | Experimental study | Yu, P B; Hong, C C; Sachidanandan, C; Babitt, J L; Deng, D Y; Hoyng, S A; Lin, H Y; Bloch, K D; Peterson, R T | Dorsomorphin inhibits BMP signals required for embryogenesis and iron metabolism | Nat Chem Biol | 2008 |
| 120 | Experimental study | Finberg, K E; Whittlesey, R L; Fleming, M D; Andrews, N C | Down-regulation of Bmp/Smad signaling by Tmprss6 is required for maintenance of systemic iron homeostasis | Blood | 2010 |
| 121 | Experimental study | Maegdefrau, U; Arndt, S; Kivorski, G; Hellerbrand, C; Bosserhoff, A K | Downregulation of hemojuvelin prevents inhibitory effects of bone morphogenetic proteins on iron metabolism in hepatocellular carcinoma | Lab Invest | 2011 |
| 122 | Experimental study | Weizer-Stern, O; Adamsky, K; Amariglio, N; Levin, C; Koren, A; Breuer, W; Rachmilewitz, E; Breda, L; Rivella, S; Cabantchik, Z I; Rechavi, G | Downregulation of hepcidin and haemojuvelin expression in the hepatocyte cell-line HepG2 induced by thalassaemic sera | Br J Haematol | 2006 |
| 123 | Not primary hemochromatosis | Ajioka, R S; Phillips, J D; Weiss, R B; Dunn, D M; Smit, M W; Proll, S C; Katze, M G; Kushner, J P | Down-regulation of hepcidin in porphyria cutanea tarda | Blood | 2008 |
| 124 | Experimental study | Xia, Y; Cortez-Retamozo, V; Niederkofler, V; Salie, R; Chen, S; Samad, T A; Hong, C C; Arber, S; Vyas, J M; Weissleder, R; Pittet, M J; Lin, H Y | Dragon (repulsive guidance molecule b) inhibits IL-6 expression in macrophages | J Immunol | 2011 |
| 125 | Experimental study | Liu, W; Li, X; Zhao, Y; Meng, X M; Wan, C; Yang, B; Lan, H Y; Lin, H Y; Xia, Y | Dragon (repulsive guidance molecule RGMb) inhibits E-cadherin expression and induces apoptosis in renal tubular epithelial cells | J Biol Chem | 2013 |
| 126 | Not HJV gene-related disease | Le Gac, G; Mons, F; Jacolot, S; Scotet, V; Ferec, C; Frebourg, T | Early onset hereditary hemochromatosis resulting from a novel TFR2 gene nonsense mutation (R105X) in two siblings of north French descent | Br J Haematol | 2004 |
| 127 | Review |  | EASL clinical practice guidelines for HFE hemochromatosis | J Hepatol | 2010 |
| 128 | Experimental study | Frydlova, J; Rychtarcikova, Z; Gurieva, I; Vokurka, M; Truksa, J; Krijt, J | Effect of erythropoietin administration on proteins participating in iron homeostasis in Tmprss6-mutated mask mice | PLoS One | 2017 |
| 129 | Experimental study | Krijt, J; Jonasova, A; Neuwirtova, R; Necas, E | Effect of erythropoietin on hepcidin expression in hemojuvelin-mutant mice | Blood Cells Mol Dis | 2010 |
| 130 | Experimental study | Frydlova, J; Prikryl, P; Truksa, J; Falke, L L; "Du X"; Gurieva, I; Vokurka, M; Krijt, J | Effect of Erythropoietin, Iron Deficiency and Iron Overload on Liver Matriptase-2 (TMPRSS6) Protein Content in Mice and Rats | PLoS One | 2016 |
| 131 | Experimental study | Krijt, J; Frydlova, J; Kukackova, L; Fujikura, Y; Prikryl, P; Vokurka, M; Necas, E | Effect of iron overload and iron deficiency on liver hemojuvelin protein | PLoS One | 2012 |
| 132 | Experimental study | Krijt, J; Vokurka, M; Sefc, L; Duricova, D; Necas, E | Effect of lipopolysaccharide and bleeding on the expression of intestinal proteins involved in iron and haem transport | Folia Biol (Praha) | 2006 |
| 133 | Not primary hemochromatosis | Kortas, J; Prusik, K; Flis, D; Prusik, K; Ziemann, E; Leaver, N; Antosiewicz, J | Effect of Nordic Walking training on iron metabolism in elderly women | Clin Interv Aging | 2015 |
| 134 | Experimental study | Krijt, J; Niederkofler, V; Salie, R; Sefc, L; Pelichovska, T; Vokurka, M; Necas, E | Effect of phlebotomy on hepcidin expression in hemojuvelin-mutant mice | Blood Cells Mol Dis | 2007 |
| 135 | Experimental study | Zheng, Q; Guan, Y; Xia, L; Wang, Z; Jiang, Y; Zhang, X; Wang, J; Wang, G; Pu, Y; Xia, J; Luo, M | Effect of Yi Gong San Decoction on Iron Homeostasis in a Mouse Model of Acute Inflammation | Evid Based Complement Alternat Med | 2016 |
| 136 | Experimental study | Fu, C Y; Kong, Z Q; Wang, K R; Yang, Q; Zhai, K; Chen, Q; Wang, R | Effects and mechanisms of supraspinal administration of rat/mouse hemokinin-1, a mammalian tachykinin peptide, on nociception in mice | Brain Res | 2005 |
| 137 | Experimental study | Torti, S V; Lemler, E; Mueller, B K; Popp, A; Torti, F M | Effects of Anti-repulsive Guidance Molecule C (RGMc/Hemojuvelin) Antibody on Hepcidin and Iron in Mouse Liver and Tumor Xenografts | Clin Exp Pharmacol | 2016 |
| 138 | Experimental study | Rodriguez, A; Hilvo, M; Kytomaki, L; Fleming, R E; Britton, R S; Bacon, B R; Parkkila, S | Effects of iron loading on muscle: genome-wide mRNA expression profiling in the mouse | BMC Genomics | 2007 |
| 139 | Unrelated article | Zacks, M A; Paessler, S | Encephalitic alphaviruses | Vet Microbiol | 2010 |
| 140 | Experimental study | Canali, S; Zumbrennen-Bullough, K B; Core, A B; Wang, C Y; Nairz, M; Bouley, R; Swirski, F K; Babitt, J L | Endothelial cells produce bone morphogenetic protein 6 required for iron homeostasis in mice | Blood | 2017 |
| 141 | Experimental study | Gurieva, I; Frydlova, J; Rychtarcikova, Z; Vokurka, M; Truksa, J; Krijt, J | Erythropoietin administration increases splenic erythroferrone protein content and liver TMPRSS6 protein content in rats | Blood Cells Mol Dis | 2017 |
| 142 | Experimental study | Beliveau, F; Brule, C; Desilets, A; Zimmerman, B; Laporte, S A; Lavoie, C L; Leduc, R | Essential role of endocytosis of the type II transmembrane serine protease TMPRSS6 in regulating its functionality | J Biol Chem | 2011 |
| 143 | Experimental study | Merle, U; Tuma, S; Herrmann, T; Muntean, V; Volkmann, M; Gehrke, S G; Stremmel, W | Evidence for a critical role of ceruloplasmin oxidase activity in iron metabolism of Wilson disease gene knockout mice | J Gastroenterol Hepatol | 2010 |
| 144 | Experimental study | Ramos, E; Kautz, L; Rodriguez, R; Hansen, M; Gabayan, V; Ginzburg, Y; Roth, M P; Nemeth, E; Ganz, T | Evidence for distinct pathways of hepcidin regulation by acute and chronic iron loading in mice | Hepatology | 2011 |
| 145 | Experimental study | Zhang, A S; Anderson, S A; Meyers, K R; Hernandez, C; Eisenstein, R S; Enns, C A | Evidence that inhibition of hemojuvelin shedding in response to iron is mediated through neogenin | J Biol Chem | 2007 |
| 146 | Unrelated article | Allison, A B; Stallknecht, D E; Holmes, E C | Evolutionary genetics and vector adaptation of recombinant viruses of the western equine encephalitis antigenic complex provides new insights into alphavirus diversity and host switching | Virology | 2015 |
| 147 | Not HJV gene-related disease | Roetto, A; Alberti, F; Daraio, F; Cali, A; Cazzola, M; Totaro, A; Gasparini, P; Camaschella, C | Exclusion of ZIRTL as candidate gene of juvenile hemochromatosis and refinement of the critical interval on 1q21 | Blood Cells Mol Dis | 2000 |
| 148 | Review | Gnana-Prakasam, J P; Martin, P M; Smith, S B; Ganapathy, V | Expression and function of iron-regulatory proteins in retina | IUBMB Life | 2010 |
| 149 | Not HJV gene-related disease | Pelucchi, S; Mariani, R; Trombini, P; Coletti, S; Pozzi, M; Paolini, V; Barisani, D; Piperno, A | Expression of hepcidin and other iron-related genes in type 3 hemochromatosis due to a novel mutation in transferrin receptor-2 | Haematologica | 2009 |
| 150 | Experimental study | Onofre, C; Tome, F; Barbosa, C; Silva, A L; Romao, L | Expression of human Hemojuvelin (HJV) is tightly regulated by two upstream open reading frames in HJV mRNA that respond to iron overload in hepatic cells | Mol Cell Biol | 2015 |
| 151 | Experimental study | Hanninen, M M; Haapasalo, J; Haapasalo, H; Fleming, R E; Britton, R S; Bacon, B R; Parkkila, S | Expression of iron-related genes in human brain and brain tumors | BMC Neurosci | 2009 |
| 152 | Experimental study | Krijt, J; Vokurka, M; Chang, K T; Necas, E | Expression of Rgmc, the murine ortholog of hemojuvelin gene, is modulated by development and inflammation, but not by iron status or erythropoietin | Blood | 2004 |
| 153 | Experimental study | Gnana-Prakasam, J P; Zhang, M; Martin, P M; Atherton, S S; Smith, S B; Ganapathy, V | Expression of the iron-regulatory protein haemojuvelin in retina and its regulation during cytomegalovirus infection | Biochem J | 2009 |
| 154 | Experimental study | Rodriguez, A; Pan, P; Parkkila, S | Expression studies of neogenin and its ligand hemojuvelin in mouse tissues | J Histochem Cytochem | 2007 |
| 155 | Experimental study | Das, S K; Patel, V B; Basu, R; Wang, W; DesAulniers, J; Kassiri, Z; Oudit, G Y | Females Are Protected From Iron-Overload Cardiomyopathy Independent of Iron Metabolism: Key Role of Oxidative Stress | J Am Heart Assoc | 2017 |
| 156 | Review | Pietrangelo, A | Ferroportin disease: pathogenesis, diagnosis and treatment | Haematologica | 2017 |
| 157 | Experimental study | Naz, N; Malik, I A; Sheikh, N; Ahmad, S; Khan, S; Blaschke, M; Schultze, F; Ramadori, G | Ferroportin-1 is a 'nuclear'-negative acute-phase protein in rat liver: a comparison with other iron-transport proteins | Lab Invest | 2012 |
| 158 | Experimental study | Wallace, D F; Summerville, L; Lusby, P E; Subramaniam, V N | First phenotypic description of transferrin receptor 2 knockout mouse, and the role of hepcidin | Gut | 2005 |
| 159 | Not primary hemochromatosis | McDonald, C J; Ostini, L; Bennett, N; Subramaniam, N; Hooper, J; Velasco, G; Wallace, D F; Subramaniam, V N | Functional analysis of matriptase-2 mutations and domains: insights into the molecular basis of iron-refractory iron deficiency anemia | Am J Physiol Cell Physiol | 2015 |
| 160 | Not primary hemochromatosis | De Falco, L; Silvestri, L; Kannengiesser, C; Moran, E; Oudin, C; Rausa, M; Bruno, M; Aranda, J; Argiles, B; Yenicesu, I; Falcon-Rodriguez, M; Yilmaz-Keskin, E; Kocak, U; Beaumont, C; Camaschella, C; Iolascon, A; Grandchamp, B; Sanchez, M | Functional and clinical impact of novel TMPRSS6 variants in iron-refractory iron-deficiency anemia patients and genotype-phenotype studies | Hum Mutat | 2014 |
| 161 | Experimental study | Silvestri, L; Pagani, A; Camaschella, C | Furin-mediated release of soluble hemojuvelin: a new link between hypoxia and iron homeostasis | Blood | 2008 |
| 162 | Not primary hemochromatosis | Malyszko, J; Koc-Zorawska, E; Malyszko, J S; Glowinska, I; Mysliwiec, M; Macdougall, I C | GDF15 is related to anemia and hepcidin in kidney allograft recipients | Nephron Clin Pract | 2013 |
| 163 | Review | Klomp, C; Abbes, A P; Engel, H | Gene symbol: HFE2a (HJV). Disease: juvenile hemochromatosis | Hum Genet | 2005 |
| 164 | Review | Sheth, S; Brittenham, G M | Genetic disorders affecting proteins of iron metabolism: clinical implications | Annu Rev Med | 2000 |
| 165 | Genetic sequencing not performed | Papanikolaou, G; Papaioannou, M; Politou, M; Vavatsi, N; Kioumi, A; Tsiatsiou, P; Marinaki, P; Loukopoulos, D; Christakis, J I | Genetic heterogeneity underlies juvenile hemochromatosis phenotype: analysis of three families of northern Greek origin | Blood Cells Mol Dis | 2002 |
| 166 | Not primary hemochromatosis | Piesanen, JVI; Nikkari, S T; Kunnas, T A | Genetic variation in bone morphogenetic proteins family members (BMPs 2 and 4) and hypertension risk in middle-aged men: The TAMRISK study | Medicine (Baltimore) | 2017 |
| 167 | Review | Pietrangelo, A | Genetics, Genetic Testing, and Management of Hemochromatosis: 15 Years Since Hepcidin | Gastroenterology | 2015 |
| 168 | Unrelated article | Allison, A B; Stallknecht, D E | Genomic sequencing of Highlands J virus: a comparison to western and eastern equine encephalitis viruses | Virus Res | 2009 |
| 169 | Review | Brissot, P; Pietrangelo, A; Adams, P C; de Graaff, B; McLaren, C E; Loreal, O | Haemochromatosis | Nat Rev Dis Primers | 2018 |
| 170 | Review | Griffiths, W; Cox, T | Haemochromatosis: novel gene discovery and the molecular pathophysiology of iron metabolism | Hum Mol Genet | 2000 |
| 171 | Not HJV gene-related disease | Fonseca, P F; Cancado, R D; Uellendahl, Lopes MM; Correia, E; Lescano, M A; Santos, P C | HAMP Gene Mutation Associated with Juvenile Hemochromatosis in Brazilian Patients | Acta Haematol | 2016 |
| 172 | Experimental study | Yin, X; Wu, Q; Monga, J; Xie, E; Wang, H; Wang, S; Zhang, H; Wang, Z Y; Zhou, T; Shi, Y; Rogers, J; Lin, H; Min, J; Wang, F | HDAC1 Governs Iron Homeostasis Independent of Histone Deacetylation in Iron-Overload Murine Models | Antioxid Redox Signal | 2018 |
| 173 | Not HJV gene-related disease | Lee, P L; Barton, J C | Hemochromatosis and severe iron overload associated with compound heterozygosity for TFR2 R455Q and two novel mutations TFR2 R396X and G792R | Acta Haematol | 2006 |
| 174 | Review | Pietrangelo, A | Hemochromatosis: an endocrine liver disease | Hepatology | 2007 |
| 175 | Review | Zoller, H; Cox, T M | Hemochromatosis: genetic testing and clinical practice | Clin Gastroenterol Hepatol | 2005 |
| 176 | Review | Beutler, E | Hemochromatosis: genetics and pathophysiology | Annu Rev Med | 2006 |
| 177 | Genetic sequencing not performed | Cox, T M; Halsall, D J | Hemochromatosis--neonatal and young subjects | Blood Cells Mol Dis | 2002 |
| 178 | Review | Core, A B; Canali, S; Babitt, J L | Hemojuvelin and bone morphogenetic protein (BMP) signaling in iron homeostasis | Front Pharmacol | 2014 |
| 179 | Not primary hemochromatosis | Du Thanh, A; Aguilar-Martinez, P; Cunat, S; Bessis, D; Guillot, B; Dereure, O | Hemojuvelin and hepcidin gene mutations in patients with porphyria cutanea tarda from Southern France | Acta Derm Venereol | 2011 |
| 180 | SNP study | de Lima, Santos PC; Pereira, A C; Cancado, R D; Schettert, I T; Hirata, R D; Hirata, M H; Figueiredo, M S; Chiattone, C S; Krieger, J E; Guerra-Shinohara, E M | Hemojuvelin and hepcidin genes sequencing in Brazilian patients with primary iron overload | Genet Test Mol Biomarkers | 2010 |
| 181 | Not primary hemochromatosis | Przybylowski, P; Koc-Zorawska, E; Glowinska, I; Levin-Iaina, N; Macdougall, I C; Malyszko, J S; Mysliwiec, M; Malyszko, J | Hemojuvelin and iron metabolism in kidney and heart allograft recipients | Transplant Proc | 2013 |
| 182 | Experimental study | Niederkofler, V; Salie, R; Arber, S | Hemojuvelin is essential for dietary iron sensing, and its mutation leads to severe iron overload | J Clin Invest | 2005 |
| 183 | Experimental study | Bartnikas, T B; Fleming, M D | Hemojuvelin is essential for transferrin-dependent and transferrin-independent hepcidin expression in mice | Haematologica | 2012 |
| 184 | Experimental study | Young, G H; Huang, T M; Wu, C H; Lai, C F; Hou, C C; Peng, K Y; Liang, C J; Lin, S L; Chang, S C; Tsai, P R; Wu, K D; Wu, V C; Ko, W J | Hemojuvelin modulates iron stress during acute kidney injury: improved by furin inhibitor | Antioxid Redox Signal | 2014 |
| 185 | Experimental study | Pagani, A; Silvestri, L; Nai, A; Camaschella, C | Hemojuvelin N-terminal mutants reach the plasma membrane but do not activate the hepcidin response | Haematologica | 2008 |
| 186 | Not primary hemochromatosis | Ko, S W; Chi, N H; Wu, C H; Huang, T M; Chueh, S J; Wang, C H; Lin, J H; Wang, W J; Ting, J T; Chang, H M; Connolly, R; Lai, C H; Tseng, L J; Wu, V C; Chu, T S | Hemojuvelin Predicts Acute Kidney Injury and Poor Outcomes Following Cardiac Surgery | Sci Rep | 2018 |
| 187 | Experimental study | Xia, Y; Babitt, J L; Sidis, Y; Chung, R T; Lin, H Y | Hemojuvelin regulates hepcidin expression via a selective subset of BMP ligands and receptors independently of neogenin | Blood | 2008 |
| 188 | Experimental study | Wu, Q; Shen, Y; Tao, Y; Wei, J; Wang, H; An, P; Zhang, Z; Gao, H; Zhou, T; Wang, F; Min, J | Hemojuvelin regulates the innate immune response to peritoneal bacterial infection in mice | Cell Discov | 2017 |
| 189 | Not primary hemochromatosis | Luciani, N; Brasse-Lagnel, C; Poli, M; Anty, R; Lesueur, C; Cormont, M; Laquerriere, A; Folope, V; LeMarchand-Brustel, Y; Gugenheim, J; Gual, P; Tran, A; Bekri, S | Hemojuvelin: a new link between obesity and iron homeostasis | Obesity (Silver Spring) | 2011 |
| 190 | Review | Celec, P | Hemojuvelin: a supposed role in iron metabolism one year after its discovery | J Mol Med (Berl) | 2005 |
| 191 | Review | Malyszko, J | Hemojuvelin: the hepcidin story continues | Kidney Blood Press Res | 2009 |
| 192 | Not primary hemochromatosis | Formanowicz, D; Kozak, A; Glowacki, T; Radom, M; Formanowicz, P | Hemojuvelin-hepcidin axis modeled and analyzed using Petri nets | J Biomed Inform | 2013 |
| 193 | Experimental study | Zhang, A S; Yang, F; Wang, J; Tsukamoto, H; Enns, C A | Hemojuvelin-neogenin interaction is required for bone morphogenic protein-4-induced hepcidin expression | J Biol Chem | 2009 |
| 194 | Experimental study | Rodriguez, Martinez A; Niemela, O; Parkkila, S | Hepatic and extrahepatic expression of the new iron regulatory protein hemojuvelin | Haematologica | 2004 |
| 195 | Experimental study | Bondi, A; Valentino, P; Daraio, F; Porporato, P; Gramaglia, E; Carturan, S; Gottardi, E; Camaschella, C; Roetto, A | Hepatic expression of hemochromatosis genes in two mouse strains after phlebotomy and iron overload | Haematologica | 2005 |
| 196 | Genetic sequencing not performed | Gleeson, F; Ryan, E; Barrett, S; Russell, J; Crowe, J | Hepatic iron metabolism gene expression profiles in HFE associated hereditary hemochromatosis | Blood Cells Mol Dis | 2007 |
| 197 | Experimental study | Maurer, E; Gutschow, M; Stirnberg, M | Hepatocyte growth factor activator inhibitor type 2 (HAI-2) modulates hepcidin expression by inhibiting the cell surface protease matriptase-2 | Biochem J | 2013 |
| 198 | Experimental study | Gao, J; Chen, J; De Domenico, I; Koeller, D M; Harding, C O; Fleming, R E; Koeberl, D D; Enns, C A | Hepatocyte-targeted HFE and TFR2 control hepcidin expression in mice | Blood | 2010 |
| 199 | Experimental study | Sheikh, N; Batusic, D S; Dudas, J; Tron, K; Neubauer, K; Saile, B; Ramadori, G | Hepcidin and hemojuvelin gene expression in rat liver damage: in vivo and in vitro studies | Am J Physiol Gastrointest Liver Physiol | 2006 |
| 200 | Review | Poli, M; Asperti, M; Ruzzenenti, P; Regoni, M; Arosio, P | Hepcidin antagonists for potential treatments of disorders with hepcidin excess | Front Pharmacol | 2014 |
| 201 | Experimental study | Krijt, J; Fujikura, Y; Sefc, L; Vokurka, M; Hlobenova, T; Necas, E | Hepcidin downregulation by repeated bleeding is not mediated by soluble hemojuvelin | Physiol Res | 2010 |
| 202 | Experimental study | Ishizaki, N; Kotani, M; Funaba, M; Matsui, T | Hepcidin expression in the liver of rats fed a magnesium-deficient diet | Br J Nutr | 2011 |
| 203 | Genetic sequencing not performed | Papanikolaou, G; Tzilianos, M; Christakis, J I; Bogdanos, D; Tsimirika, K; MacFarlane, J; Goldberg, Y P; Sakellaropoulos, N; Ganz, T; Nemeth, E | Hepcidin in iron overload disorders | Blood | 2005 |
| 204 | Experimental study | Schmidt, P J; Andrews, N C; Fleming, M D | Hepcidin induction by transgenic overexpression of Hfe does not require the Hfe cytoplasmic tail, but does require hemojuvelin | Blood | 2010 |
| 205 | Not primary hemochromatosis | Finkenstedt, A; Widschwendter, A; Brasse-Lagnel, C G; Theurl, I; Hubalek, M; Dieplinger, H; Tselepis, C; Ward, D G; Vogel, W; Zoller, H | Hepcidin is correlated to soluble hemojuvelin but not to increased GDF15 during pregnancy | Blood Cells Mol Dis | 2012 |
| 206 | Not HJV gene-related disease | Nemeth, E; Roetto, A; Garozzo, G; Ganz, T; Camaschella, C | Hepcidin is decreased in TFR2 hemochromatosis | Blood | 2005 |
| 207 | Not primary hemochromatosis | Slomka, A; Switonska, M; Zekanowska, E | Hepcidin Levels Are Increased in Patients with Acute Ischemic Stroke: Preliminary Report | J Stroke Cerebrovasc Dis | 2015 |
| 208 | Experimental study | Vokurka, M; Krijt, J; Sulc, K; Necas, E | Hepcidin mRNA levels in mouse liver respond to inhibition of erythropoiesis | Physiol Res | 2006 |
| 209 | Experimental study | Moulouel, B; Houamel, D; Delaby, C; Tchernitchko, D; Vaulont, S; Letteron, P; Thibaudeau, O; Puy, H; Gouya, L; Beaumont, C; Karim, Z | Hepcidin regulates intrarenal iron handling at the distal nephron | Kidney Int | 2013 |
| 210 | Experimental study | Wu, X; Yung, L M; Cheng, W H; Yu, P B; Babitt, J L; Lin, H Y; Xia, Y | Hepcidin regulation by BMP signaling in macrophages is lipopolysaccharide dependent | PLoS One | 2012 |
| 211 | Review | De Domenico, I; Ward, D M; Kaplan, J | Hepcidin regulation: ironing out the details | J Clin Invest | 2007 |
| 212 | Review | Ganz, T | Hepcidin, a key regulator of iron metabolism and mediator of anemia of inflammation | Blood | 2003 |
| 213 | Review | Viatte, L; Vaulont, S | Hepcidin, the iron watcher | Biochimie | 2009 |
| 214 | Not primary hemochromatosis | Kohjima, M; Yoshimoto, T; Enjoji, M; Fukushima, N; Fukuizumi, K; Nakamura, T; Kurokawa, M; Fujimori, N; Sasaki, Y; Shimonaka, Y; Murata, Y; Koyama, S; Kawabe, K; Haraguchi, K; Sumida, Y; Harada, N; Kato, M; Kotoh, K; Nakamuta, M | Hepcidin/ferroportin expression levels involve efficacy of pegylated-interferon plus ribavirin in hepatitis C virus-infected liver | World J Gastroenterol | 2015 |
| 215 | Review | Pietrangelo, A | Hereditary hemochromatosis | Biochim Biophys Acta | 2006 |
| 216 | Not HJV gene-related disease | Voicu, P M; Cojocariu, C; Petrescu-Danila, E; Stanciu, C; Covic, M; Rusu, M; Trifan, A | Hereditary hemochromatosis in north-eastern Romania | Rev Med Chir Soc Med Nat Iasi | 2010 |
| 217 | Experimental study | Miller, H K; Schwiesow, L; Au-Yeung, W; Auerbuch, V | Hereditary Hemochromatosis Predisposes Mice to Yersinia pseudotuberculosis Infection Even in the Absence of the Type III Secretion System | Front Cell Infect Microbiol | 2016 |
| 218 | Experimental study | Quenee, L E; Hermanas, T M; Ciletti, N; Louvel, H; Miller, N C; Elli, D; Blaylock, B; Mitchell, A; Schroeder, J; Krausz, T; Kanabrocki, J; Schneewind, O | Hereditary hemochromatosis restores the virulence of plague vaccine strains | J Infect Dis | 2012 |
| 219 | Review | Alexander, J; Kowdley, K V | Hereditary hemochromatosis: genetics, pathogenesis, and clinical management | Ann Hepatol | 2005 |
| 220 | Not HJV gene-related disease | Papanikolaou, G; Politou, M; Terpos, E; Fourlemadis, S; Sakellaropoulos, N; Loukopoulos, D | Hereditary hemochromatosis: HFE mutation analysis in Greeks reveals genetic heterogeneity | Blood Cells Mol Dis | 2000 |
| 221 | Review | Gasparini, P; Camaschella, C | Hereditary hemochromatosis: is the gene race over? | Eur J Hum Genet | 2004 |
| 222 | Review | Pietrangelo, A | Hereditary hemochromatosis: pathogenesis, diagnosis, and treatment | Gastroenterology | 2010 |
| 223 | Review | Franchini, M | Hereditary iron overload: update on pathophysiology, diagnosis, and treatment | Am J Hematol | 2006 |
| 224 | Experimental study | Kent, P; Wilkinson, N; Constante, M; Fillebeen, C; Gkouvatsos, K; Wagner, J; Buffler, M; Becker, C; Schumann, K; Santos, M M; Pantopoulos, K | Hfe and Hjv exhibit overlapping functions for iron signaling to hepcidin | J Mol Med (Berl) | 2015 |
| 225 | Not primary hemochromatosis | Shalev, H; Perez-Avraham, G; Kapelushnik, J; Levi, I; Rabinovich, A; Swinkels, D W; Brasse-Lagnel, C; Tamary, H | High levels of soluble serum hemojuvelin in patients with congenital dyserythropoietic anemia type I | Eur J Haematol | 2013 |
| 226 | Review | Wu, Q; Wang, H; An, P; Tao, Y; Deng, J; Zhang, Z; Shen, Y; Chen, C; Min, J; Wang, F | HJV and HFE Play Distinct Roles in Regulating Hepcidin | Antioxid Redox Signal | 2015 |
| 227 | Unrelated article |  | HlV/STD risks in young men who have sex with men who do not disclose their sexual orientation--six U.S. cities, 1994-2000 | MMWR Morb Mortal Wkly Rep | 2003 |
| 228 | Experimental study | Shi, W; Wang, H; Zheng, X; Jiang, X; Xu, Z; Shen, H; Li, M | HNF-4alpha Negatively Regulates Hepcidin Expression Through BMPR1A in HepG2 Cells | Biol Trace Elem Res | 2017 |
| 229 | Experimental study | Chaston, T B; Matak, P; Pourvali, K; Srai, S K; McKie, A T; Sharp, P A | Hypoxia inhibits hepcidin expression in HuH7 hepatoma cells via decreased SMAD4 signaling | Am J Physiol Cell Physiol | 2011 |
| 230 | Not HJV gene-related disease | Hattori, A; Tomosugi, N; Tatsumi, Y; Suzuki, A; Hayashi, K; Katano, Y; Inagaki, Y; Ishikawa, T; Hayashi, H; Goto, H; Wakusawa, S | Identification of a novel mutation in the HAMP gene that causes non-detectable hepcidin molecules in a Japanese male patient with juvenile hemochromatosis | Blood Cells Mol Dis | 2012 |
| 231 | Review | Gerhard, G S; Paynton, B V; DiStefano, J K | Identification of Genes for Hereditary Hemochromatosis | Methods Mol Biol | 2018 |
| 232 | Not HJV gene-related disease | Piubelli, C; Castagna, A; Marchi, G; Rizzi, M; Busti, F; Badar, S; Marchetti, M; De Gobbi, M; Roetto, A; Xumerle, L; Suku, E; Giorgetti, A; Delledonne, M; Olivieri, O; Girelli, D | Identification of new BMP6 pro-peptide mutations in patients with iron overload | Am J Hematol | 2017 |
| 233 | Experimental study | Rausa, M; Ghitti, M; Pagani, A; Nai, A; Campanella, A; Musco, G; Camaschella, C; Silvestri, L | Identification of TMPRSS6 cleavage sites of hemojuvelin | J Cell Mol Med | 2015 |
| 234 | Experimental study | Brasse-Lagnel, C; Poli, M; Lesueur, C; Grandchamp, B; Lavoinne, A; Beaumont, C; Bekri, S | Immunoassay for human serum hemojuvelin | Haematologica | 2010 |
| 235 | Experimental study | Ganz, T; Olbina, G; Girelli, D; Nemeth, E; Westerman, M | Immunoassay for human serum hepcidin | Blood | 2008 |
| 236 | Unrelated article | Valentijn, T M; Hoeks, S E; Martienus, K A; Bakker, E J; van de Luijtgaarden, K M; Verhagen, H J; Stolker, R J; van Lier, F | Impact of haemoglobin concentration on cardiovascular outcome after vascular surgery: a retrospective observational cohort study | Eur J Anaesthesiol | 2013 |
| 237 | Experimental study | Schaefer, B; Haschka, D; Finkenstedt, A; Petersen, B S; Theurl, I; Henninger, B; Janecke, A R; Wang, C Y; Lin, H Y; Veits, L; Vogel, W; Weiss, G; Franke, A; Zoller, H | Impaired hepcidin expression in alpha-1-antitrypsin deficiency associated with iron overload and progressive liver disease | Hum Mol Genet | 2015 |
| 238 | Experimental study | Hofer, T; Marzetti, E; Xu, J; Seo, A Y; Gulec, S; Knutson, M D; Leeuwenburgh, C; Dupont-Versteegden, E E | Increased iron content and RNA oxidative damage in skeletal muscle with aging and disuse atrophy | Exp Gerontol | 2008 |
| 239 | Experimental study | Arjunan, P; Gnanaprakasam, J P; Ananth, S; Romej, M A; Rajalakshmi, V K; Prasad, P D; Martin, P M; Gurusamy, M; Thangaraju, M; Bhutia, Y D; Ganapathy, V | Increased Retinal Expression of the Pro-Angiogenic Receptor GPR91 via BMP6 in a Mouse Model of Juvenile Hemochromatosis | Invest Ophthalmol Vis Sci | 2016 |
| 240 | Review | Camaschella, C; Poggiali, E | Inherited disorders of iron metabolism | Curr Opin Pediatr | 2011 |
| 241 | Genetic sequencing not performed | Camaschella, C; Fargion, S; Sampietro, M; Roetto, A; Bosio, S; Garozzo, G; Arosio, C; Piperno, A | Inherited HFE-unrelated hemochromatosis in Italian families | Hepatology | 1999 |
| 242 | Review | Worwood, M | Inherited iron loading: genetic testing in diagnosis and management | Blood Rev | 2005 |
| 243 | Review | Ala, A; Schilsky, M L | Inherited metabolic liver disease | Curr Opin Gastroenterol | 2004 |
| 244 | Review | Darshan, D; Anderson, G J | Interacting signals in the control of hepcidin expression | Biometals | 2009 |
| 245 | Experimental study | Zhang, A S; West, AP Jr; Wyman, A E; Bjorkman, P J; Enns, C A | Interaction of hemojuvelin with neogenin results in iron accumulation in human embryonic kidney 293 cells | J Biol Chem | 2005 |
| 246 | Experimental study | Otto-Duessel, M; Brewer, C; Wood, J C | Interdependence of cardiac iron and calcium in a murine model of iron overload | Transl Res | 2011 |
| 247 | Review | Knutson, M D | Into the matrix: regulation of the iron regulatory hormone hepcidin by matriptase-2 | Nutr Rev | 2009 |
| 248 | Review | Anderson, G J; Frazer, D M; McLaren, G D | Iron absorption and metabolism | Curr Opin Gastroenterol | 2009 |
| 249 | Experimental study | Deugnier, Y; Brissot, P; Loreal, O | Iron and the liver: update 2008 | J Hepatol | 2008 |
| 250 | Review | Brissot, P; Bardou-Jacquet, E; Jouanolle, A M; Loreal, O | Iron disorders of genetic origin: a changing world | Trends Mol Med | 2011 |
| 251 | Review | Brissot, P; Loreal, O | Iron metabolism and related genetic diseases: A cleared land, keeping mysteries | J Hepatol | 2016 |
| 252 | Not primary hemochromatosis | Polonifi, A; Politou, M; Kalotychou, V; Xiromeritis, K; Tsironi, M; Berdoukas, V; Vaiopoulos, G; Aessopos, A | Iron metabolism gene expression in human skeletal muscle | Blood Cells Mol Dis | 2010 |
| 253 | Not primary hemochromatosis | Malyszko, J; Koc-Zorawska, E; Levin-Iaina, N; Slotki, I; Matuszkiewicz-Rowinska, J; Glowinska, I; Malyszko, J S | Iron metabolism in hemodialyzed patients - a story half told? | Arch Med Sci | 2014 |
| 254 | Review | Anderson, G J; Frazer, D M | Iron metabolism meets signal transduction | Nat Genet | 2006 |
| 255 | Review | Siddaiah, N; Kowdley, K V | Iron Overload (with Attention to Genetic Testing and Diagnosis/Management of HFE Wild Type Patients) | Curr Treat Options Gastroenterol | 2006 |
| 256 | Not primary hemochromatosis | Barton, J C; Lee, P L; Bertoli, L F; Beutler, E | Iron overload in an African American woman with SS hemoglobinopathy and a promoter mutation in the X-linked erythroid-specific 5-aminolevulinate synthase (ALAS2) gene | Blood Cells Mol Dis | 2005 |
| 257 | Review | Batts, K P | Iron overload syndromes and the liver | Mod Pathol | 2007 |
| 258 | Experimental study | Chirumbolo, S; Rossi, A P; Rizzatti, V; Zoico, E; Franceschetti, G; Girelli, D; Zamboni, M | Iron primes 3T3-L1 adipocytes to a TLR4-mediated inflammatory response | Nutrition | 2015 |
| 259 | Review | De Falco, L; Sanchez, M; Silvestri, L; Kannengiesser, C; Muckenthaler, M U; Iolascon, A; Gouya, L; Camaschella, C; Beaumont, C | Iron refractory iron deficiency anemia | Haematologica | 2013 |
| 260 | Review | Nemeth, E | Iron regulation and erythropoiesis | Curr Opin Hematol | 2008 |
| 261 | Review | Zhao, N; Zhang, A S; Enns, C A | Iron regulation by hepcidin | J Clin Invest | 2013 |
| 262 | Review | Pantopoulos, K | Iron regulation of hepcidin through Hfe and Hjv: Common or distinct pathways? | Hepatology | 2015 |
| 263 | Not primary hemochromatosis | Tsuchiya, H; Ashla, A A; Hoshikawa, Y; Matsumi, Y; Kanki, K; Enjoji, M; Momosaki, S; Nakamuta, M; Taketomi, A; Maehara, Y; Shomori, K; Kurimasa, A; Hisatome, I; Ito, H; Shiota, G | Iron state in association with retinoid metabolism in non-alcoholic fatty liver disease | Hepatol Res | 2010 |
| 264 | Not primary hemochromatosis | Lukaszyk, E; Lukaszyk, M; Koc-Zorawska, E; Tobolczyk, J; Bodzenta-Lukaszyk, A; Malyszko, J | Iron Status and Inflammation in Early Stages of Chronic Kidney Disease | Kidney Blood Press Res | 2015 |
| 265 | Review | McDonald, C J; Wallace, D F; Crawford, D H; Subramaniam, V N | Iron storage disease in Asia-Pacific populations: the importance of non-HFE mutations | J Gastroenterol Hepatol | 2013 |
| 266 | Genetic sequencing not performed | Gehrke, S G; Herrmann, T; Kulaksiz, H; Merle, U; Bents, K; Kaiser, I; Riedel, H D; Stremmel, W | Iron stores modulate hepatic hepcidin expression by an HFE-independent pathway | Digestion | 2005 |
| 267 | Experimental study | Lin, L; Valore, E V; Nemeth, E; Goodnough, J B; Gabayan, V; Ganz, T | Iron transferrin regulates hepcidin synthesis in primary hepatocyte culture through hemojuvelin and BMP2/4 | Blood | 2007 |
| 268 | Review | Dunn, L L; Suryo, Rahmanto Y; Richardson, D R | Iron uptake and metabolism in the new millennium | Trends Cell Biol | 2007 |
| 269 | Experimental study | Lenoir, A; Deschemin, J C; Kautz, L; Ramsay, A J; Roth, M P; Lopez-Otin, C; Vaulont, S; Nicolas, G | Iron-deficiency anemia from matriptase-2 inactivation is dependent on the presence of functional Bmp6 | Blood | 2011 |
| 270 | Experimental study | Gkouvatsos, K; Fillebeen, C; Daba, A; Wagner, J; Sebastiani, G; Pantopoulos, K | Iron-dependent regulation of hepcidin in Hjv-/- mice: evidence that hemojuvelin is dispensable for sensing body iron levels | PLoS One | 2014 |
| 271 | Experimental study | Gnana-Prakasam, J P; Tawfik, A; Romej, M; Ananth, S; Martin, P M; Smith, S B; Ganapathy, V | Iron-mediated retinal degeneration in haemojuvelin-knockout mice | Biochem J | 2012 |
| 272 | Experimental study | Das, S K; Wang, W; Zhabyeyev, P; Basu, R; McLean, B; Fan, D; Parajuli, N; DesAulniers, J; Patel, V B; Hajjar, R J; Dyck, J R; Kassiri, Z; Oudit, G Y | Iron-overload injury and cardiomyopathy in acquired and genetic models is attenuated by resveratrol therapy | Sci Rep | 2015 |
| 273 | Review | Finberg, K E | Iron-refractory iron deficiency anemia | Semin Hematol | 2009 |
| 274 | Experimental study | Mollbrink, A; Holmstrom, P; Sjostrom, M; Hultcrantz, R; Eriksson, L C; Stal, P | Iron-regulatory gene expression during liver regeneration | Scand J Gastroenterol | 2012 |
| 275 | Not primary hemochromatosis | Malyszko, J; Malyszko, J S; Levin-Iaina, N; Koc-Zorawska, E; Kozminski, P; Mysliwiec, M | Is hemojuvelin a possible new player in iron metabolism in hemodialysis patients? | Int Urol Nephrol | 2012 |
| 276 | Unrelated article | Barton, B E; Zampella, G; Justice, A K; De Gioia, L; Rauchfuss, T B; Wilson, S R | Isomerization of the hydride complexes [HFe2(SR)2(PR3)(x)(CO)(6-x)]+ (x = 2, 3, 4) relevant to the active site models for the [FeFe]-hydrogenases | Dalton Trans | 2010 |
| 277 | Genetic sequencing not performed | Camaschella, C; Roetto, A; Cicilano, M; Pasquero, P; Bosio, S; Gubetta, L; Di Vito, F; Girelli, D; Totaro, A; Carella, M; Grifa, A; Gasparini, P | Juvenile and adult hemochromatosis are distinct genetic disorders | Eur J Hum Genet | 1997 |
| 278 | Review | Pietrangelo, A | Juvenile hemochromatosis | J Hepatol | 2006 |
| 279 | Review | Camaschella, C; Roetto, A; De Gobbi, M | Juvenile hemochromatosis | Semin Hematol | 2002 |
| 280 | Genetic sequencing not performed | Montes-Cano, M; Gonzalez-Escribano, M F; Aguilar, J; Nunez-Roldan, A | Juvenile hemochromatosis in a Spanish family | Blood Cells Mol Dis | 2002 |
| 281 | Genetic sequencing not performed | Barton, J C; Rao, S V; Pereira, N M; Gelbart, T; Beutler, E; Rivers, C A; Acton, R T | Juvenile hemochromatosis in the southeastern United States: a report of seven cases in two kinships | Blood Cells Mol Dis | 2002 |
| 282 | Genetic sequencing not performed | Roetto, A; Totaro, A; Cazzola, M; Cicilano, M; Bosio, S; D'Ascola, G; Carella, M; Zelante, L; Kelly, A L; Cox, T M; Gasparini, P; Camaschella, C | Juvenile hemochromatosis locus maps to chromosome 1q | Am J Hum Genet | 1999 |
| 283 | Genetic sequencing not performed | Rivard, S R; Lanzara, C; Grimard, D; Carella, M; Simard, H; Ficarella, R; Simard, R; D'Adamo, A P; Ferec, C; Camaschella, C; Mura, C; Roetto, A; De Braekeleer, M; Bechner, L; Gasparini, P | Juvenile hemochromatosis locus maps to chromosome 1q in a French Canadian population | Eur J Hum Genet | 2003 |
| 284 | Not HJV gene-related disease | Lescano, M A; Tavares, L C; Santos, PCJL | Juvenile hemochromatosis: HAMP mutation and severe iron overload treated with phlebotomies and deferasirox | World J Clin Cases | 2017 |
| 285 | Review | Goldberg, Y P | Juvenile Hereditary Hemochromatosis |  | 1993 |
| 286 | Experimental study | Theurl, M; Theurl, I; Hochegger, K; Obrist, P; Subramaniam, N; van Rooijen, N; Schuemann, K; Weiss, G | Kupffer cells modulate iron homeostasis in mice via regulation of hepcidin expression | J Mol Med (Berl) | 2008 |
| 287 | Experimental study | Meynard, D; Kautz, L; Darnaud, V; Canonne-Hergaux, F; Coppin, H; Roth, M P | Lack of the bone morphogenetic protein BMP6 induces massive iron overload | Nat Genet | 2009 |
| 288 | Experimental study | Lefebvre, T; Dessendier, N; Houamel, D; Ialy-Radio, N; Kannengiesser, C; Manceau, H; Beaumont, C; Nicolas, G; Gouya, L; Puy, H; Karim, Z | LC-MS/MS method for hepcidin-25 measurement in human and mouse serum: clinical and research implications in iron disorders | Clin Chem Lab Med | 2015 |
| 289 | Genetic sequencing not performed | Papanikolaou, G; Politou, M; Roetto, A; Bosio, S; Sakelaropoulos, N; Camaschella, C; Loukopoulos, D | Linkage to chromosome 1q in Greek families with juvenile hemochromatosis | Blood Cells Mol Dis | 2001 |
| 290 | Experimental study | Fujikura, Y; Krijt, J; Necas, E | Liver and muscle hemojuvelin are differently glycosylated | BMC Biochem | 2011 |
| 291 | Review | Ovchinsky, N; Moreira, R K; Lefkowitch, J H; Lavine, J E | Liver biopsy in modern clinical practice: a pediatric point-of-view | Adv Anat Pathol | 2012 |
| 292 | Experimental study | De Franceschi, L; Daraio, F; Filippini, A; Carturan, S; Muchitsch, E M; Roetto, A; Camaschella, C | Liver expression of hepcidin and other iron genes in two mouse models of beta-thalassemia | Haematologica | 2006 |
| 293 | Experimental study | Krijt, J; Fujikura, Y; Ramsay, A J; Velasco, G; Necas, E | Liver hemojuvelin protein levels in mice deficient in matriptase-2 (Tmprss6) | Blood Cells Mol Dis | 2011 |
| 294 | Experimental study | Frydlova, J; Rogalsky, D W; Truksa, J; Traeger, L; Steinbicker, A U; Vokurka, M; Krijt, J | Liver HFE protein content is posttranscriptionally decreased in iron-deficient mice and rats | Am J Physiol Gastrointest Liver Physiol | 2018 |
| 295 | Review | Graham, R M; Chua, A C; Herbison, C E; Olynyk, J K; Trinder, D | Liver iron transport | World J Gastroenterol | 2007 |
| 296 | Experimental study | Merle, U; Theilig, F; Fein, E; Gehrke, S; Kallinowski, B; Riedel, H D; Bachmann, S; Stremmel, W; Kulaksiz, H | Localization of the iron-regulatory proteins hemojuvelin and transferrin receptor 2 to the basolateral membrane domain of hepatocytes | Histochem Cell Biol | 2007 |
| 297 | Experimental study | Xu, J; Hwang, J C; Lees, H A; Wohlgemuth, S E; Knutson, M D; Judge, A R; Dupont-Versteegden, E E; Marzetti, E; Leeuwenburgh, C | Long-term perturbation of muscle iron homeostasis following hindlimb suspension in old rats is associated with high levels of oxidative stress and impaired recovery from atrophy | Exp Gerontol | 2012 |
| 298 | Experimental study | Gnana-Prakasam, J P; Veeranan-Karmegam, R; Coothankandaswamy, V; Reddy, S K; Martin, P M; Thangaraju, M; Smith, S B; Ganapathy, V | Loss of Hfe leads to progression of tumor phenotype in primary retinal pigment epithelial cells | Invest Ophthalmol Vis Sci | 2013 |
| 299 | Experimental study | Zhao, N; Nizzi, C P; Anderson, S A; Wang, J; Ueno, A; Tsukamoto, H; Eisenstein, R S; Enns, C A; Zhang, A S | Low intracellular iron increases the stability of matriptase-2 | J Biol Chem | 2015 |
| 300 | Review | Ramsay, A J; Hooper, J D; Folgueras, A R; Velasco, G; Lopez-Otin, C | Matriptase-2 (TMPRSS6): a proteolytic regulator of iron homeostasis | Haematologica | 2009 |
| 301 | Experimental study | Maxson, J E; Chen, J; Enns, C A; Zhang, A S | Matriptase-2- and proprotein convertase-cleaved forms of hemojuvelin have different roles in the down-regulation of hepcidin expression | J Biol Chem | 2010 |
| 302 | Experimental study | Folgueras, A R; Freitas-Rodriguez, S; Ramsay, A J; Garabaya, C; Rodriguez, F; Velasco, G; Lopez-Otin, C | Matriptase-2 deficiency protects from obesity by modulating iron homeostasis | Nat Commun | 2018 |
| 303 | Experimental study | Willemetz, A; Lenoir, A; Deschemin, J C; Lopez-Otin, C; Ramsay, A J; Vaulont, S; Nicolas, G | Matriptase-2 is essential for hepcidin repression during fetal life and postnatal development in mice to maintain iron homeostasis | Blood | 2014 |
| 304 | Experimental study | Wahedi, M; Wortham, A M; Kleven, M D; Zhao, N; Jue, S; Enns, C A; Zhang, A S | Matriptase-2 suppresses hepcidin expression by cleaving multiple components of the hepcidin induction pathway | J Biol Chem | 2017 |
| 305 | Review | Stirnberg, M; Gutschow, M | Matriptase-2, a regulatory protease of iron homeostasis: possible substrates, cleavage sites and inhibitors | Curr Pharm Des | 2013 |
| 306 | Review | Pietrangelo, A; Trautwein, C | Mechanisms of disease: The role of hepcidin in iron homeostasis--implications for hemochromatosis and other disorders | Nat Clin Pract Gastroenterol Hepatol | 2004 |
| 307 | Review | Anderson, G J; Frazer, D M; McKie, A T; Vulpe, C D; Smith, A | Mechanisms of haem and non-haem iron absorption: lessons from inherited disorders of iron metabolism | Biometals | 2005 |
| 308 | Not HJV gene-related disease | Lee, P L; Gaasterland, T; Barton, J C | Mild iron overload in an African American man with SLC40A1 D270V | Acta Haematol | 2012 |
| 309 | Not primary hemochromatosis | Nikkari, S T; Visto, A L; Maatta, K M; Kunnas, T A | Minor variant of rs 16827043 in the iron regulator hemojuvelin gene (HJV) contributes to hypertension: The TAMRISK study | Medicine (Baltimore) | 2017 |
| 310 | Review | Huang, X P; O'Brien, P J; Templeton, D M | Mitochondrial involvement in genetically determined transition metal toxicity I. Iron toxicity | Chem Biol Interact | 2006 |
| 311 | Experimental study | St-Georges, C; Desilets, A; Beliveau, F; Ghinet, M; Dion, S P; Colombo, E; Boudreault, P L; Najmanovich, R J; Leduc, R; Marsault, E | Modulating the selectivity of matriptase-2 inhibitors with unnatural amino acids | Eur J Med Chem | 2017 |
| 312 | Experimental study | Babitt, J L; Huang, F W; Xia, Y; Sidis, Y; Andrews, N C; Lin, H Y | Modulation of bone morphogenetic protein signaling in vivo regulates systemic iron balance | J Clin Invest | 2007 |
| 313 | Review | Darshan, D; Frazer, D M; Anderson, G J | Molecular basis of iron-loading disorders | Expert Rev Mol Med | 2010 |
| 314 | Review | Santos, P C; Krieger, J E; Pereira, A C | Molecular diagnostic and pathogenesis of hereditary hemochromatosis | Int J Mol Sci | 2012 |
| 315 | Experimental study | Hilton, K B; Lambert, L A | Molecular evolution and characterization of hepcidin gene products in vertebrates | Gene | 2008 |
| 316 | Experimental study | Camus, L M; Lambert, L A | Molecular evolution of hemojuvelin and the repulsive guidance molecule family | J Mol Evol | 2007 |
| 317 | Review | Andrews, N C; Fleming, M D; Levy, J E | Molecular insights into mechanisms of iron transport | Curr Opin Hematol | 1999 |
| 318 | Review | Pietrangelo, A | Molecular insights into the pathogenesis of hereditary haemochromatosis | Gut | 2006 |
| 319 | Not HJV gene-related disease | Rideau, A; Mangeat, B; Matthes, T; Trono, D; Beris, P | Molecular mechanism of hepcidin deficiency in a patient with juvenile hemochromatosis | Haematologica | 2007 |
| 320 | Experimental study | Neves, J V; Caldas, C; Wilson, J M; Rodrigues, P N | Molecular mechanisms of hepcidin regulation in sea bass (Dicentrarchus labrax) | Fish Shellfish Immunol | 2011 |
| 321 | Review | Richardson, D R | Molecular mechanisms of iron uptake by cells and the use of iron chelators for the treatment of cancer | Curr Med Chem | 2005 |
| 322 | Experimental study | Silvestri, L; Guillem, F; Pagani, A; Nai, A; Oudin, C; Silva, M; Toutain, F; Kannengiesser, C; Beaumont, C; Camaschella, C; Grandchamp, B | Molecular mechanisms of the defective hepcidin inhibition in TMPRSS6 mutations associated with iron-refractory iron deficiency anemia | Blood | 2009 |
| 323 | Review | Liu, J; Pu, C; Lang, L; Qiao, L; Abdullahi, M A; Jiang, C | Molecular pathogenesis of hereditary hemochromatosis | Histol Histopathol | 2016 |
| 324 | Unrelated article | Kading, R C; Biggerstaff, B J; Young, G; Komar, N | Mosquitoes used to draw blood for arbovirus viremia determinations in small vertebrates | PLoS One | 2014 |
| 325 | Experimental study | Brewer, C J; Wood, R I; Wood, J C | mRNA regulation of cardiac iron transporters and ferritin subunits in a mouse model of iron overload | Exp Hematol | 2014 |
| 326 | Not HJV gene-related disease | Sussman, N L; Lee, P L; Dries, A M; Schwartz, M R; Barton, J C | Multi-organ iron overload in an African-American man with ALAS2 R452S and SLC40A1 R561G | Acta Haematol | 2008 |
| 327 | Review | Bartnikas, T B; Fleming, M D; Schmidt, P J | Murine mutants in the study of systemic iron metabolism and its disorders: an update on recent advances | Biochim Biophys Acta | 2012 |
| 328 | Not HJV gene-related disease | Roetto, A; Papanikolaou, G; Politou, M; Alberti, F; Girelli, D; Christakis, J; Loukopoulos, D; Camaschella, C | Mutant antimicrobial peptide hepcidin is associated with severe juvenile hemochromatosis | Nat Genet | 2003 |
| 329 | Genetic sequencing not performed | De Gobbi, M; Roetto, A; Piperno, A; Mariani, R; Alberti, F; Papanikolaou, G; Politou, M; Lockitch, G; Girelli, D; Fargion, S; Cox, T M; Gasparini, P; Cazzola, M; Camaschella, C | Natural history of juvenile haemochromatosis | Br J Haematol | 2002 |
| 330 | Experimental study | Zhao, N; Maxson, J E; Zhang, R H; Wahedi, M; Enns, C A; Zhang, A S | Neogenin Facilitates the Induction of Hepcidin Expression by Hemojuvelin in the Liver | J Biol Chem | 2016 |
| 331 | Experimental study | Lee, D H; Zhou, L J; Zhou, Z; Xie, J X; Jung, J U; Liu, Y; Xi, C X; Mei, L; Xiong, W C | Neogenin inhibits HJV secretion and regulates BMP-induced hepcidin expression and iron homeostasis | Blood | 2010 |
| 332 | Experimental study | Yang, F; West, AP Jr; Allendorph, G P; Choe, S; Bjorkman, P J | Neogenin interacts with hemojuvelin through its two membrane-proximal fibronectin type III domains | Biochemistry | 2008 |
| 333 | Experimental study | Enns, C A; Ahmed, R; Zhang, A S | Neogenin interacts with matriptase-2 to facilitate hemojuvelin cleavage | J Biol Chem | 2012 |
| 334 | Experimental study | Zhang, A S; Yang, F; Meyer, K; Hernandez, C; Chapman-Arvedson, T; Bjorkman, P J; Enns, C A | Neogenin-mediated hemojuvelin shedding occurs after hemojuvelin traffics to the plasma membrane | J Biol Chem | 2008 |
| 335 | Not primary hemochromatosis | Bonilla, S; Prozialeck, J D; Malladi, P; Pan, X; Yu, S; Melin-Aldana, H; Whitington, P F | Neonatal iron overload and tissue siderosis due to gestational alloimmune liver disease | J Hepatol | 2012 |
| 336 | Experimental study | Saito, M S; Lourenco, A L; Kang, H C; Rodrigues, C R; Cabral, L M; Castro, H C; Satlher, P C | New approaches in tail-bleeding assay in mice: improving an important method for designing new anti-thrombotic agents | Int J Exp Pathol | 2016 |
| 337 | Review | Roetto, A; Camaschella, C | New insights into iron homeostasis through the study of non-HFE hereditary haemochromatosis | Best Pract Res Clin Haematol | 2005 |
| 338 | Review | Deicher, R; Horl, W H | New insights into the regulation of iron homeostasis | Eur J Clin Invest | 2006 |
| 339 | Not primary hemochromatosis | Malyszko, J; Koc-Zorawska, E; Levin-Iaina, N; Malyszko, J; Kozminski, P; Kobus, G; Mysliwiec, M | New parameters in iron metabolism and functional iron deficiency in patients on maintenance hemodialysis | Pol Arch Med Wewn | 2012 |
| 340 | Experimental study | Koppe, T; Patchen, B; Cheng, A; Bhasin, M; Vulpe, C; Schwartz, R E; Moreno-Navarrete, J M; Fernandez-Real, J M; Pissios, P; Fraenkel, P G | Nicotinamide N-methyltransferase expression decreases in iron overload, exacerbating toxicity in mouse hepatocytes | Hepatol Commun | 2017 |
| 341 | Review | Wallace, D F; Subramaniam, V N | Non-HFE haemochromatosis | World J Gastroenterol | 2007 |
| 342 | Review | Santos, P C; Dinardo, C L; Cancado, R D; Schettert, I T; Krieger, J E; Pereira, A C | Non-HFE hemochromatosis | Rev Bras Hematol Hemoter | 2012 |
| 343 | Review | Pietrangelo, A | Non-HFE hemochromatosis | Semin Liver Dis | 2005 |
| 344 | Review | Pietrangelo, A | Non-HFE hemochromatosis | Hepatology | 2004 |
| 345 | Review | Nelson, J E; Kowdley, K V | Non-HFE hemochromatosis: genetics, pathogenesis, and clinical management | Curr Gastroenterol Rep | 2005 |
| 346 | Review | Bardou-Jacquet, E; Ben, Ali Z; Beaumont-Epinette, M P; Loreal, O; Jouanolle, A M; Brissot, P | Non-HFE hemochromatosis: pathophysiological and diagnostic aspects | Clin Res Hepatol Gastroenterol | 2014 |
| 347 | Review | Pietrangelo, A; Caleffi, A; Corradini, E | Non-HFE hepatic iron overload | Semin Liver Dis | 2011 |
| 348 | Not primary hemochromatosis | Sato, T; Iyama, S; Murase, K; Kamihara, Y; Ono, K; Kikuchi, S; Takada, K; Miyanishi, K; Sato, Y; Takimoto, R; Kobune, M; Kato, J | Novel missense mutation in the TMPRSS6 gene in a Japanese female with iron-refractory iron deficiency anemia | Int J Hematol | 2011 |
| 349 | Review | Kautz, L; Nemeth, E | Novel tools for the evaluation of iron metabolism | Haematologica | 2010 |
| 350 | Not primary hemochromatosis | Visser, M; Davids, M; Verberne, H J; Kok, W E; Tepaske, R; Cocchieri, R; Kemper, E M; Teerlink, T; Jonker, M A; Wisselink, W; de Mol, B A; van Leeuwen, P A | Nutrition before, during, and after surgery increases the arginine:asymmetric dimethylarginine ratio and relates to improved myocardial glucose metabolism: a randomized controlled trial | Am J Clin Nutr | 2014 |
| 351 | Not primary hemochromatosis | Sarafidis, P A; Rumjon, A; Ackland, D; MacLaughlin, H L; Bansal, S S; Brasse-Lagnel, C; Macdougall, I C | Obesity does not influence hepcidin and hemojuvelin levels in hemodialysis patients | Nephron Clin Pract | 2013 |
| 352 | Review | Vaulont, S; Lou, D Q; Viatte, L; Kahn, A | Of mice and men: the iron age | J Clin Invest | 2005 |
| 353 | Review | Brissot, P | Optimizing the diagnosis and the treatment of iron overload diseases | Expert Rev Gastroenterol Hepatol | 2016 |
| 354 | Experimental study | Ishitani, K; Maekawa, K | Ovarian development of female-female pairs in the termite, Reticulitermes speratus | J Insect Sci | 2010 |
| 355 | Review | Zoller, H; Henninger, B | Pathogenesis, Diagnosis and Treatment of Hemochromatosis | Dig Dis | 2016 |
| 356 | Experimental study | Theurl, I; Ludwiczek, S; Eller, P; Seifert, M; Artner, E; Brunner, P; Weiss, G | Pathways for the regulation of body iron homeostasis in response to experimental iron overload | J Hepatol | 2005 |
| 357 | Experimental study | Theurl, I; Schroll, A; Nairz, M; Seifert, M; Theurl, M; Sonnweber, T; Kulaksiz, H; Weiss, G | Pathways for the regulation of hepcidin expression in anemia of chronic disease and iron deficiency anemia in vivo | Haematologica | 2011 |
| 358 | Not primary hemochromatosis | Aigner, E; Theurl, I; Theurl, M; Lederer, D; Haufe, H; Dietze, O; Strasser, M; Datz, C; Weiss, G | Pathways underlying iron accumulation in human nonalcoholic fatty liver disease | Am J Clin Nutr | 2008 |
| 359 | Experimental study | Moriconi, F; Ahmad, G; Ramadori, P; Malik, I; Sheikh, N; Merli, M; Riggio, O; Dudas, J; Ramadori, G | Phagocytosis of gadolinium chloride or zymosan induces simultaneous upregulation of hepcidin- and downregulation of hemojuvelin- and Fpn-1-gene expression in murine liver | Lab Invest | 2009 |
| 360 | Experimental study | Theurl, I; Schroll, A; Sonnweber, T; Nairz, M; Theurl, M; Willenbacher, W; Eller, K; Wolf, D; Seifert, M; Sun, C C; Babitt, J L; Hong, C C; Menhall, T; Gearing, P; Lin, H Y; Weiss, G | Pharmacologic inhibition of hepcidin expression reverses anemia of chronic inflammation in rats | Blood | 2011 |
| 361 | Experimental study | Gnana-Prakasam, J P; Reddy, S K; Veeranan-Karmegam, R; Smith, S B; Martin, P M; Ganapathy, V | Polarized distribution of heme transporters in retinal pigment epithelium and their regulation in the iron-overload disease hemochromatosis | Invest Ophthalmol Vis Sci | 2011 |
| 362 | Experimental study | Maxson, J E; Enns, C A; Zhang, A S | Processing of hemojuvelin requires retrograde trafficking to the Golgi in HepG2 cells | Blood | 2009 |
| 363 | Review | Kawabata, H | Progress in iron metabolism research | Rinsho Ketsueki | 2017 |
| 364 | Experimental study | Kuninger, D; Kuns-Hashimoto, R; Nili, M; Rotwein, P | Pro-protein convertases control the maturation and processing of the iron-regulatory protein, RGMc/hemojuvelin | BMC Biochem | 2008 |
| 365 | Experimental study | Nili, M; David, L; Elferich, J; Shinde, U; Rotwein, P | Proteomic analysis and molecular modelling characterize the iron-regulatory protein haemojuvelin/repulsive guidance molecule c | Biochem J | 2013 |
| 366 | Not HJV gene-related disease | Hamdi-Roze, H; Beaumont-Epinette, M P; Ben, Ali Z; Le Lan, C; Loustaud-Ratti, V; Causse, X; Loreal, O; Deugnier, Y; Brissot, P; Jouanolle, A M; Bardou-Jacquet, E | Rare HFE variants are the most frequent cause of hemochromatosis in non-c282y homozygous patients with hemochromatosis | Am J Hematol | 2016 |
| 367 | Review | Anderson, G J; Frazer, D M | Recent advances in intestinal iron transport | Curr Gastroenterol Rep | 2005 |
| 368 | Review | Robson, K J; Merryweather-Clarke, A T; Cadet, E; Viprakasit, V; Zaahl, M G; Pointon, J J; Weatherall, D J; Rochette, J | Recent advances in understanding haemochromatosis: a transition state | J Med Genet | 2004 |
| 369 | Review | Yan, B C; Hart, J A | Recent developments in liver pathology | Arch Pathol Lab Med | 2009 |
| 370 | Experimental study | Ribeiro, S; Garrido, P; Fernandes, J; Rocha, S; Rocha-Pereira, P; Costa, E; Belo, L; Reis, F; Santos-Silva, A | Recombinant human erythropoietin-induced erythropoiesis regulates hepcidin expression over iron status in the rat | Blood Cells Mol Dis | 2016 |
| 371 | Review | Anderson, G J; Darshan, D; Wilkins, S J; Frazer, D M | Regulation of systemic iron homeostasis: how the body responds to changes in iron demand | Biometals | 2007 |
| 372 | Experimental study | Lakhal, S; Schodel, J; Townsend, A R; Pugh, C W; Ratcliffe, P J; Mole, D R | Regulation of type II transmembrane serine proteinase TMPRSS6 by hypoxia-inducible factors: new link between hypoxia signaling and iron homeostasis | J Biol Chem | 2011 |
| 373 | Unrelated article | Nishiyama, Y; Ito, Y; Shimokata, K; Kimura, Y; Nagata, I | Relationship between establishment of persistent infection of haemagglutinating virus of Japan and the properties of the virus | J Gen Virol | 1976 |
| 374 | Not primary hemochromatosis | Valenti, L; Pulixi, E A; Arosio, P; Cremonesi, L; Biasiotto, G; Dongiovanni, P; Maggioni, M; Fargion, S; Fracanzani, A L | Relative contribution of iron genes, dysmetabolism and hepatitis C virus (HCV) in the pathogenesis of altered iron regulation in HCV chronic hepatitis | Haematologica | 2007 |
| 375 | Experimental study | Constante, M; Wang, D; Raymond, V A; Bilodeau, M; Santos, M M | Repression of repulsive guidance molecule C during inflammation is independent of Hfe and involves tumor necrosis factor-alpha | Am J Pathol | 2007 |
| 376 | Experimental study | Wu, Q; Sun, C C; Lin, H Y; Babitt, J L | Repulsive guidance molecule (RGM) family proteins exhibit differential binding kinetics for bone morphogenetic proteins (BMPs) | PLoS One | 2012 |
| 377 | Experimental study | Healey, E G; Bishop, B; Elegheert, J; Bell, C H; Padilla-Parra, S; Siebold, C | Repulsive guidance molecule is a structural bridge between neogenin and bone morphogenetic protein | Nat Struct Mol Biol | 2015 |
| 378 | Experimental study | Xia, Y; Yu, P B; Sidis, Y; Beppu, H; Bloch, K D; Schneyer, A L; Lin, H Y | Repulsive guidance molecule RGMa alters utilization of bone morphogenetic protein (BMP) type II receptors by BMP2 and BMP4 | J Biol Chem | 2007 |
| 379 | Experimental study | Das, S K; DesAulniers, J; Dyck, J R; Kassiri, Z; Oudit, G Y | Resveratrol mediates therapeutic hepatic effects in acquired and genetic murine models of iron-overload | Liver Int | 2016 |
| 380 | Experimental study | Gnana-Prakasam, J P; Baldowski, R B; Ananth, S; Martin, P M; Smith, S B; Ganapathy, V | Retinal expression of the serine protease matriptase-2 (Tmprss6) and its role in retinal iron homeostasis | Mol Vis | 2014 |
| 381 | Experimental study |  | Retraction: Deficits of learning and memory in Hemojuvelin knockout mice | J Vet Med Sci | 2015 |
| 382 | Unrelated article | von Giesen, H J; Kaiser, R; Koller, H; Wetzel, K; Arendt, G | Reversible ALS-like disorder in HIV infection. An ALS-like syndrome with new HIV infection and complete response to antiretroviral therapy | Neurology | 2002 |
| 383 | Review | Siebold, C; Yamashita, T; Monnier, P P; Mueller, B K; Pasterkamp, R J | RGMs: Structural Insights, Molecular Regulation, and Downstream Signaling | Trends Cell Biol | 2017 |
| 384 | Review | Masson, C | Rheumatoid anemia | Joint Bone Spine | 2011 |
| 385 | Not primary hemochromatosis | Luque-Ramirez, M; Alvarez-Blasco, F; Alpanes, M; Escobar-Morreale, H F | Role of decreased circulating hepcidin concentrations in the iron excess of women with the polycystic ovary syndrome | J Clin Endocrinol Metab | 2011 |
| 386 | Review | Lee, P | Role of matriptase-2 (TMPRSS6) in iron metabolism | Acta Haematol | 2009 |
| 387 | Experimental study | Bogdan, M; Silosi, I; Surlin, P; Tica, A A; Tica, O S; Balseanu, T A; Rauten, A M; Camen, A | Salivary and serum biomarkers for the study of side effects of aripiprazole coprescribed with mirtazapine in rats | Int J Clin Exp Med | 2015 |
| 388 | Review | Stryker, J A | Science to practice: why is the liver a radiosensitive organ? | Radiology | 2007 |
| 389 | Not HJV gene-related disease | Roetto, A; Daraio, F; Porporato, P; Caruso, R; Cox, T M; Cazzola, M; Gasparini, P; Piperno, A; Camaschella, C | Screening hepcidin for mutations in juvenile hemochromatosis: identification of a new mutation (C70R) | Blood | 2004 |
| 390 | Experimental study | Kuns-Hashimoto, R; Kuninger, D; Nili, M; Rotwein, P | Selective binding of RGMc/hemojuvelin, a key protein in systemic iron metabolism, to BMP-2 and neogenin | Am J Physiol Cell Physiol | 2008 |
| 391 | Experimental study | Turato, C; Kent, P; Sebastiani, G; Cannito, S; Morello, E; Terrin, L; Biasiolo, A; Simonato, D; Parola, M; Pantopoulos, K; Pontisso, P | Serpinb3 is overexpressed in the liver in presence of iron overload | J Investig Med | 2018 |
| 392 | Not primary hemochromatosis | Rumjon, A; Sarafidis, P; Brincat, S; Musto, R; Malyszko, J; Bansal, S S; Macdougall, I C | Serum hemojuvelin and hepcidin levels in chronic kidney disease | Am J Nephrol | 2012 |
| 393 | Not primary hemochromatosis | Robach, P; Recalcati, S; Girelli, D; Campostrini, N; Kempf, T; Wollert, K C; Corbella, M; Santambrogio, P; Perbellini, L; Brasse-Lagnel, C; Christensen, B; Moutereau, S; Lundby, C; Cairo, G | Serum hepcidin levels and muscle iron proteins in humans injected with low- or high-dose erythropoietin | Eur J Haematol | 2013 |
| 394 | Not HJV gene-related disease | Matthes, T; Aguilar-Martinez, P; Pizzi-Bosman, L; Darbellay, R; Rubbia-Brandt, L; Giostra, E; Michel, M; Ganz, T; Beris, P | Severe hemochromatosis in a Portuguese family associated with a new mutation in the 5'-UTR of the HAMP gene | Blood | 2004 |
| 395 | Not primary hemochromatosis | Lee, P; Rice, L; McCarthy, J J; Beutler, E | Severe iron overload with a novel aminolevulinate synthase mutation and hepatitis C infection. A case report | Blood Cells Mol Dis | 2009 |
| 396 | Experimental study | Lee, P; Hsu, M H; Welser-Alves, J; Peng, H | Severe microcytic anemia but increased erythropoiesis in mice lacking Hfe or Tfr2 and Tmprss6 | Blood Cells Mol Dis | 2012 |
| 397 | Not HJV gene-related disease | Le Lan, C; Mosser, A; Ropert, M; Detivaud, L; Loustaud-Ratti, V; Vital-Durand, D; Roget, L; Bardou-Jacquet, E; Turlin, B; David, V; Loreal, O; Deugnier, Y; Brissot, P; Jouanolle, A M | Sex and acquired cofactors determine phenotypes of ferroportin disease | Gastroenterology | 2011 |
| 398 | Experimental study | Brewer, C; Otto-Duessel, M; Wood, R I; Wood, J C | Sex differences and steroid modulation of cardiac iron in a mouse model of iron overload | Transl Res | 2014 |
| 399 | Experimental study | Chen, W; Huang, F W; de Renshaw, T B; Andrews, N C | Skeletal muscle hemojuvelin is dispensable for systemic iron homeostasis | Blood | 2011 |
| 400 | Experimental study | Jenkitkasemwong, S; Wang, C Y; Coffey, R; Zhang, W; Chan, A; Biel, T; Kim, J S; Hojyo, S; Fukada, T; Knutson, M D | SLC39A14 Is Required for the Development of Hepatocellular Iron Overload in Murine Models of Hereditary Hemochromatosis | Cell Metab | 2015 |
| 401 | Not HJV gene-related disease | Lee, P L; Gelbart, T; West, C; Barton, J C | SLC40A1 c.1402G-->a results in aberrant splicing, ferroportin truncation after glycine 330, and an autosomal dominant hemochromatosis phenotype | Acta Haematol | 2007 |
| 402 | Experimental study | Vujic, Spasic M; Sparla, R; Mleczko-Sanecka, K; Migas, M C; Breitkopf-Heinlein, K; Dooley, S; Vaulont, S; Fleming, R E; Muckenthaler, M U | Smad6 and Smad7 are co-regulated with hepcidin in mouse models of iron overload | Biochim Biophys Acta | 2013 |
| 403 | Experimental study | Mleczko-Sanecka, K; Casanovas, G; Ragab, A; Breitkopf, K; Muller, A; Boutros, M; Dooley, S; Hentze, M W; Muckenthaler, M U | SMAD7 controls iron metabolism as a potent inhibitor of hepcidin expression | Blood | 2010 |
| 404 | Not primary hemochromatosis | Ferro, E; Di Pietro, A; Visalli, G; Piraino, B; Salpietro, C; La Rosa, M A | Soluble hemojuvelin in transfused and untransfused thalassaemic subjects | Eur J Haematol | 2017 |
| 405 | Experimental study | Lin, L; Nemeth, E; Goodnough, J B; Thapa, D R; Gabayan, V; Ganz, T | Soluble hemojuvelin is released by proprotein convertase-mediated cleavage at a conserved polybasic RNRR site | Blood Cells Mol Dis | 2008 |
| 406 | Experimental study | Nili, M; Shinde, U; Rotwein, P | Soluble repulsive guidance molecule c/hemojuvelin is a broad spectrum bone morphogenetic protein (BMP) antagonist and inhibits both BMP2- and BMP6-mediated signaling and gene expression | J Biol Chem | 2010 |
| 407 | Review | Gutierrez, O M; Sun, C C; Chen, W; Babitt, J L; Lin, H Y | Statement of concern about a commercial assay used to measure soluble hemojuvelin in humans | Am J Nephrol | 2012 |
| 408 | Experimental study | Carlson, M R; Gray, D L; Richers, C P; Wang, W; Zhao, P H; Rauchfuss, T B; Pelmenschikov, V; Pham, C C; Gee, L B; Wang, H; Cramer, S P | Sterically Stabilized Terminal Hydride of a Diiron Dithiolate | Inorg Chem | 2018 |
| 409 | Experimental study | Wysocka, M; Gruba, N; Miecznikowska, A; Popow-Stellmaszyk, J; Gutschow, M; Stirnberg, M; Furtmann, N; Bajorath, J; Lesner, A; Rolka, K | Substrate specificity of human matriptase-2 | Biochimie | 2014 |
| 410 | Experimental study | Truksa, J; Gelbart, T; Peng, H; Beutler, E; Beutler, B; Lee, P | Suppression of the hepcidin-encoding gene Hamp permits iron overload in mice lacking both hemojuvelin and matriptase-2/TMPRSS6 | Br J Haematol | 2009 |
| 411 | Experimental study | Tsuchiya, H; Akechi, Y; Ikeda, R; Nishio, R; Sakabe, T; Terabayashi, K; Matsumi, Y; Ashla, A A; Hoshikawa, Y; Kurimasa, A; Suzuki, T; Ishibashi, N; Yanagida, S; Shiota, G | Suppressive effects of retinoids on iron-induced oxidative stress in the liver | Gastroenterology | 2009 |
| 412 | Experimental study | Li, N; Chen, Q; Gu, J; Li, S; Zhao, G; Wang, W; Wang, Z; Wang, X | Synergistic inhibitory effects of deferasirox in combination with decitabine on leukemia cell lines SKM-1, THP-1, and K-562 | Oncotarget | 2017 |
| 413 | Review | Steele, T M; Frazer, D M; Anderson, G J | Systemic regulation of intestinal iron absorption | IUBMB Life | 2005 |
| 414 | Review | Sun, C C; Vaja, V; Babitt, J L; Lin, H Y | Targeting the hepcidin-ferroportin axis to develop new treatment strategies for anemia of chronic disease and anemia of inflammation | Am J Hematol | 2012 |
| 415 | Unrelated article | Kimura, Y; Ito, Y; Shimokata, K; Nishiyama, Y; Nagata, I | Temperature-sensitive virus derived from BHK cells persistently infected with HVJ (Sendai virus) | J Virol | 1975 |
| 416 | Unrelated article | Barton, B E; Rauchfuss, T B | Terminal hydride in [FeFe]-hydrogenase model has lower potential for H2 production than the isomeric bridging hydride | Inorg Chem | 2008 |
| 417 | Experimental study | Wang, L; Trebicka, E; Fu, Y; Ellenbogen, S; Hong, C C; Babitt, J L; Lin, H Y; Cherayil, B J | The bone morphogenetic protein-hepcidin axis as a therapeutic target in inflammatory bowel disease | Inflamm Bowel Dis | 2012 |
| 418 | Review | Brissot, P; Troadec, M B; Loreal, O | The clinical relevance of new insights in iron transport and metabolism | Curr Hematol Rep | 2004 |
| 419 | Review | Aguilar-Martinez, P; Schved, J F; Brissot, P | The evaluation of hyperferritinemia: an updated strategy based on advances in detecting genetic abnormalities | Am J Gastroenterol | 2005 |
| 420 | Not HJV gene-related disease | Camaschella, C; Roetto, A; Cali, A; De Gobbi, M; Garozzo, G; Carella, M; Majorano, N; Totaro, A; Gasparini, P | The gene TFR2 is mutated in a new type of haemochromatosis mapping to 7q22 | Nat Genet | 2000 |
| 421 | Review | Wallace, D F; Subramaniam, V N | The global prevalence of HFE and non-HFE hemochromatosis estimated from analysis of next-generation sequencing data | Genet Med | 2016 |
| 422 | Experimental study | D'Alessio, F; Hentze, M W; Muckenthaler, M U | The hemochromatosis proteins HFE, TfR2, and HJV form a membrane-associated protein complex for hepcidin regulation | J Hepatol | 2012 |
| 423 | Experimental study | Lee, P; Peng, H; Gelbart, T; Beutler, E | The IL-6- and lipopolysaccharide-induced transcription of hepcidin in HFE-, transferrin receptor 2-, and beta 2-microglobulin-deficient hepatocytes | Proc Natl Acad Sci U S A | 2004 |
| 424 | Not HJV gene-related disease | Barbara, K H; Marcin, L; Jedrzej, A; Wieslaw, Z; Elzbieta, A D; Malgorzata, M; Ewa, M; Jacek, K J | The impact of H63D HFE gene carriage on hemoglobin and iron status in children | Ann Hematol | 2016 |
| 425 | Review | Deugnier, Y | The iron driven pathway of hepcidin synthesis | Gastroenterol Clin Biol | 2010 |
| 426 | Experimental study | Simonis, G; Mueller, K; Schwarz, P; Wiedemann, S; Adler, G; Strasser, R H; Kulaksiz, H | The iron-regulatory peptide hepcidin is upregulated in the ischemic and in the remote myocardium after myocardial infarction | Peptides | 2010 |
| 427 | Experimental study | Castoldi, M; Vujic, Spasic M; Altamura, S; Elmen, J; Lindow, M; Kiss, J; Stolte, J; Sparla, R; D'Alessandro, L A; Klingmuller, U; Fleming, R E; Longerich, T; Grone, H J; Benes, V; Kauppinen, S; Hentze, M W; Muckenthaler, M U | The liver-specific microRNA miR-122 controls systemic iron homeostasis in mice | J Clin Invest | 2011 |
| 428 | Review | Kawabata, H | The mechanisms of systemic iron homeostasis and etiology, diagnosis, and treatment of hereditary hemochromatosis | Int J Hematol | 2018 |
| 429 | Experimental study | Mok, H; Mlodnicka, A E; Hentze, M W; Muckenthaler, M; Schumacher, A | The molecular circuitry regulating the switch between iron deficiency and overload in mice | J Biol Chem | 2006 |
| 430 | Review | Le Gac, G; Ferec, C | The molecular genetics of haemochromatosis | Eur J Hum Genet | 2005 |
| 431 | Review | Babitt, J L; Lin, H Y | The molecular pathogenesis of hereditary hemochromatosis | Semin Liver Dis | 2011 |
| 432 | Review | Chua, A C; Graham, R M; Trinder, D; Olynyk, J K | The regulation of cellular iron metabolism | Crit Rev Clin Lab Sci | 2007 |
| 433 | Not primary hemochromatosis | Boga, S; Alkim, H; Alkim, C; Koksal, A R; Bayram, M; Yilmaz, Ozguven MB; Tekin, Neijmann S | The Relationship of Serum Hemojuvelin and Hepcidin Levels with Iron Overload in Nonalcoholic Fatty Liver Disease | J Gastrointestin Liver Dis | 2015 |
| 434 | Review | Oates, P S | The relevance of the intestinal crypt and enterocyte in regulating iron absorption | Pflugers Arch | 2007 |
| 435 | Review | Coimbra, S; Catarino, C; Santos-Silva, A | The role of adipocytes in the modulation of iron metabolism in obesity | Obes Rev | 2013 |
| 436 | Experimental study | Zhang, A S; Gao, J; Koeberl, D D; Enns, C A | The role of hepatocyte hemojuvelin in the regulation of bone morphogenic protein-6 and hepcidin expression in vivo | J Biol Chem | 2010 |
| 437 | Not primary hemochromatosis | Przybyszewska, J; Zekanowska, E | The role of hepcidin and haemojuvelin in the pathogenesis of iron disorders in patients with severe malnutrition | Ann Agric Environ Med | 2014 |
| 438 | Experimental study | Dokuyucu, R; Demir, T; Yumrutas, Onder; Erbagci, A B; Orkmez, M; Bahar, A Y; Bayraktar, R; Bozgeyik, I; Kaplan, D S; Cengiz, B; Bagci, B C | The role of hepcidin and its related genes (BMP6, GDF-15, and HJV) in rats exposed to ischemia and reperfusion | Turk J Med Sci | 2014 |
| 439 | Experimental study | Silvestri, L; Pagani, A; Nai, A; De Domenico, I; Kaplan, J; Camaschella, C | The serine protease matriptase-2 (TMPRSS6) inhibits hepcidin activation by cleaving membrane hemojuvelin | Cell Metab | 2008 |
| 440 | Unrelated article | Liwang, A C; Wang, Z X; Sun, Y; Peiper, S C; Liwang, P J | The solution structure of the anti-HIV chemokine vMIP-II | Protein Sci | 1999 |
| 441 | Experimental study | Nai, A; Pagani, A; Silvestri, L; Campostrini, N; Corbella, M; Girelli, D; Traglia, M; Toniolo, D; Camaschella, C | TMPRSS6 rs855791 modulates hepcidin transcription in vitro and serum hepcidin levels in normal individuals | Blood | 2011 |
| 442 | Experimental study | Dion, S P; Beliveau, F; Desilets, A; Ghinet, M G; Leduc, R | Transcriptome analysis reveals TMPRSS6 isoforms with distinct functionalities | J Cell Mol Med | 2018 |
| 443 | Experimental study | Troadec, M B; Fautrel, A; Drenou, B; Leroyer, P; Camberlein, E; Turlin, B; Guillouzo, A; Brissot, P; Loreal, O | Transcripts of ceruloplasmin but not hepcidin, both major iron metabolism genes, exhibit a decreasing pattern along the portocentral axis of mouse liver | Biochim Biophys Acta | 2008 |
| 444 | Experimental study | Itani, T; Kobayashi, Y; Kuroda, M; Ma, N; Mifuji, R; Urawa, N; Tanaka, Y; Kaito, M; Adachi, Y | Transfecting the multidrug resistance protein 2 gene improves transcellular organic anion transport | Int J Mol Med | 2005 |
| 445 | Experimental study | Rapisarda, C; Puppi, J; Hughes, R D; Dhawan, A; Farnaud, S; Evans, R W; Sharp, P A | Transferrin receptor 2 is crucial for iron sensing in human hepatocytes | Am J Physiol Gastrointest Liver Physiol | 2010 |
| 446 | Not HJV gene-related disease | Ravasi, G; Rausa, M; Pelucchi, S; Arosio, C; Greni, F; Mariani, R; Pelloni, I; Silvestri, L; Pineda, P; Camaschella, C; Piperno, A | Transferrin receptor 2 mutations in patients with juvenile hemochromatosis phenotype | Am J Hematol | 2015 |
| 447 | Experimental study | Chen, S; Feng, T; Vujic, Spasic M; Altamura, S; Breitkopf-Heinlein, K; Altenoder, J; Weiss, T S; Dooley, S; Muckenthaler, M U | Transforming Growth Factor beta1 (TGF-beta1) Activates Hepcidin mRNA Expression in Hepatocytes | J Biol Chem | 2016 |
| 448 | Experimental study | Kupatt, C; Habazettl, H; Goedecke, A; Wolf, D A; Zahler, S; Boekstegers, P; Kelly, R A; Becker, B F | Tumor necrosis factor-alpha contributes to ischemia- and reperfusion-induced endothelial activation in isolated hearts | Circ Res | 1999 |
| 449 | Experimental study | Salama, M F; Bayele, H K; Srai, S S | Tumour necrosis factor alpha downregulates human hemojuvelin expression via a novel response element within its promoter | J Biomed Sci | 2012 |
| 450 | Experimental study | Truksa, J; Lee, P; Beutler, E | Two BMP responsive elements, STAT, and bZIP/HNF4/COUP motifs of the hepcidin promoter are critical for BMP, SMAD1, and HJV responsiveness | Blood | 2009 |
| 451 | Not primary hemochromatosis | Slavin, T P; Feng, T; Schnell, A; Zhu, X; Elston, R C | Two-marker association tests yield new disease associations for coronary artery disease and hypertension | Hum Genet | 2011 |
| 452 | Review | Yun, S; Vincelette, N D | Update on iron metabolism and molecular perspective of common genetic and acquired disorder, hemochromatosis | Crit Rev Oncol Hematol | 2015 |
| 453 | Not primary hemochromatosis | Wang, J J; Chi, N H; Huang, T M; Connolly, R; Chen, L W; Chueh, S J; Kan, W C; Lai, C C; Wu, V C; Fang, J T; Chu, T S; Wu, K D | Urinary biomarkers predict advanced acute kidney injury after cardiovascular surgery | Crit Care | 2018 |
| 454 | Experimental study | Mendes, J F; Siqueira, E M; de Brito, E Silva JG; Arruda, S F | Vitamin A deficiency modulates iron metabolism independent of hemojuvelin (Hfe2) and bone morphogenetic protein 6 (Bmp6) transcript levels | Genes Nutr | 2016 |
| 455 | Experimental study | Christiansen, H; Sheikh, N; Saile, B; Reuter, F; Rave-Frank, M; Hermann, R M; Dudas, J; Hille, A; Hess, C F; Ramadori, G | x-Irradiation in rat liver: consequent upregulation of hepcidin and downregulation of hemojuvelin and ferroportin-1 gene expression | Radiology | 2007 |
| 456 | Experimental study | De Domenico, I; Vaughn, M B; Yoon, D; Kushner, J P; Ward, D M; Kaplan, J | Zebrafish as a model for defining the functional impact of mammalian ferroportin mutations | Blood | 2007 |
| 457 | Experimental study | Wu, J; Yang, L; Zhang, X; Li, Y; Wang, J; Zhang, S; Liu, H; Huang, H; Wang, Y; Yuan, L; Cheng, X; Zhuang, D; Zhang, H; Chen, X | MC-LR induces dysregulation of iron homeostasis by inhibiting hepcidin expression: A preliminary study | Chemosphere | 2018 |
| 458 | Experimental study | Bogdan M, Silosi I, Surlin P, Tica AA, Tica OS, Balseanu TA, Rauten AM, Cioloca D, Camen A | Liver Fatty Acid Binding Protein And Hemojuvelin - Potential Biomarkers For Liver Function in Rat Model. | Curr Health Sci J | 2015 |
| 459 | Experimental study | Fillebeen, C; Wilkinson, N; Charlebois, E; Katsarou, A; Wagner, J; Pantopoulos, K | Hepcidin-mediated hypoferremic response to acute inflammation requires a threshold of Bmp6/Hjv/Smad signaling | Blood | 2018 |
| 460 | Not primary hemochromatosis | El SH, Abou SK, Ahmed YS, Abou EH, El ST, Behairy MA, Mohamed MM, Ahmed FA | Relationship of serum haemojuvelin and hepcidin levels with iron level and erythropoietin requirement in prevalent hepatitis C virus positive haemodialysis patients | Nephrology (Carlton) | 2018 |
| 461 | Review | Pantopoulo K | Inherited Disorders of Iron Overload | Frontiers in Nutrition | 2018 |
| 462 | Not HJV gene-related disease | Zhang, W.; Xu, A.; Li, Y.; Zhao, S.; Zhou, D.; Wu, L.; Zhang, B.; Zhao, X.; Wang, Y.; Wang, X.; Duan, W.; Wang, Q.; Nan, Y.; You, H.; Jia, J.; Ou, X.; Huang, J. | A novel SLC40A1 p.Y333H mutation with gain of function of ferroportin: A recurrent cause of haemochromatosis in China | Liver Int | 2018 |
| 463 | Experimental study | Mao, P.; Wortham, A. M.; Enns, C. A.; Zhang, A. S. | The catalytic, stem, and transmembrane portions of matriptase-2 are required for suppressing the expression of the iron-regulatory hormone hepcidin | J Biol Chem | 2018 |
| 464 | Unrelated article | Masajtis-Zagajewska, A.; Nowicki, M | Effect of atorvastatin on iron metabolism regulation in patients with chronic kidney disease - a randomized double blind crossover study | Ren Fail | 2018 |
| 465 | Not HJV gene-related disease | Barton, J C; Rivers, C A; Niyongere, S; Bohannon, S B; Acton, R T | Allele frequencies of hemojuvelin gene (HJV) I222N and G320V missense mutations in white and African American subjects from the general Alabama population | BMC Med Genet | 2004 |

1. **Articles excluded after full-text reviewed with reasons n=24**

| **ID** | **Exclusion reason** | **Author** | **Title** | **Journal** | **Year** |
| --- | --- | --- | --- | --- | --- |
| 1 | Without phenotype description | Laursen, A H; Bjerrum, O W; Friis-Hansen, L; Hansen, T O; Marott, J L; Magnussen, K | Causes of iron overload in blood donors - a clinical study | Vox Sang | 2018 |
| 2 | Without phenotype description | Barton, J C; Acton, R T; Leiendecker-Foster, C; Lovato, L; Adams, P C; Eckfeldt, J H; McLaren, C E; Reiss, J A; McLaren, G D; Reboussin, D M; Gordeuk, V R; Speechley, M R; Press, R D; Dawkins, F W | Characteristics of participants with self-reported hemochromatosis or iron overload at HEIRS study initial screening | Am J Hematol | 2008 |
| 3 | Without phenotype description | Hayashi, H; Wakusawa, S; Yano, M; Okada, T | Genetic background of Japanese patients with adult-onset storage diseases in the liver | Hepatol Res | 2007 |
| 4 | Review | Hayashi, H; Wakusawa, S; Motonishi, S; Miyamoto, K; Okada, H; Inagaki, Y; Ikeda, T | Genetic background of primary iron overload syndromes in Japan | Intern Med | 2006 |
| 5 | Not HJV gene-related disease | Barton, J C; Acton, R T; Leiendecker-Foster, C; Lovato, L; Adams, P C; McLaren, G D; Eckfeldt, J H; McLaren, C E; Reboussin, D M; Gordeuk, V R; Speechley, M R; Reiss, J A; Press, R D; Dawkins, F W | HFE C282Y homozygotes aged 25-29 years at HEIRS Study initial screening | Genet Test | 2007 |
| 6 | Without phenotype description | Barton, J C; Lafreniere, S A; Leiendecker-Foster, C; Li, H; Acton, R T; Press, R D; Eckfeldt, J H | HFE, SLC40A1, HAMP, HJV, TFR2, and FTL mutations detected by denaturing high-performance liquid chromatography after iron phenotyping and HFE C282Y and H63D genotyping in 785 HEIRS Study participants | Am J Hematol | 2009 |
| 7 | Not HJV gene-related disease | Badar, S; Busti, F; Ferrarini, A; Xumerle, L; Bozzini, P; Capelli, P; Pozzi-Mucelli, R; Campostrini, N; De Matteis, G; Marin, Vargas S; Giorgetti, A; Delledonne, M; Olivieri, O; Girelli, D | Identification of novel mutations in hemochromatosis genes by targeted next generation sequencing in Italian patients with unexplained iron overload | Am J Hematol | 2016 |
| 8 | Not HJV gene-related disease | Barton, J C; Lee, P L; West, C; Bottomley, S S | Iron overload and prolonged ingestion of iron supplements: clinical features and mutation analysis of hemochromatosis-associated genes in four cases | Am J Hematol | 2006 |
| 9 | Genetic sequencing not performed | Unlusoy, Aksu A; Caleffi, A; Pietrangelo, A; Sari, S; Egritas, Gurkan O; Demirtas, Z; Yilmaz, G; Dalgic, B | Iron Overload in the Liver of 2 Children: Nonalcoholic Steatohepatitis and Juvenile Hemochromatosis | J Pediatr Hematol Oncol | 2017 |
| 10 | Genetic sequencing not performed | De Gobbi, M; Pasquero, P; Brunello, F; Paccotti, P; Mazza, U; Camaschella, C | Juvenile hemochromatosis associated with B-thalassemia treated by phlebotomy and recombinant human erythropoietin | Haematologica | 2000 |
| 11 | Not HJV gene-related disease | Pietrangelo, A; Caleffi, A; Henrion, J; Ferrara, F; Corradini, E; Kulaksiz, H; Stremmel, W; Andreone, P; Garuti, C | Juvenile hemochromatosis associated with pathogenic mutations of adult hemochromatosis genes | Gastroenterology | 2005 |
| 12 | Genetic sequencing not performed | Nobakht, H; Zolfaghari, S; Pourazizi, M; Malek, M | Juvenile Hemochromatosis in Iran: A Case Report with 5-Year Follow-up after Treatment | Middle East J Dig Dis | 2016 |
| 13 | Genetic sequencing not performed | Cazes, A; Duong, Van Huyen JP; Fornes, P; Amrein, C; Guillemain, R; Grinda, J M; Bruneval, P | Mechanical ventricular assistance in heart failure: pathology of the cardiac apex removed during device implantation | Cardiovasc Pathol | 2010 |
| 14 | Genetic sequencing not performed | Wouthuis, S F; van Deursen, C T; Te, Lintelo MP; Rozeman, C A; Beekman, R | Neuromuscular manifestations in hereditary haemochromatosis | J Neurol | 2010 |
| 15 | Without phenotype description | Faria, R; Silva, B; Silva, C; Loureiro, P; Queiroz, A; Fraga, S; Esteves, J; Mendes, D; Fleming, R; Vieira, L; Goncalves, J; Faustino, P | Next-generation sequencing of hereditary hemochromatosis-related genes: Novel likely pathogenic variants found in the Portuguese population | Blood Cells Mol Dis | 2016 |
| 16 | Not HJV gene-related disease | Mendes, A I; Ferro, A; Martins, R; Picanco, I; Gomes, S; Cerqueira, R; Correia, M; Nunes, A R; Esteves, J; Fleming, R; Faustino, P | Non-classical hereditary hemochromatosis in Portugal: novel mutations identified in iron metabolism-related genes | Ann Hematol | 2009 |
| 17 | Review | Sandhu, K; Flintoff, K; Chatfield, M D; Dixon, J L; Ramm, L E; Ramm, G A; Powell, L W; Subramaniam, V N; Wallace, D F | Phenotypic analysis of hemochromatosis subtypes reveals variations in severity of iron overload and clinical disease | Blood | 2018 |
| 18 | Without phenotype description | Pissia, M; Polonifi, K; Politou, M; Lilakos, K; Sakellaropoulos, N; Papanikolaou, G | Prevalence of the G320V mutation of the HJV gene, associated with juvenile hemochromatosis, in Greece | Haematologica | 2004 |
| 19 | Not HJV gene-related disease | Fabio, G; Minonzio, F; Delbini, P; Bianchi, A; Cappellini, M D | Reversal of cardiac complications by deferiprone and deferoxamine combination therapy in a patient affected by a severe type of juvenile hemochromatosis (JH) | Blood | 2007 |
| 20 | Genetic sequencing not performed | Blank, R; Wolber, T; Maeder, M; Rickli, H | Reversible cardiomyopathy in a patient with juvenile hemochromatosis | Int J Cardiol | 2006 |
| 21 | Not HJV gene-related disease | Funakoshi, N; Chaze, I; Alary, A S; Tachon, G; Cunat, S; Giansily-Blaizot, M; Bismuth, M; Larrey, D; Pageaux, G P; Schved, J F; Donnadieu-Rigole, H; Blanc, P; Aguilar-Martinez, P | The role of genetic factors in patients with hepatocellular carcinoma and iron overload - a prospective series of 234 patients | Liver Int | 2016 |
| 22 | Genetic sequencing not performed | Mantilla-Hernández, Julio C.; Amaya-Mujica, Julián | Juvenile hemochromatosis with multi-organ involvement diagnosed at autopsy | Revista Española de Patología | 2019 |
| 23 | Review | Silvestri, L; Nai, A; Dulja, A; Pagani, A | Hepcidin and the BMP-SMAD pathway: An unexpected liaison | Vitam Horm | 2019 |
| 24 | Review | Rotwein, P | Variation in the repulsive guidance molecule family in human populations | Physiol Rep | 2019 |
