# Supplementary material for: Genotypic and phenotypic spectra of hemojuvelin mutations in primary hemochromatosis patients: a systematic review
Source: Orphanet J Rare Dis. 2019 Jul 8;14:171. doi: 10.1186/s13023-019-1097-2 (PMC6615163; doi:10.1186/s13023-019-1097-2)
Supplement: Supplementary file 3 — Clinical findings for cases with biallelic mutations. (DOCX 96 kb) [file 13023_2019_1097_MOESM3_ESM.docx]

**Additional file 3.** Clinical findings for cases with biallelic mutations.

| **ID** | **Amino acid change (HGVS nomenclature)** | | **Nucleotide change (HGVS nomenclature)** | | **Sex** | **Age at diagnosis** | **Age at presentation** | **Proband** | **SF (ng/ml)** | **TS (%)** | **Other gene variants** | **Family origin** | **Cardiomyopathy** | **Skin hyperpigmentation** | **Arthropathy** | **Endocrine abnormality** | | | | **Liver abnormality** | | | | **Liver biopsy** | **Treatments** | **Outcomes** | **Reference** |
| --- | --- | --- | --- | --- | --- | --- | --- | --- | --- | --- | --- | --- | --- | --- | --- | --- | --- | --- | --- | --- | --- | --- | --- | --- | --- | --- | --- |
|  | **Allele1** | **Allele2** | **Allele1** | **Allele2** |  |  |  |  |  |  |  |  |  |  |  | **Hypogonadism** | **Glucose intolerance** | **Osteopathy** | **Thyroid abnormality** | **Abnormal liver function test** | **Liver iron deposition** | **Liver fibrosis** | **Liver cirrhosis** |  |  |  |  |
| **Caucasians** | | | | | | | | | | | | | | | | | | | | | | | | | | | |
| 1 | p.Leu28SerfsTer24 (p.L28fs) | p.Leu28SerfsTer24 (p.L28fs) | c.81delG | c.81delG | Female | 25 | 25 | Yes | >500 | — |  | England/Ireland | Yes | No | — | Yes | No | No | — | Yes | Yes | Yes | No | Yes | Phlebotomy | Achieved partial iron depletion and normal heart function test. | 1 |
| 2 | p.Arg41Pro (p.R41P) | p.Arg176Cys (p.R176C) | c.122G>C | c.526C>T | Male | 53 | — | Yes | 4220 | 86 |  | France | — | Yes | Yes | — | — | — | — | — | Yes | — | Yes | Yes | Phlebotomy | — | 2 |
| 3 | p.Gly66Ter (p.G66*) | p.Gly66Ter (p.G66*) | c.196G>T | c.196G>T | Male | 25 | 25 | Yes | 4220 | 94 |  | Czech | No | — | — | No | No | — | — | Yes | Yes | Yes | — | Yes | Phlebotomy | — | 3 |
| 4 | p.Gly66Ter (p.G66*) | p.Gly66Ter (p.G66*) | c.196G>T | c.196G>T | Male | 25 | 25 | Yes | 21857 | 94 |  | Romania | Yes | Yes | Yes | Yes | Yes | — | — | Yes | Yes | Yes | No | Yes | Died before therapy | Died after diagnosis because of circulatory status deterioration. | 4 |
| 5 | p.Val74TrpfsTer40 (p.V74fs) | p.Asn269LysfsTer43 (p.N269fs) | c.220delG | c.806dupA | Female | 24 | 24 | Yes | 2130 | 100 |  | England | No | No | Yes | Yes | No | — | — | — | Yes | — | Yes | Yes | — | — | 5 |
| 6 | p.Cys80Arg (p.C80R) | p.Leu101Pro (p.L101P) | c.238T>C | c.302T>C | Female | 23 | 18 | Yes | — | 96 |  | Southeast United States | No | Yes | Yes | Yes | — | — | — | — | — | Yes | No | Yes | Phlebotomy, sex hormone replacement | Achieved iron depletion and maintained by periodic phlebotomy, resolution of hyperpigmentation and regression of hepatomegaly. | 6 |
| 7 | p.Cys80Arg (p.C80R) | p.Leu101Pro (p.L101P) | c.238T>C | c.302T>C | Female | 21 | 17 | No | — | 98 |  | Southeast United States | No | Yes | — | Yes | — | — | — | — | — | Yes | No | Yes | Phlebotomy, sex hormone replacement | Achieved iron depletion and maintained by periodic phlebotomy, resolution of hyperpigmentation and regression of hepatomegaly. | 6 |
| 8 | p.Cys80Arg (p.C80R) | p.Arg326Ter (p.R326*) | c.238T>C | c.976C>T | Female | 18 | 18 | Yes | 2600 | 91 |  | Australia | — | — | Yes | — | — | — | — | Yes | Yes | Yes | — | Yes | — | — | 7 |
| 9 | p.Cys80Try (p.C80Y) | p.Gly320Val (p.G320V) | c.239G>A | c.959G>T | Female | 18 | 18 | Yes | 5293 | 100 |  | Bangladesh/United Kingdom | — | — | — | Yes | Yes | — | — | — | — | — | — | No | — | — | 8 |
| 10 | p.Ser85Pro (p.S85P) | p.Ser85Pro (p.S85P) | c.253T>C | c.253T>C | Female | 30 | 30 | Yes | 823 | 100 |  | Italy | No | — | — | Yes | — | — | — | — | — | Yes | — | Yes | — | — | 5 |
| 11 | p.Cys89Arg (p.C89R) | p.Cys89Arg (p.C89R) | c.265T>C | c.265T>C | Male | 24 | 24 | Yes | >2000 | 85 | *HFE*: V285A/V285V | Western Iran (Kurd and Lur) | Yes | Yes | — | Yes | — | — | — | Yes | Yes | Yes | — | — | Phlebotomy | Achieved iron depletion and normal liver function test. | 9 |
| 12 | p.Cys89Arg (p.C89R) | p.Cys89Arg (p.C89R) | c.265T>C | c.265T>C | Female | 30 | — | No | 4350 | 73 |  | Western Iran (Kurd and Lur) | — | — | — | — | — | — | — | Yes | — | Yes | — | — | Phlebotomy | Achieved partial iron depletion and normal liver function test. | 9 |
| 13 | p.Gly99Arg (p.G99R) | p.Gly99Arg (p.G99R) | c.295G>A | c.295G>A | Male | 26 | 26 | Yes | 4485 | — |  | Pakistan | — | — | — | Yes | Yes | Yes | — | — | Yes | — | Yes | Yes | — | — | 8 |
| 14 | p.Gly99Arg (p.G99R) | p.Gly99Arg (p.G99R) | c.295G>A | c.295G>A | Female | 11 | 11 | Yes | 3772 | — |  | Pakistan | — | — | — | — | — | — | — | — | Yes | — | — | Yes | — | — | 8 |
| 15 | p.Gly99Val (p.G99V) | p.Gly99Val (p.G99V) | c.296G>T | c.296G>T | Male | 33 | 28 | Yes | 1125 | 80 |  | Greece | No | No | Yes | Yes | Yes | — | — | — | — | Yes | Yes | Yes | Phlebotomy | — | 10 |
| 16 | p.Gly99Val (p.G99V) | p.Gly99Val (p.G99V) | c.296G>T | c.296G>T | Female | 40 | 34 | No | 1229 | 60 |  | Greece | Yes | Yes | Yes | Yes | — | — | — | — | — | — | — | — | Phlebotomy | — | 10 |
| 17 | p.Gly99Arg (p.G99R) | p.Leu101Pro (p.L101P) | c.295G>A | c.302T>C | Female | 26 | 26 | Yes | 2500 | 100 |  | Albanian | Yes | — | — | Yes | — | — | — | — | — | — | — | No | — | — | 5 |
| 18 | p.Leu101Pro (p.L101P) | p.Leu101Pro (p.L101P) | c.302T>C | c.302T>C | Female | 23 | 13 | No | 2647 | 90 |  | Southeast United States | No | No | Yes | Yes | — | Yes | Yes | — | — | — | — | No | Phlebotomy, sex hormone replacement, L-thyroxine | Achieved iron depletion, resolution of hyperpigmentation and regression of hepatomegaly. | 6 |
| 19 | p.Leu101Pro (p.L101P) | p.Leu101Pro (p.L101P) | c.302T>C | c.302T>C | Male | 21 | 15 | No | 4425 | 97 | *HFE*: H63D/H63H | Southeast United States | Yes | Yes, freckles | Yes | Yes | — | Yes | No | — | Yes | — | Yes | Yes | Phlebotomy, sex hormone replacement | Achieved resolution of hyperpigmentation and regression of hepatomegaly. Significant improvement in cardiomyopathy with phlebotomy and medical therapy for heart failure. | 6 |
| 20 | p.Leu101Pro (p.L101P) | p.Leu101Pro (p.L101P) | c.302T>C | c.302T>C | Female | 18 | 12 | No | 2003 | 92 |  | Southeast United States | No | Yes | — | Yes | — | Yes | No | — | — | — | — | No | Phlebotomy, sex hormone replacement | Achieved resolution of hyperpigmentation and regression of hepatomegaly. | 6 |
| 21 | p.Leu101Pro (p.L101P) | p.Leu101Pro (p.L101P) | c.302T>C | c.302T>C | Female | 8 | — | No | 1047 | 90 |  | Southeast United States | No | Yes, freckles | — | No | — | — | Yes | — | Yes | — | — | No | Phlebotomy, L-thyroxine | Achieved resolution of hyperpigmentation and regression of hepatomegaly. Repeatedly liver biopsy showing normal liver iron stain. | 6 |
| 22 | p.Leu101Pro (p.L101P) | p.Lys299Glu (p.K299E) | c.302T>C | c.895A>G | Female | 27 | — | Yes | — | 85 |  | France | Yes | Yes | Yes | Yes | Yes | — | — | — | Yes | No | — | Yes | Phlebotomy | — | 2 |
| 23 | p.Leu101Pro (p.L101P) | p.His180Arg (p.H180R) | c.302T>C | c.539A>G | Male | 31 | — | Yes | 2300 | 100 |  | France | — | — | Yes | — | — | — | — | — | Yes | — | — | Yes | Phlebotomy | — | 2 |
| 24 | p.Leu101Pro (p.L101P) | p.Ala384Val (p.A384V) | c.302T>C | c.1151C>T | Female | 60 | — | Yes | 4800 | 86 |  | France | — | Yes | — | — | Yes | — | — | — | Yes | — | — | — | Phlebotomy | — | 2 |
| 25 | p.Gln116Ter (p.Q116*) | p.Gly320Val (p.G320V) | c.346C>T | c.959G>T | Female | 16 | 16 | Yes | 646 | 75.4 |  | Ireland | Yes | — | — | Yes | Yes | — | Yes | — | Yes | Yes | Yes | Yes | Phlebotomy, deferoxamine, sex hormone replacement, L-thyroxine | Completely Reversed the cardiovascular symptoms and signs and heart function returned to normal. Hepatic iron stores were fully depleted but fibrosis remained unchanged. | 11 |
| 26 | p.Cys119Phe (p.C119F) | p.Cys119Phe (p.C119F) | c.356G>T | c.356G>T | Male | 25 | — | Yes | 1980 | 100 |  | Germany | Yes | No | — | No | — | — | — | — | — | No | — | — | — | — | 12 |
| 27 | p.Arg131PhefsTer111 (p.R131fs) | p.Arg131PhefsTer111 (p.R131fs) | c.391_403del | c.391_403del | Female | 20 | 20 | Yes | 4840 | 100 | *HFE*: H63D/H63H | Italy | Yes | No | No | Yes | Yes | — | — | — | — | — | — | No | — | — | 5 |
| 28 | p.Asp149ThrfsTer97 (p.D149fs) | p.Asp149ThrfsTer97 (p.D149fs) | c.445delG | c.445delG | Male | 21 | 21 | Yes | 2850 | 100 |  | Italy | No | No | No | Yes | Yes | — | — | — | Yes | — | Yes | Yes | Phlebotomy | — | 5 |
| 29 | p.Asp149ThrfsTer97 (p.D149fs) | p.Asp149ThrfsTer97 (p.D149fs) | c.445delG | c.445delG | Male | 22 | 22 | Yes | 2800 | 95 |  | Italy | No | — | — | Yes | — | — | — | — | — | Yes | — | Yes | — | — | 5 |
| 30 | p.Leu165Ter (p.L165*) | p.Leu165Ter (p.L165*) | c.494T>A | c.494T>A | Male | 16 | 16 | No | — | 93 | *HFE*: C282Y/C282C | Netherland | Yes | Yes, freckles | Yes | — | Yes | — | — | No | Yes | No | — | Yes | Phlebotomy, metformin | Achieved iron depletion. Developed abnormal heart function and diabetes. | 13, 14 |
| 31 | p.Leu165Ter (p.L165*) | p.Gly320Val (p.G320V) | c.494T>A | c.959G>T | Male | 24 | 21 | Yes | 4000 | 99 |  | Netherland | No | — | Yes | Yes | No | Yes | No | Yes | Yes | No | — | — | Phlebotomy, erthrocytapheresis, choriongonadotrophin | Achieved iron depletion and improved bone density of the lumbar spine. Arthralgia decreased but didn't completely disappear. | 13 |
| 32 | p.Leu165Ter (p.L165*) | p.Gly320Val (p.G320V) | c.494T>A | c.959G>T | Male | 20 | 17 | No | 1188 | 90 |  | Netherland | No | — | Yes | No | No | Yes | No | No | Yes | No | — | — | Phlebotomy | Achieved partial iron depletion, improved bone density of the lumbar spine, and resolution of arthralgia. | 13 |
| 33 | p.Leu165Ter (p.L165*) | p.Gly320Val (p.G320V) | c.494T>A | c.959G>T | Female | 29 | 29 | Yes | 2854 | 91 |  | Netherland | — | — | Yes | No | No | Yes | No | No | — | No | No | — | Phlebotomy | Achieved partial iron depletion. Doing well. | 13 |
| 34 | p.His166Arg (p.H166R) | p.His166Arg (p.H166R) | c.497A>G | c.497A>G | Male | 21 | 21 | Yes | 7722 | 95 |  | Arab | Yes | — | — | Yes | No | — | No | Yes | — | Yes | — | No | Phlebotomy, deferiprone, deferoxamine | Achieved iron depletion and improved liver function test. Waiting for a liver and heart transplant. | 15 |
| 35 | p.His166Arg (p.H166R) | p.His166Arg (p.H166R) | c.497A>G | c.497A>G | Male | 32 | 32 | No | 9685 | 94 |  | Arab | — | — | — | No | No | — | No | Yes | — | Yes | — | — | Phlebotomy | — | 15 |
| 36 | p.Ala168Asp (p.A168D) | p.Ala168Asp (p.A168D) | c.503C>A | c.503C>A | Male | 28 | 28 | Yes | — | — |  | Australia/England | No | — | — | Yes | — | — | — | — | — | — | — | No | — | — | 5 |
| 37 | p.Phe170Ser (p.F170S) | p.Phe170Ser (p.F170S) | c.509T>C | c.509T>C | Female | 20 | 20 | Yes | 1400 | 82 |  | Italy | No | Yes | No | Yes | No | — | — | — | Yes | — | Yes | Yes | Phlebotomy | — | 5 |
| 38 | p.Phe170Ser (p.F170S) | p.Phe170Ser (p.F170S) | c.509T>C | c.509T>C | Female | 14 | 14 | Yes | 3280 | 92 |  | Italy | No | Yes | No | Yes | Yes | — | — | — | Yes | Yes | — | Yes | — | — | 5 |
| 39 | p.Asp172Glu (p.D172E) | p.Cys321ValfsTer21 (p.C321fs) | c.516C>G | c.960dupG | Female | 20 | 20 | Yes | 2500 | — |  | Italy | Yes | — | — | Yes | — | — | — | — | — | — | — | No | — | — | 5 |
| 40 | p.Arg176Cys (p.R176C) | p.Arg176Cys (p.R176C) | c.526C>T | c.526C>T | Female | 17 | 17 | Yes | 2000 | 97 |  | France | — | — | Yes | Yes | — | — | — | — | — | — | Yes | Yes | — | — | 16 |
| 41 | p.Trp191Cys (p.W191C) | p.Trp191Cys (p.W191C) | c.573G>T | c.573G>T | Female | 21 | 21 | Yes | 3500 | 84 |  | Italy | No | Yes | No | Yes | No | — | — | — | Yes | Yes | — | Yes | — | — | 5 |
| 42 | p.Pro192Leu (p.P192L) | p.Pro192Leu (p.P192L) | c.575C>T | c.575C>T | Male | 23 | 23 | Yes | >6000 | — |  | Pakistan | — | — | Yes | Yes | — | — | — | — | Yes | — | Yes | Yes | — | — | 8 |
| 43 | p.Leu194Pro (p.L194P) | p.Leu194Pro (p.L194P) | c.581T>C | c.581T>C | Male | 32 | 32 | Yes | 3756 | 100 |  | Pakistan | — | — | — | Yes | No | — | — | Yes | Yes | Yes | — | Yes | — | — | 8 |
| 44 | p.Ser205Arg (p.S205R) | p.Gly250Val (p.G250V) | c.615C>G | c.749G>T | Female | 21 | 21 | Yes | 2300 | 75 |  | Italy | Yes | Yes | No | Yes | Yes | — | — | — | Yes | Yes | — | Yes | — | — | 5 |
| 45 | p.Ile222Asn (p.I222N) | p.Gly320Val (p.G320V) | c.665T>A | c.959G>T | Female | 23 | 17 | Yes | — | 85 |  | Southeast United States | Yes | Yes, hyperpigmentation and freckles | — | Yes | — | — | — | — | Yes | — | Yes | Yes | Phlebotomy, sex hormone replacement | Die of complications of cardiomyopathy. | 6 |
| 46 | p.Lys234del (p.K234del) | p.Lys234del (p.K234del) | c.700_702delAAG | c.700_702delAAG | Female | 20 | — | Yes | 1955 | — |  | Europe | Yes | Yes | Yes | Yes | — | Yes | — | Yes | Yes | — | Yes | Yes | Phlebotomy, sex hormone replacement | Achieved iron depletion. Hypogonadism remained. A slight anemia and new arthralgia developed. | 17 |
| 47 | p.Ile281Thr (p.I281T) | p.Ile281Thr (p.I281T) | c.842T>C | c.842T>C | Female | 49 | 39 | Yes | 4127 | 90 |  | Greece | No | Yes | Yes | Yes | No | — | — | — | Yes | Yes | — | Yes | Phlebotomy | — | 10 |
| 48 | p.Arg288Trp (p.R288W) | p.Arg288Trp (p.R288W) | c.862C>T | c.862C>T | Female | 26 | — | Yes | 12500 | 100 | *HFE*: V285A/V285V | France | Yes | — | — | Yes | — | — | — | — | Yes | Yes | — | Yes | Gonadotrophin, die after diagnosis | Died of sepsis resulting from infection after diagnosis. | 5, 18 |
| 49 | p.Arg288Trp (p.R288W) | p.Arg288Trp (p.R288W) | c.862C>T | c.862C>T | Female | 24 | — | No | 1940 | 100 | *HFE*: V285A/V285V | France | — | — | — | Yes | — | — | — | — | — | — | — | — | Phlebotomy | Achieved iron depletion and normal hematological parameters. | 18 |
| 50 | p.Arg288Trp (p.R288W) | p.Arg288Trp (p.R288W) | c.862C>T | c.862C>T | Male | 32 | — | Yes | 4959 | 90 |  | France | — | — | — | Yes | — | — | — | — | Yes | — | — | — | Phlebotomy | — | 2 |
| 51 | p.Cys317Ser (p.C317S) | p.Cys317Ser (p.C317S) | c.950G>C | c.950G>C | Female | 68 | 35 | Yes | 3987 | 99 |  | Italy | Yes | Yes | Yes | — | Yes | — | — | No | Yes | — | Yes | Yes | Erthrocytapheresis, deferoxamine, insulin | Achieved partial iron depletion and normal heart function. | 19 |
| 52 | p.Gly320Val (p.G320V) | p.Gly320Val (p.G320V) | c.959G>T | c.959G>T | Male | 28 | — | Yes | 2900 | 96 |  | Kosovo | Yes | — | Yes | Yes | — | — | No | Yes | Yes | Yes | — | Yes | Phlebotomy | Achieved iron depletion and normal heart function. | 20 |
| 53 | p.Gly320Val (p.G320V) | p.Gly320Val (p.G320V) | c.959G>T | c.959G>T | Female | 28 | 27 | Yes | 4302 | 96 |  | Germany | — | Yes | — | No | Yes | — | No | Yes | Yes | Yes | Yes | Yes | Phlebotomy | — | 21 |
| 54 | p.Gly320Val (p.G320V) | p.Gly320Val (p.G320V) | c.959G>T | c.959G>T | Female | 12 | — | Yes | 3671 | 81 |  | Denmark | — | — | — | — | — | — | — | — | Yes | — | — | Yes | Phlebotomy | Achieved favorable effect. | 22 |
| 55 | p.Gly320Val (p.G320V) | p.Gly320Val (p.G320V) | c.959G>T | c.959G>T | Female | 10 | — | No | 1356 | 80 |  | Denmark | — | — | — | — | — | — | — | — | Yes | — | — | Yes | Phlebotomy | Achieved favorable effect. | 22 |
| 56 | p.Gly320Val (p.G320V) | p.Gly320Val (p.G320V) | c.959G>T | c.959G>T | Female | 39 | 20 | Yes | 116070 | 100 |  | United States | Yes | — | — | Yes | — | — | Yes | Yes | Yes | Yes | Yes | Yes | Phlebotomy, sex hormone and thyroid hormone replacement | Symptoms resolved except for persistent fatigue. Died of heart failure after diagnosis. | 23 |
| 57 | p.Gly320Val (p.G320V) | p.Gly320Val (p.G320V) | c.959G>T | c.959G>T | Male | 30 | — | Yes | 9025 | 92 |  | Belgium | Yes | Yes | — | Yes | Yes | Yes | Yes | Yes | — | — | Yes | Yes | Phlebotomy, deferoxamine, insulin | Achieved partial iron depletion. Heart function remained unchanged. | 24 |
| 58 | p.Gly320Val (p.G320V) | p.Gly320Val (p.G320V) | c.959G>T | c.959G>T | Male | 28 | — | Yes | 5484 | 95 | *HFE*: C282Y/C282C | Canada | Yes | Yes | — | Yes | — | — | — | — | — | — | — | — | Phlebotomy | — | 25 |
| 59 | p.Gly320Val (p.G320V) | p.Gly320Val (p.G320V) | c.959G>T | c.959G>T | Male | 37 | — | Yes | 11000 | 100 | *HFE*: C282Y/C282C | Canada | Yes | — | — | — | — | — | — | — | — | — | — | — | Phlebotomy | — | 25 |
| 60 | p.Gly320Val (p.G320V) | p.Gly320Val (p.G320V) | c.959G>T | c.959G>T | Female | 18 | — | Yes | 2329 | 100 |  | United States | — | — | — | — | — | — | — | — | — | — | — | — | Phlebotomy | — | 26 |
| 61 | p.Gly320Val (p.G320V) | p.Gly320Val (p.G320V) | c.959G>T | c.959G>T | Male | 12 | — | No | >1000 | 100 |  | United States | — | — | — | — | — | — | — | — | — | — | — | — | — | — | 26 |
| 62 | p.Gly320Val (p.G320V) | p.Gly320Val (p.G320V) | c.959G>T | c.959G>T | Male | 16 | — | No | >1000 | 100 |  | United States | — | — | — | — | — | — | — | — | — | — | — | — | — | — | 26 |
| 63 | p.Gly320Val (p.G320V) | p.Gly320Val (p.G320V) | c.959G>T | c.959G>T | Female | 28 | 24 | Yes | 3541.8 | 85 |  | Hungary | No | No | Yes | Yes | — | — | No | Yes | No | — | — | — | Phlebotomy, deferoxamine | Achieved partial iron depletion and normal liver function test. | 27 |
| 64 | p.Gly320Val (p.G320V) | p.Gly320Val (p.G320V) | c.959G>T | c.959G>T | Female | 12 | 12 | Yes | 4050 | 99 | *HFE*: H63D/H63H | Australia | Yes | — | — | Yes | — | — | — | Yes | Yes | Yes | — | Yes | — | — | 7 |
| 65 | p.Gly320Val (p.G320V) | p.Gly320Val (p.G320V) | c.959G>T | c.959G>T | Female | 32 | 32 | Yes | 2000 | 88 | *HFE*: C282Y/C282C | Australia | — | — | Yes | Yes | — | Yes | — | Yes | Yes | — | Yes | Yes | — | — | 7 |
| 66 | p.Gly320Val (p.G320V) | p.Gly320Val (p.G320V) | c.959G>T | c.959G>T | Male | 24 | — | Yes | 13700 | 100 | *HFE*: C282Y/C282C | Croatia | Yes | Yes | — | Yes | — | — | — | — | — | Yes | Yes | — | — | — | 12 |
| 67 | p.Gly320Val (p.G320V) | p.Gly320Val (p.G320V) | c.959G>T | c.959G>T | Male | 24 | — | Yes | — | 94 |  | Germany | No | No | — | Yes | — | — | — | — | — | Yes | — | — | — | — | 12 |
| 68 | p.Gly320Val (p.G320V) | p.Gly320Val (p.G320V) | c.959G>T | c.959G>T | Male | 24 | — | Yes | 6400 | 100 |  | Germany | No | Yes | — | No | — | — | — | — | — | No | — | — | — | — | 12 |
| 69 | p.Gly320Val (p.G320V) | p.Gly320Val (p.G320V) | c.959G>T | c.959G>T | Female | 28 | — | Yes | 4300 | 96 |  | Germany | No | Yes | — | No | — | — | — | — | — | Yes | — | — | — | — | 12 |
| 70 | p.Gly320Val (p.G320V) | p.Gly320Val (p.G320V) | c.959G>T | c.959G>T | Male | 22 | 22 | Yes | 3500 | 99 | *HFE*: H63D/H63H | [Brazil](https://fanyi.so.com/?src=onebox#Brazil) | No | — | Yes | Yes | Yes | — | — | Yes | Yes | — | — | — | Phlebotomy, deferasirox | Achieved partial iron depletion, the resolution of all initial clinical symptoms, normalization of endocrine and liver function, and reduced liver iron. | 28, 29 |
| 71 | p.Gly320Val (p.G320V) | p.Gly320Val (p.G320V) | c.959G>T | c.959G>T | Female | 26 | 18 | Yes | 6018 | — |  | Romania | Yes | — | — | Yes | — | — | — | Yes | — | — | — | — | — | — | 30 |
| 72 | p.Gly320Val (p.G320V) | p.Gly320Val (p.G320V) | c.959G>T | c.959G>T | Male | 25 | 21 | Yes | 2283 | 100 |  | Greece | No | Yes | Yes | Yes | No | — | — | Yes | Yes | Yes | — | Yes | Phlebotomy | — | 10 |
| 73 | p.Gly320Val (p.G320V) | p.Gly320Val (p.G320V) | c.959G>T | c.959G>T | Female | 39 | 32 | Yes | 3553 | 100 |  | Greece | Yes | Yes | Yes | Yes | Yes | — | — | — | Yes | Yes | — | Yes | Phlebotomy | — | 10 |
| 74 | p.Gly320Val (p.G320V) | p.Gly320Val (p.G320V) | c.959G>T | c.959G>T | Male | 32 | 25 | Yes | 2500 | 100 |  | Greece | Yes | Yes | Yes | Yes | Yes | — | — | — | Yes | Yes | — | Yes | Inadequately treated with deferoxamine, sex hormone replacement | Died of cardiac failure. | 10 |
| 75 | p.Gly320Val (p.G320V) | p.Gly320Val (p.G320V) | c.959G>T | c.959G>T | Female | 21 | 20 | Yes | — | 100 |  | Greece | No | Yes | No | Yes | No | — | — | — | — | — | — | No | Phlebotomy | Remain asymptomatic. | 10 |
| 76 | p.Gly320Val (p.G320V) | p.Gly320Val (p.G320V) | c.959G>T | c.959G>T | Male | 25 | 21 | Yes | 5250 | 100 |  | Greece | No | No | No | Yes | No | Yes | — | Yes | Yes | Yes | Yes | Yes | — | — | 10 |
| 77 | p.Gly320Val (p.G320V) | p.Gly320Val (p.G320V) | c.959G>T | c.959G>T | Male | 31 | 31 | Yes | 2541 | 90 | *HFE*: H63D/H63H | Serbia | Yes | — | — | Yes | Yes | — | — | Yes | Yes | Yes | — | Yes | Phlebotomy, deferoxamine, sex hormone replacement, insulin | Achieved iron depletion and heart failure nearly complete recovery. | 31 |
| 78 | p.Gly320Val (p.G320V) | p.Gly320Val (p.G320V) | c.959G>T | c.959G>T | Male | 29 | 29 | Yes | 5665 | 100 |  | Italy | No | Yes | No | Yes | No | — | — | — | Yes | — | Yes | Yes | — | — | 5 |
| 79 | p.Gly320Val (p.G320V) | p.Gly320Val (p.G320V) | c.959G>T | c.959G>T | Female | 33 | 27 | Yes | >2500 | 100 | *HFE*: C282Y/C282C | Canada, Saguenay-Lac-Saint-Jean | Yes | Yes | Yes | Yes | No | — | — | — | — | — | — | No | — | — | 5 |
| 80 | p.Gly320Val (p.G320V) | p.Gly320Val (p.G320V) | c.959G>T | c.959G>T | Female | 39 | 39 | Yes | 6200 | 98 |  | Netherland | No | — | Yes | — | No | — | No | Yes | Yes | Yes | — | Yes | Phlebotomy | Achieved partial iron depletion and normalized liver function test. Arthralgia decreased but not completely disappeared. | 13 |
| 81 | p.Gly320Val (p.G320V) | p.Gly320Val (p.G320V) | c.959G>T | c.959G>T | Male | 31 | 31 | Yes | 2680 | . |  | Netherland | No | — | Yes | Considered but not necessary | No | Considered but not necessary | No | Yes | Yes | Yes | — | Yes | Phlebotomy | Achieved iron depletion. Doing well. | 13 |
| 82 | p.Gly320Val (p.G320V) | p.Gly320Val (p.G320V) | c.959G>T | c.959G>T | Male | 28 | — | Yes | 3300 | 58 |  | France | — | — | — | Yes | — | — | — | — | Yes | — | — | — | Phlebotomy, sex hormone replacement | — | 2 |
| 83 | p.Gly320Val (p.G320V) | p.Gly320Val (p.G320V) | c.959G>T | c.959G>T | Male | 31 | — | Yes | 3700 | 91 |  | France | — | — | — | — | — | — | — | — | Yes | Yes | — | Yes | Phlebotomy | — | 2 |
| 84 | p.Gly320Val (p.G320V) | p.Gly320Val (p.G320V) | c.959G>T | c.959G>T | Female | 16 | — | Yes | 2270 | 89 | *HFE*: C282Y/C282C | France | — | Yes | — | Yes | — | — | — | — | Yes | — | — | — | Phlebotomy | — | 2 |
| 85 | p.Gly320Val (p.G320V) | p.Cys321Trp (p.C321W) | c.959G>T | c.963C>G | Female | 30 | 23 | Yes | — | 76 |  | United States | No | Yes | Yes | Yes | Yes | — | Yes | — | Yes | — | Yes | Yes | Phlebotomy, thyroxine, oral anti-diabetic drugs | Achieved iron depletion. | 32 |
| 86 | p.Gly320Val (p.G320V) | p.Ser328AspfsTer10 (p.S328fs) | c.959G>T | c.982_985delTCTC | Male | 25 | — | Yes | — | 100 |  | Slovakia | No | No | — | Yes | — | — | — | — | — | Yes | — | — | — | — | 12 |
| 87 | p.Gly320Val (p.G320V) | p.Ser328AspfsTer10 (p.S328fs) | c.959G>T | c.982_985delTCTC | Female | 16 | — | No | — | 74 |  | Slovakia | No | No | — | No | — | — | — | — | — | Yes | — | — | — | — | 12 |
| 88 | p.Gly336Ter (p.G336*) | p.Gly336Ter (p.G336*) | c.1006G>T | c.1006G>T | Male | 45 | — | Yes | 3136 | 94 | *HFE*: H63D/H63H | India | — | Yes | — | — | Yes | — | — | No | Yes | — | — | — | Phlebotomy | — | 33 |
| 89 | p.Gly336Ter (p.G336*) | p.Gly336Ter (p.G336*) | c.1006G>T | c.1006G>T | Female | 49 | — | Yes | 1700 | 100 |  | India | — | — | — | — | — | — | — | No | Yes | — | — | — | Phlebotomy | — | 33 |
| 90 | p.Gly336Ter (p.G336*) | p.Gly336Ter (p.G336*) | c.1006G>T | c.1006G>T | Female | 38 | — | Yes | 9400 | 69 |  | India | — | — | — | — | Yes | — | Yes | Yes | Yes | — | Yes | — | Phlebotomy | — | 33 |
| 91 | p.Gly336Ter (p.G336*) | p.Gly336Ter (p.G336*) | c.1006G>T | c.1006G>T | Male | 47 | — | Yes | 12433 | 91 |  | India | Yes | Yes | Yes | Yes | Yes | — | — | Yes | Yes | — | — | — | Phlebotomy | — | 33 |
| 92 | p.Ala343ProfsTer24 (p.A343fs) | p.Ala343ProfsTer24 (p.A343fs) | c.1026delT | c.1026delT | Male | 17 | 17 | Yes | 5999 | 94 |  | Sri Lanka | — | Yes | — | Yes | Yes | — | — | Yes | Yes | Yes | — | Yes | — | — | 8 |
| 93 | p.Cys361ValfsTer6 (p.C361fs) | p.Cys361ValfsTer6 (p.C361fs) | c.1080delC | c.1080delC | Male | 33 | 26 | Yes | 5900 | 98 |  | Greece | No | Yes | No | Yes | No | — | — | — | Yes | Yes | Yes | Yes | Phlebotomy | — | 10 |
| 94 | p.Leu366Ter (p.L366*) | p.Leu366Ter (p.L366*) | c.1097T>A | c.1097T>A | Male | 19 | — | Yes | 7000 | 100 |  | Canada | Yes | — | — | — | — | — | — | — | — | — | — | — | Phlebotomy | — | 25 |
| 95 | p.Arg385Ter (p.R385*) | p.Arg385Ter (p.R385*) | c.1153C>T | c.1153C>T | Female | 15 | 15 | Yes | 615 | 88 |  | Italy | No | Yes | No | No | Yes | — | — | — | Yes | Yes | — | Yes | — | — | 5 |
| 96 | p.Arg385Ter (p.R385*) | p.Arg385Ter (p.R385*) | c.1153C>T | c.1153C>T | Male | 20 | 20 | Yes | 2000 | — | *HFE*: H63D/H63H | Italy | No | Yes | No | Yes | No | — | — | — | — | — | — | No | Phlebotomy | — | 5 |
| 97 | 非编码区 | 非编码区 | 5'UTR-1624(G>A) | 5'UTR-36(G>A) | Male | 43 | — | Yes | 2800 | 79.4 |  | India | — | Yes | — | — | Yes | — | Yes | Yes | Yes | — | — | — | Phlebotomy | — | 33 |
| 98 | 非编码区 | 非编码区 | 5'UTR-1624(G>A) | 5'UTR-36(G>A) | Female | 32 | — | Yes | 4250 | 67.7 |  | India | — | — | — | Yes | Yes | — | Yes | Yes | Yes | — | — | — | — | — | 33 |
| **East Asians** | | | | | | | | | | | | | | | | | | | | | | | | | | | |
| 99 | p.[Gln6His;Cys321Ter] (p.[Q6H;C321*]) | p.[Gln6His;Cys321Ter] (p.[Q6H;C321*]) | c.[18G>C;962_963delinsAA] | c.[18G>C;962_963delinsAA] | Male | 29 | 19 | Yes | 1650 | — |  | China | Yes | Yes | — | Yes | Yes | — | — | — | — | — | — | — | — | — | 34 |
| 100 | p.[Gln6His;Cys321Ter] (p.[Q6H;C321*]) | p.Val274Met (p.V274M) | c.[18G>C;962_963delinsAA] | c.820G>A | Male | 57 | — | Yes | 4001 | 93 |  | China | — | Yes | — | — | — | — | — | Yes | Yes | Yes | — | Yes | Phlebotomy | — | 35 |
| 101 | p.[Gln6His;Cys321Ter] (p.[Q6H;C321*]) | p.Ile281Thr (p.I281T) | c.[18G>C;962_963delinsAA] | c.842T>C | Female | 19 | 14 | Yes | 7575 | 94 |  | China | Yes | Yes | — | Yes | Yes | — | No | Yes | Yes | Yes | Yes | Yes | Phlebotomy, sex hormone replacement, insulin | Achieved iron depletion, normalization of liver function test and heart function, and recovery of heart disease and diabetes. | 36 |
| 102 | p.[Gln6His;Cys321Ter] (p.[Q6H;C321*]) | p.Ile281Thr (p.I281T) | c.[18G>C;962_963delinsAA] | c.842T>C | Male | 26 | — | Yes | 7004 | 92.1 |  | China | Yes | — | — | — | Yes | — | — | — | Yes | — | — | Yes | — | — | 35 |
| 103 | p.[Gln6His;Cys321Ter] (p.[Q6H;C321*]) | p.Ile281Thr (p.I281T) | c.[18G>C;962_963delinsAA] | c.842T>C | Male | 27 | — | No | 6269 | 95.4 | *HFE*: H63D/H63H | China | — | Yes | — | Yes | — | — | — | Yes | Yes | — | — | Yes | — | — | 35 |
| 104 | p.[Gln6His;Cys321Ter] (p.[Q6H;C321*]) | p.His104Arg (p.H104R) | c.[18G>C;962_963delinsAA] | c.311A>G | Male | 18 | — | Yes | 6678 | 100 | *TFR2*: A75V/A75A | China | — | — | — | — | — | — | — | Yes | Yes | — | — | Yes | — | — | 35 |
| 105 | p.Tyr46Ter (p.Y46*) | p.Tyr46Ter (p.Y46*) | c.138C>A | c.138C>A | Male | 13 | — | Yes | 2337 | 99.1 |  | China | — | — | — | — | — | — | — | — | Yes | — | Yes | — | — | — | 37 |
| 106 | p.Phe103Leu (p.F103L) | p.Phe103Leu (p.F103L) | c.309C>G | c.309C>G | Female | 36 | — | Yes | 2000 | 96 |  | China | — | — | — | Yes | — | — | — | Yes | Yes | — | — | Yes | — | — | 35 |
| 107 | p.His174ProfsTer23 (p.H174fs) | p.His174ProfsTer23 (p.H174fs) | c.515_516insC | c.515_516insC | Male | 13 | 13 | Yes | 16000 | 90 |  | Japan | Yes | — | — | — | No | — | — | Yes | Yes | Yes | — | Yes | Phlebotomy, deferasirox | Achieved partial iron depletion, partial normalization of heart and liver function tests, and improved biochemical parameters gradually. | 38, 39 |
| 108 | p.His174ProfsTer23 (p.H174fs) | p.His174ProfsTer23 (p.H174fs) | c.515_516insC | c.515_516insC | Male | 17 | 12 | No | 2500 | 92 |  | Japan | No | — | — | — | Yes | — | — | Yes | — | — | — | No | Phlebotomy, insulin | Achieved partial iron depletion and normalized biochemical parameters. | 38 |
| 109 | p.Asp249His (p.D249H) | p.Asp249His (p.D249H) | c.745G>C | c.745G>C | Male | 48 | 48 | Yes | 6115 | 94.8 |  | Japan | Yes | Yes | No | No | Yes | — | — | Yes | Yes | — | Yes | Yes | Phlebotomy, insulin | Achieved improved liver damage and diabetes. Social active. | 38, 40 |
| 110 | p.Asp249His (p.D249H) | p.Gln312Ter (p.Q312*) | c.745G>C | c.934C>T | Male | 26 | — | Yes | 4354 | 75.9 | *TFR2*: A75V/A75A | Japan | — | — | — | — | Yes | — | — | Yes | — | — | — | — | — | — | 41 |
| 111 | p.Ile287Ser (p.I287S) | p.Ile287Ser (p.I287S) | c.860T>G | c.860T>G | Male | 37 | — | Yes | 4974 | 102.3 | *TFR2*: I238M/I238I | China | — | — | — | — | Yes | — | — | — | Yes | — | — | — | — | — | 37 |
| 112 | p.Gln312Ter (p.Q312*) | p.Gln312Ter (p.Q312*) | c.934C>T | c.934C>T | Female | 55 | — | No | 510 | 90 |  | Japan | — | — | — | — | — | — | — | — | — | — | — | — | — | — | 42 |
| 113 | p.Gln312Ter (p.Q312*) | p.Gln312Ter (p.Q312*) | c.934C>T | c.934C>T | Male | 22 | — | No | — | — |  | Japan | Yes | — | — | — | — | — | — | — | — | — | — | — | — | — | 42 |
| 114 | p.Gln312Ter (p.Q312*) | p.Gln312Ter (p.Q312*) | c.934C>T | c.934C>T | Male | 24 | 24 | Yes | 5520 | 92 |  | Japan | Yes | — | — | Yes | — | — | — | — | Yes | — | — | — | Phlebotomy | Achieved partial iron depletion. Complete atrioventricular block and required implantation of a permanent pacemaker. | 42 |
| 115 | p.Gln312Ter (p.Q312*) | p.Gln312Ter (p.Q312*) | c.934C>T | c.934C>T | Male | 51 | 51 | Yes | 2280 | 95.5 |  | Japan | Yes | Yes | — | — | Yes | — | — | — | Yes | — | Yes | Yes | Phlebotomy, insulin | Die of sepsis. | 40 |
| 116 | p.Gln312Ter (p.Q312*) | p.Gln312Ter (p.Q312*) | c.934C>T | c.934C>T | Female | 51 | 49 | No | 4278 | 95.8 |  | Japan | — | Yes | — | — | Yes | — | — | — | Yes | — | Yes | Yes | Phlebotomy | — | 40 |
| **African** | | | | | | | | | | | | | | | | | | | | | | | | | | | |
| 117 | p.Arg385Ter (p.R385*) | p.Arg385Ter (p.R385*) | c.1153C>T | c.1153C>T | Male | 8 | — | Yes | 282 | 83 |  | North African | — | — | — | — | — | — | — | — | Yes | — | — | — | Deferasirox or deferoxamine | — | 2 |

HGVS, Human Genome Variation Society; *HJV*-HH, *HJV* related hereditary hemochromatosis.

**References:**

1. Lee P, Promrat K, Mallette C, Flynn M, Beutler E. A juvenile hemochromatosis patient homozygous for a novel deletion of cDNA nucleotide 81 of hemojuvelin. Acta Haematol 2006;115(1-2):123-7.

2. Hamdi-Roze H, Ben AZ, Ropert M, et al. Variable expressivity of HJV related hemochromatosis: "Juvenile" hemochromatosis? Blood Cells Mol Dis 2018.

3. Neroldova M, Frankova S, Stranecky V, et al. Hereditary haemochromatosis caused by homozygous HJV mutation evolved through paternal disomy. Clin Genet 2015;87(1):96-8.

4. Janosi A, Andrikovics H, Vas K, et al. Homozygosity for a novel nonsense mutation (G66X) of the HJV gene causes severe juvenile hemochromatosis with fatal cardiomyopathy. Blood 2005;105(1):432.

5. Lanzara C, Roetto A, Daraio F, et al. Spectrum of hemojuvelin gene mutations in 1q-linked juvenile hemochromatosis. Blood 2004;103(11):4317-21.

6. Lee PL, Beutler E, Rao SV, Barton JC. Genetic abnormalities and juvenile hemochromatosis: mutations of the HJV gene encoding hemojuvelin. Blood 2004;103(12):4669-71.

7. Wallace DF, Dixon JL, Ramm GA, Anderson GJ, Powell LW, Subramaniam N. Hemojuvelin (HJV)-associated hemochromatosis: analysis of HJV and HFE mutations and iron overload in three families. Haematologica 2005;90(2):254-5.

8. Lok CY, Merryweather-Clarke AT, Viprakasit V, et al. Iron overload in the Asian community. Blood 2009;114(1):20-5.

9. Malekzadeh MM, Radmard AR, Nouroozi A, et al. Juvenile Hemochromatosis, Genetic Study and Long-term Follow up after Therapy. Middle East J Dig Dis 2014;6(2):87-92.

10. Papanikolaou G, Samuels ME, Ludwig EH, et al. Mutations in HFE2 cause iron overload in chromosome 1q-linked juvenile hemochromatosis. Nat Genet 2004;36(1):77-82.

11. Daraio F, Ryan E, Gleeson F, Roetto A, Crowe J, Camaschella C. Juvenile hemochromatosis due to G320V/Q116X compound heterozygosity of hemojuvelin in an Irish patient. Blood Cells Mol Dis 2005;35(2):174-6.

12. Gehrke SG, Pietrangelo A, Kascak M, et al. HJV gene mutations in European patients with juvenile hemochromatosis. Clin Genet 2005;67(5):425-8.

13. Smit SL, Peters T, Gisbertz I, et al. Variable workup calls for guideline development for type 2A hereditary haemochromatosis. Neth J Med 2018;76(8):365-73.

14. van Dijk BA, Kemna EH, Tjalsma H, et al. Effect of the new HJV-L165X mutation on penetrance of HFE. Blood 2007;109(12):5525-6.

15. Ramzan K, Imtiaz F, Al-Ashgar HI, AlSayed M, Sulaiman RA. Juvenile hemochromatosis and hepatocellular carcinoma in a patient with a novel mutation in the HJV gene. Eur J Med Genet 2017;60(6):308-11.

16. Ka C, Le Gac G, Letocart E, Gourlaouen I, Martin B, Ferec C. Phenotypic and functional data confirm causality of the recently identified hemojuvelin p.r176c missense mutation. Haematologica 2007;92(9):1262-3.

17. Lee PL, Beutler E. Regulation of hepcidin and iron-overload disease. Annu Rev Pathol 2009;4:489-515.

18. Filali M, Le Jeunne C, Durand E, et al. Juvenile hemochromatosis HJV-related revealed by cardiogenic shock. Blood Cells Mol Dis 2004;33(2):120-4.

19. Ravasi G, Pelucchi S, Mariani R, Silvestri L, Camaschella C, Piperno A. A severe hemojuvelin mutation leading to late onset of HFE2-hemochromatosis. Dig Liver Dis 2018;50(8):859-62.

20. Burri E, Decker M, Eriksson U, Buser P, Hunziker L. [28-year old patient with successfully treated dilatative cardiomyopathy]. Internist (Berl) 2008;49(3):349-52.

21. Eisold M, Gehrke S, Stremmel W, Gugler R. [A young diabetic with small-nodule liver cirrhosis, high transferrin saturation and negative HFE test]. Dtsch Med Wochenschr 2005;130(24):1494-6.

22. Berg LB, Milman NT, Friis-Hansen L, Jensen PD, Frund T. [Juvenile haemochromatosis caused by a homozygous Gly320Val mutation in the haemojuvelin gene]. Ugeskr Laeger 2013;175(16):1113-4.

23. Cherfane C, Lee P, Guerin L, Brown K. A late presentation of a fatal disease: juvenile hemochromatosis. Case Rep Med 2013;2013:875093.

24. Pauwels R, Vandecasteele E, Devos D, Pauwels W, De Pauw M. An unexpected cause of liver cirrhosis and cardiomyopathy in a young man. Acta Clin Belg 2018;73(5):393-7.

25. Lanktree MB, Sadikovic B, Waye JS, et al. Clinical evaluation of a hemochromatosis next-generation sequencing gene panel. Eur J Haematol 2017;98(3):228-34.

26. Farrell CP, Parker CJ, Phillips JD. Exome sequencing for molecular characterization of non-HFE hereditary hemochromatosis. Blood Cells Mol Dis 2015;55(2):101-3.

27. Varkonyi J, Lueff S, Szucs N, et al. Hemochromatosis and hemojuvelin G320V homozygosity in a Hungarian woman. Acta Haematol 2010;123(3):191-3.

28. Santos PC, Cancado RD, Pereira AC, Chiattone CS, Krieger JE, Guerra-Shinohara EM. HJV hemochromatosis, iron overload, and hypogonadism in a Brazilian man: treatment with phlebotomy and deferasirox. Acta Haematol 2010;124(4):204-5.

29. Santos PC, Cancado RD, Pereira AC, et al. Hereditary hemochromatosis: mutations in genes involved in iron homeostasis in Brazilian patients. Blood Cells Mol Dis 2011;46(4):302-7.

30. Militaru MS, Popp RA, Trifa AP. Homozygous G320V mutation in the HJV gene causing juvenile hereditary haemochromatosis type A. A case report. J Gastrointestin Liver Dis 2010;19(2):191-3.

31. Cooray SD, Heerasing NM, Selkrig LA, et al. Reversal of end-stage heart failure in juvenile hemochromatosis with iron chelation therapy: a case report. J Med Case Rep 2018;12(1):18.

32. Lee PL, Barton JC, Brandhagen D, Beutler E. Hemojuvelin (HJV) mutations in persons of European, African-American and Asian ancestry with adult onset haemochromatosis. Br J Haematol 2004;127(2):224-9.

33. Dhillon BK, Chopra G, Jamwal M, et al. Adult onset hereditary hemochromatosis is associated with a novel recurrent Hemojuvelin (HJV) gene mutation in north Indians. Blood Cells Mol Dis 2018;73:14-21.

34. Yuanfeng L, Hongxing Z, Haitao Z, et al. [Mutation analysis of the pathogenic gene in a Chinese family with hereditary hemochromatosis]. Yi Chuan 2014;36(11):1152-8.

35. Lv T, Zhang W, Xu A, et al. Non-HFE mutations in haemochromatosis in China: combination of heterozygous mutations involving HJV signal peptide variants. J Med Genet 2018;55(10):650-60.

36. Huang FW, Rubio-Aliaga I, Kushner JP, Andrews NC, Fleming MD. Identification of a novel mutation (C321X) in HJV. Blood 2004;104(7):2176-7.

37. Wang Y, Du Y, Liu G, et al. Identification of novel mutations in HFE, HFE2, TfR2, and SLC40A1 genes in Chinese patients affected by hereditary hemochromatosis. Int J Hematol 2017;105(4):521-5.

38. Hattori A, Miyajima H, Tomosugi N, Tatsumi Y, Hayashi H, Wakusawa S. Clinicopathological study of Japanese patients with genetic iron overload syndromes. Pathol Int 2012;62(9):612-8.

39. Maeda T, Nakamaki T, Saito B, et al. Hemojuvelin hemochromatosis receiving iron chelation therapy with deferasirox: improvement of liver disease activity, cardiac and hematological function. Eur J Haematol 2011;87(5):467-9.

40. Koyama C, Hayashi H, Wakusawa S, et al. Three patients with middle-age-onset hemochromatosis caused by novel mutations in the hemojuvelin gene. J Hepatol 2005;43(4):740-2.

41. Ikuta K, Hatayama M, Addo L, et al. Iron overload patients with unknown etiology from national survey in Japan. Int J Hematol 2017;105(3):353-60.

42. Nagayoshi Y, Nakayama M, Suzuki S, et al. A Q312X mutation in the hemojuvelin gene is associated with cardiomyopathy due to juvenile haemochromatosis. Eur J Heart Fail 2008;10(10):1001-6.
