# Supplementary material for: Genotypic and phenotypic spectra of hemojuvelin mutations in primary hemochromatosis patients: a systematic review
Source: Orphanet J Rare Dis. 2019 Jul 8;14:171. doi: 10.1186/s13023-019-1097-2 (PMC6615163; doi:10.1186/s13023-019-1097-2)
Supplement: Supplementary file 4 — Clinical findings for cases with monoallelic mutation. (DOCX 56 kb) [file 13023_2019_1097_MOESM4_ESM.docx]

**Additional file 4.** Clinical findings for cases with monoallelic mutation.

| **ID** | **Amino acid change (HGVS nomenclature)** | **Nucleotide change (HGVS nomenclature)** | **Sex** | **Age at diagnosis** | **Age at presentation** | **Proband** | **SF (ng/ml)** | **TS (%)** | **Other gene variants** | **Family origin** | **Heart disease** | **Skin hyperpigmentation** | **Arthropathy** | **Endocrine abnormalities** | | | | **Liver abnormality** | | | | **Liver biopsy** | **Therapy** | **Outcomes** | **Reference** |
| --- | --- | --- | --- | --- | --- | --- | --- | --- | --- | --- | --- | --- | --- | --- | --- | --- | --- | --- | --- | --- | --- | --- | --- | --- | --- |
|  |  |  |  |  |  |  |  |  |  |  |  |  |  | **Hypogonadism** | **Glucose intolerance** | **Osteoporosis** | **Thyroid abnormality** | **Abnormal liver function test** | **Liver iron deposition** | **Liver fibrosis** | **Liver cirrhosis** |  |  |  |  |
| **Caucasians** | | | | | | | | | | | | | | | | | | | | | | | | | |
| 1 | p.Phe103SerfsTer11 (p.F103fs) | c.306delC | Female | 12 | 12 | Yes | 467 | 87 |  | Italy | No | No | No | — | No | — | — | — | Yes | — | — | — | Phlebotomy | Iron depletion was not achieved. Did not reach the menarche at age 13.5. | 1 |
| 2 | p.Asp149ThrfsTer97 (p.D149fs) | c.445delG | Female | 62 | — | Yes | 230 | 45 | *HFE*: C282Y/C282C | Italy | — | — | — | — | — | — | — | — | — | — | — | — | — | — | 2 |
| 3 | p.Asn196Lys (p.N196K) | c.588T>G | Male | 70 | 70 | No | 400 | — | *HFE*: C282Y/C282C | Italy | — | — | — | — | — | — | — | — | — | — | — | — | — | — | 2 |
| **East Asians** | | | | | | | | | | | | | | | | | | | | | | | | | |
| 4 | p.Glu3Asp (p.E3D) | c.9G>C | Male | 67 | — | Yes | 1102 | 92.3 | *HFE*: H63D/H63H *TFR2*: I238M/I238I *SUGP2*: R639Q/R639R *DENND3*: L708V/L708V | China | — | — | — | — | — | — | — | No | Yes | Yes | — | Yes | — | — | 3 |
| 5 | p.Glu3Asp (p.E3D) | c.9G>C | Female | 77 | — | No | 365 | 31.3 | *HFE*: H63D/H63H *TFR2*: I238M/I238I *SUGP2*: R639Q/R639R | China | — | — | — | — | — | — | — | No | — | — | — | No | — | — |  |
| 6 | p.Glu3Asp (p.E3D) | c.9G>C | Male | 33 | 23 | Yes | 421 | 84 | *HFE*: H63D/H63H *TMPRSS6*: T331M/ T331T *SUGP2*: R639Q/R639R | China | — | — | — | — | — | — | — | Yes | Yes | — | — | Yes | — | — | 3 |
| 7 | p.Glu3Asp (p.E3D) | c.9G>C | Female | 53 | 51 | Yes | 1402 | 49 | *BMP4*: R269Q/R269R *SUGP2*: R639Q/R639R | China | — | — | — | — | — | — | — | Yes | Yes | — | — | Yes | — | — | 3 |
| 8 | p.Glu3Asp (p.E3D) | c.9G>C | Male | 30 | — | No | 79.3 | 46 | *BMP4*: R269Q/R269R | China | — | — | — | — | — | — | — | No | — | — | — | No | — | — | 3 |
| 9 | p.His104Arg (p.H104R) | c.311A>G | Male | 46 | — | No | 266 | 56.2 | *TFR2*: A75V/A75A | China | — | — | — | — | — | — | — | — | — | — | — | — | — | — | 3 |
| 10 | p.Ile281Thr (p.I281T) | c.842T>C | Male | 49 | — | No | 554 | 34 |  | China | — | — | — | — | — | — | — | — | — | — | — | — | — | — | 4 |
| 11 | p.[Gln6His;Cys321Ter] (p.[Q6H;C321*]) | c.[18G>C;962_963delinsAA] | Male | 36 | 26 | Yes | >2000 | 60 |  | China | Yes | Yes | Yes | Yes | Yes | — | No | Yes | — | — | Yes | — | Phlebotomy, insulin | Die of sudden cardiac arrhythmia. | 5 |
| 12 | p.[Gln6His;Cys321Ter] (p.[Q6H;C321*]) | c.[18G>C;962_963delinsAA] | Male | 47 | 45 | No | >2000 | 40 |  | China | — | Yes | — | Yes | Yes | — | — | Yes | — | — | — | — | Phlebotomy | — | 5 |
| **Not described** | | | | | | | | | | | | | | | | | | | | | | | | | |
| 13 | p.Glu302Lys (p.E302K) | c.904G>A | Male | 61 | — | Yes | 356 | 68 | *HFE*: H63D/H63H | Brazil | — | — | — | — | — | — | — | — | — | — | — | — | — | — | 6 |
| 14 | p.Glu302Lys (p.E302K) | c.904G>A | Male | 65 | — | Yes | 1623 | 82 | *HFE*: H63D/H63H | Brazil | — | — | — | — | — | — | — | — | — | — | — | — | — | — | 6 |
| 15 | p.Ala310Gly (p.A310G) | c.929C>G | Female | 58 | — | Yes | — | — |  | Brazil | — | — | — | — | — | — | — | — | — | — | — | — | — | — | 6 |

HGVS, Human Genome Variation Society; *HJV*-HH, *HJV* related hereditary hemochromatosis.

**References:**

1. Pelusi S, Rametta R, Della CC, et al. Juvenile hemochromatosis associated with heterozygosity for novel hemojuvelin mutations and with unknown cofactors. Ann Hepatol 2014;13(5):568-71.

2. Biasiotto G, Roetto A, Daraio F, et al. Identification of new mutations of hepcidin and hemojuvelin in patients with HFE C282Y allele. Blood Cells Mol Dis 2004;33(3):338-43.

3. Lv T, Zhang W, Xu A, et al. Non-HFE mutations in haemochromatosis in China: combination of heterozygous mutations involving HJV signal peptide variants. J Med Genet 2018;55(10):650-60.

4. Huang FW, Rubio-Aliaga I, Kushner JP, Andrews NC, Fleming MD. Identification of a novel mutation (C321X) in HJV. Blood 2004;104(7):2176-7.

5. Li S, Xue J, Chen B, et al. Two middle-age-onset hemochromatosis patients with heterozygous mutations in the hemojuvelin gene in a Chinese family. Int J Hematol 2014;99(4):487-92.

6. Santos PC, Cancado RD, Pereira AC, et al. Hereditary hemochromatosis: mutations in genes involved in iron homeostasis in Brazilian patients. Blood Cells Mol Dis 2011;46(4):302-7.
